# Inhibiting Monoacylglycerol Lipase Suppresses RANKL-Induced Osteoclastogenesis and Alleviates Ovariectomy-Induced Bone Loss

**DOI:** 10.3389/fcell.2021.640867

**Published:** 2021-03-12

**Authors:** Hui Liu, Chuankun Zhou, Dahu Qi, Yutong Gao, Meipeng Zhu, Tenghui Tao, Xuying Sun, Jun Xiao

**Affiliations:** ^1^Department of Orthopedics, Tongji Hospital, Tongji Medical College, Huazhong University of Science and Technology, Wuhan, China; ^2^Department of Orthopedics Trauma and Microsurgery, Zhongnan Hospital of Wuhan University, Wuhan, China; ^3^Department of Pathology, Union Hospital, Tongji Medical College, Huazhong University of Science and Technology, Wuhan, China

**Keywords:** osteoporosis, monoacylglycerol lipase, JZL184, osteoclasts, receptor activator of nuclear factor (NF)-κB ligand (RANKL)

## Abstract

Osteoporosis is a common chronic metabolic bone disease characterized by reduced trabecular bone and increased bone fragility. Monoacylglycerol lipase (MAGL) is a lipolytic enzyme to catalyze the hydrolysis of monoglycerides and specifically degrades the 2-arachidonoyl glycerol (2-AG). Previous studies have identified that 2-AG is the mainly source for arachidonic acid and the most abundant endogenous agonist of cannabinoid receptors. Considering the close relationship between inflammatory mediators/cannabinoid receptors and bone metabolism, we speculated that MAGL may play a role in the osteoclast differentiation. In the present study, we found that MAGL protein expression increased during osteoclast differentiation. MAGL knockdown by adenovirus-mediated shRNA in bone marrow-derived macrophages demonstrated the suppressive effects of MAGL on osteoclast formation and bone resorption. In addition, pharmacological inhibition of MAGL by JZL184 suppressed osteoclast differentiation, bone resorption, and osteoclast-specific gene expression. Activation of the Mitogen-activated protein kinase (MAPK) and nuclear factor κB (NF-κB) pathways was inhibited by JZL184 and deletion of MAGL. Our *in vivo* study indicated that JZL184 ameliorated bone loss in an ovariectomized mouse model. Furthermore, overexpressing H1 calponin partially alleviated the inhibition caused by JZL184 or MAGL deletion on osteoclastogenesis. Therefore, we conclude that targeting MAGL may be a novel therapeutic strategy for osteoporosis.

## Introduction

Osteoporosis is a common chronic metabolic bone disease characterized by changes in bone mineral density and architecture (Henriksen et al., 2011; Rachner et al., 2011; Compston et al., 2019). The incidence of osteoporosis has increased annually with the aging of the population, particularly in postmenopausal women (Compston et al., 2019). Osteoporosis patients often experience a chronic course of disease, resulting in a high risk of bone fracture that severely affects quality of life.

The balance between bone formation by osteoblasts and bone resorption by osteoclasts continuously maintains bone homeostasis (Boyce, 2013). This process involves intricate factors, such as hormones, nutrition, drugs, and external biomechanical stimuli (Zhang et al., 2015). Changes in bone homeostasis can critically drive the progression of osteopathic diseases, such as inflammatory- or cancer-induced osteolysis, Paget’s disease, periodontitis, and osteoporosis (Liu et al., 2018; Mundy, 2007; Roodman and Windle, 2005; Zhang et al., 2011; Zhao et al., 2012). Osteoclasts, which are derived from the mononuclear/macrophage lineage, are exclusive giant bone-resorbing multinucleated cells with the capacity to resorb and degrade the mineralized bone matrix (Novack and Teitelbaum, 2008). Although a variety of factors are involved in osteoclast differentiation, macrophage colony-stimulating factor (M-CSF) and receptor activator of nuclear factor (NF)-κB ligand (RANKL) are two specific cytokines involved in the process. M-CSF mainly promotes survival, proliferation, and differentiation of the mononuclear/macrophage lineage, whereas RANKL provides signals essential for pre-osteoclasts to differentiate into mature osteoclasts (Kollet et al., 2007). RANKL binds to the RANK receptor on osteoclast progenitor cells to recruit downstream factors, such as NR receptor-associated factor 6, which activates nuclear factor κB (NF-κB), Mitogen-activated protein kinase (MAPK), and activator protein 1, which are important for the initial induction of the transcription factor nuclear factor of activated T-cell cytoplasmic 1 (NFATc1). NFATc1 translocates to the nucleus and cooperates with other transcription factors to activate the expression of osteoclast-specific genes, including tartrate-resistant acid phosphatase (TRAP), matrix metalloproteinase-9 (MMP-9), cathepsin K (CTSK), and c-FOS, which exert bone resorption functions (Nakashima et al., 2012). The current therapies for osteoporosis primarily target antiresorptive and anabolic agents, including bisphosphonates, calcitonin, estrogens, denosumab, and teriparatide (Rachner et al., 2011). Although these drugs alleviate symptoms, side effects should not be neglected. Therefore, suitable therapies must be developed.

Monoacylglycerol lipase (MAGL) is a member of the serine hydrolase family that functions as a lipolytic enzyme catalyzing the hydrolysis of monoglycerides into glycerol and fatty acids. MAGL specifically degrades endocannabinoid 2-arachidonoyl glycerol (2-AG) (Nomura et al., 2010; Grabner et al., 2017), a major source of arachidonic acid and a precursor of prostaglandins and other inflammatory mediators (Gil-Ordonez et al., 2018). In addition, 2-AG is the most abundant endogenous agonist for the cannabinoid receptor (Grabner et al., 2017). Studies have shown that inflammatory mediators are closely related to the process of osteoclast differentiation and bone resorption. Moreover, the Cannabinoid-type 1/Cannabinoid-type 2 (CB1/CB2) cannabinoid receptors are involved in bone homeostasis by regulating the differentiation of osteoclasts and osteoblasts (Bab and Zimmer, 2008; Galve-Roperh et al., 2013). Recent research showed that MAGL is closely related to various disorders, including cancers, pain, and neurodegenerative and inflammatory diseases (Gil-Ordonez et al., 2018). Tardelli et al. (2019) reported that inhibiting MAGL protects against liver injury and may be considered a potential therapy for sclerosing cholangitis. In addition, Xiang et al. (2018) reported that MAGL delays tumor progression by regulating activation of cannabinoid receptor 2-dependent macrophages. The latest study indicated that inhibiting MAGL protects against bone disease caused by primary bone cancer and bone metastasis (Marino et al., 2019). However, whether MAGL mediates osteoclastogenesis remains unknown.

The purpose of this study is to identify the role of MAGL in RANKL-induced osteoclast differentiation and bone resorption *in vitro* and in an ovariectomy (OVX)-induced osteoporosis mouse model *in vivo*, and to elucidate the underlying mechanisms.

## Materials and Methods

### Reagents and Antibodies

Recombinant murine soluble RANKL and M-CSF were purchased from R&D Systems (Minneapolis, MN, United States). JZL184 was purchased from Shanghai Lollane Biological Technology (Shanghai, China) and dissolved in DMSO (vehicle). Cell Counting Kit-8 (CCK8) was obtained from Boster Biotechnology (Wuhan, China). Antibodies against extracellular signal-regulated kinase (ERK), p-ERK, p38, p-p38, IκBα, p-IκBα, p65, p-p65, c-Jun N-terminal kinase (JNK), p-JNK, IκB kinase (IKK)-β, IKKα, p-IKKα/β, and c-Fos were purchased from Cell Signaling Technology (Beverly, MA, United States). Antibodies targeting tartrate-resistant acid phosphatase (TRAP), MMP-9, CTSK, and NFATc1 were acquired from Abcam (Cambridge, United Kingdom). The TRAP staining kit was purchased from Sigma-Aldrich (St. Louis, MO, United States). The Osteo-Assay Surface assay was obtained from Corning Life Sciences Inc., (Corning, NY, United States). Basal culture medium was obtained from Invitrogen (Carlsbad, CA, United States).

### Bone Marrow-Derived Macrophage Culture

Briefly, bone marrow macrophages (BMMs) were isolated from long bones and cultured for 24 h in α-MEM supplemented with 10% fetal bovine serum (FBS) and 30 ng/mL M-CSF (R&D Systems). After removing the adherent cells, the non-adherent cells were cultured for 3 days in the same medium, and the adherent cells were used as precursor cells for osteoclast differentiation.

### Cell Viability Assay

Bone marrow macrophages were plated at a density of 5 × 10^3^/well in triplicate in 96-well plates in α-MEM containing 10% FBS. The cells were incubated with vehicle or different concentrations of JZL184 (0–30 μM) for 24, 48, or 72 h. All tests were performed in triplicate, and cell viability was determined using the CCK-8 kit according to the manufacturer’s instructions. At the end of the incubation, 10 μL CCK-8 solution was added to each well, and the cells were incubated for 1 h. Absorbance was measured at 450 nm on the BioTek Absorbance Microplate Reader (BioTek, Winooski, VT, United States).

### Adenovirus-Mediated Knockdown and Plasmid Transfection

Adenoviruses carrying shRNA targeting mouse MAGL and control viruses were constructed by Vigene Biosciences (Rockville, MD, United States). Four shRNAs were ligated into one vector to target different MAGL regions. The sequences of each shRNA are listed in Supplementary Table 1. BMMs were seeded in a 12-well plate at the density of 4 × 10^4^ cells/well the day before transfection and infected with the virus for 12 h. Expression changes in MAGL were detected after 48 h. DDK-tagged H1 calponin (CNN1) plasmids and the control plasmid were obtained from OriGene Technologies (Rockville, MD, United States). The cells were transfected with a CNN1-encoding plasmid (OriGene, cat. no. MR219445) using Lipofectamine 3000 (Invitrogen) according to the manufacturer’s instructions.

### *In vitro* Osteoclastogenesis Assay and TRAP Staining

Bone marrow macrophages were seeded overnight in 96-well plates at a density of 2 × 10^4^/well. The following day, the cells were stimulated with or without JZL184 (0, 2, 4, 8, 10, or 20 μM) in the presence of M-CSF (30 ng/mL) and RANKL (75 ng/mL), or they were infected with MAGL-specific shRNA adenovirus or control adenovirus and then cultured with M-CSF and RANKL until osteoclasts formed. The multinucleated osteoclasts were fixed in 4% paraformaldehyde for 30 min and stained for TRAP enzymatic activity using a TRAP staining kit according to the manufacturer’s protocol. TRAP-positive cells with more than three nuclei were considered mature osteoclasts.

### F-Actin Ring Assays

Bone marrow macrophages were seeded on bone slices and treated with JZL184 (0, 2, 4, 8, 10, or 20 μM) or infected with MAGL-specific shRNA adenovirus or control adenovirus and cultured with M-CSF and RANKL until osteoclasts formed. The osteoclasts were fixed, permeabilized, blocked, stained with actin-tracker for 1 h, and counterstained with DAPI for 5 min. Images were captured, and formation of the actin ring and the number of average nuclei in these osteoclasts were analyzed using ImageJ software (National Institutes of Health, Bethesda, MD, United States) according to the mentioned detailed method (Chen et al., 2020a).

### Bone Resorption Pit Formation Assay

To determine whether MAGL affects osteoclast function, BMMs were seeded onto a 0.2% collagen gel-coated 6-well plate at a density of 5 × 10^5^/well and cultured with M-CSF and RANKL until osteoclasts formed. The osteoclasts were digested and seeded onto the Osteo-Assay surface in a multi-well plate. The cells were treated with the MAGL inhibitor JZL184 or with an adenovirus carrying MAGL-specific shRNA for 48 h in complete α-MEM containing RANKL (75 ng/mL) and M-CSF (30 ng/mL). Next, half of the wells per group were washed with disinfectant for 5 min and air-dried. Images of pit formation were captured using a fluorescence microscope and quantified using ImageJ software. The reabsorption area per well and the percentage reabsorption area per osteoclast were calculated to quantify osteoclast activity.

### RNA Extraction and qPCR

Total RNA was isolated from cultured BMMs using TRIzol reagent (Thermo Fisher Scientific, Waltham, MA, United States) according to the manufacturer’s instructions. mRNA expression was determined by qPCR as described in the SYBR Green Quantitative PCR Protocol (Takara, Shiga, Japan). The primer sequences used are as follows: GAPDH, 5′-ACGGGAAGC TCAC TGGCATGGCCTT-3′ (sense), 5′-CATGAGGTCCACCACCC TGTTGCTG-3′ (antisense); MAGL, 5′-CGGACTTCCAAGTT TTTGTCAGA-3′ (sense), 5′-GCAGCCACTAGGATGGAGA TG-3′ (antisense); NFATc1, 5′-CAACGCCCTGACCACCGA TAG-3′ (sense), 5′-GGGAAGTCAGAAGTGGGTGGA-3′ (antisense); TRAP, 5′-TACCTGTGTGGACATGACC-3′ (sense), 5′-CAGATCCATAGTGAAACCGC-3′ (antisense); CTSK, 5′-TGTATAACGCCACGGCAAA-3′ (sense), 5′-GGTTCACATT ATCACGGTCACA-3′ (antisense); MMP-9, 5′-TCCAGTACCA AGACAAAGCCTA-3′ (sense), 5′-TTGCACTGCACGGTTG AA-3′ (antisense); c-FOS, 5′-CGGGTTTCAACGCCGACTA-3′ (sense), 5′-TTGGCACTAGAGACGGACAGA-3′ (antisense).

### Western Blot Analysis

Cells were incubated in RIPA lysis buffer (Beyotime, Shanghai, China) supplemented with protease inhibitors and a phosphatase inhibitor cocktail (Boster Biotechnology) and centrifuged at 12,000 rpm for 30 min to isolate the supernatant. Proteins were separated by 10% sodium dodecyl sulfate–polyacrylamide gel electrophoresis and transferred to a polyvinylidene fluoride membrane. The membranes were blocked in 5% non-fat dry milk in Tris-buffered saline with 0.1% Tween 20 buffer (TBST) for 1 h at room temperature and incubated with the corresponding primary antibodies overnight at 4°C. After three washes in TBST, the membranes were incubated with the appropriate horseradish peroxidase-conjugated secondary antibodies for 1 h at room temperature. Finally, a chemiluminescence detection system (Thermo Fisher Scientific) and the ChemiDoc Touch Imaging System (Bio-Rad Laboratories, Hercules, CA, United States) were used to detect the labeled proteins. Protein expression levels were quantified using ImageJ software.

### Electrophoretic Mobility Shift Assay

Bone marrow macrophages were pretreated with vehicle or 20 μM JZL184 for 24 h and then stimulated with RANKL (75 ng/mL) for no more than 30 min. BMM nuclear extracts were prepared using a nuclear protein extraction kit (Beyotime, Jiangsu, China). Equal amounts of nuclear extract were incubated with an NF-κB probe at room temperature for 30 min. The reactant mixture was separated by 6.5% polyacrylamide gel electrophoresis and transferred to a positively charged nylon membrane. The membrane was cross-linked under ultraviolet light and then blocked and incubated with streptavidin–horseradish peroxidase conjugate. The image was detected using the ChemiDoc Touch Imaging System (Bio-Rad Laboratories).

### Immunofluorescence Analysis

Bone marrow macrophages were seeded in 24-well plates at a density of 1 × 10^5^/well and cultured for 24 h. After pretreatment with vehicle or 20 μM JZL184 for 12 h, the BMMs were stimulated with RANKL (75 ng/mL) for 30 min and then fixed, permeabilized, blocked, and incubated with an anti-p65 antibody overnight followed by incubation with secondary antibodies for 1 h. The nuclei were counterstained with DAPI for 5 min. Images were captured using a fluorescence microscope and analyzed the percentage of nuclear translocation positive cells and the average intensity in the nuclei according to the mentioned detailed method (Chen et al., 2020b).

### OVX-Induced Osteoporosis Mouse Model

Forty female C57BL/6 mice (12 weeks of age) were obtained from the Experimental Animal Center of Tongji Medical College (Wuhan, China) and randomly divided into four groups: sham group (*n* = 10), OVX group (*n* = 10), OVX + JZL low-dose group (*n* = 10), and OVX + JZL high-dose group (*n* = 10). Bilateral ovaries were removed to induce osteoporosis under anesthesia following an intraperitoneal injection of 1% pentobarbital sodium. The ovaries were only exposed during the sham surgery but not resected. After a 2-day recovery, the mice were intraperitoneally injected with JZL184 (8 or 16 mg/kg) or vehicle for 8 weeks. All animals were maintained under standard living conditions (25°C, 45–50% relative humidity, and 12/12-h dark/light cycle) in the Animal Research Center, Huazhong University of Science and Technology. All animal experimental procedures and care were approved by the Animal Ethics Committee of Tongji Hospital.

### Micro-CT (μCT) Scanning and Analysis

After the mice were sacrificed, the right femurs of mice in each group were fixed in 4% formaldehyde and scanned using high-resolution μCT (Scanco Medical, Bassersdorf, Switzerland). The scan parameters were a 100 kV voltage, 98 μA current, and a 10 μm voxel size. Three-dimensional images were reconstructed using the manufacturer’s software. The structural parameters, including bone volume per tissue volume, trabecular thickness, trabecular separation, and trabecular number, were analyzed to evaluate total bone mass.

### Histomorphometric Analysis

The left femurs of the mice in each group were fixed in 4% formaldehyde and decalcified in 10% EDTA. The femurs were embedded in paraffin blocks and sectioned at a 5-μm thickness on a microtome followed by hematoxylin and eosin (H&E) and TRAP staining. Images were captured using a fluorescence microscope.

### Immunocytochemical Analysis

Tissue samples were sectioned at 5 mm thickness. The sections were prepared for antigen restoration by immersion in 5% hydrogen peroxidase at 37°C for 10 min, and incubated overnight at 4°C with primary antibodies against MAGL (1:100) and p-P65 (1:100). The sections were incubated with a suitable secondary antibody and counterstained with hematoxylin.

### Enzyme-Linked Immunosorbent Assay

Mouse peripheral blood samples were collected and stored at −80°C until use. Serum RANKL, osteoprotegerin (OPG), tumor necrosis factor (TNF)-α, and interleukin (IL)-1β were measured using ELISA kits according to the manufacturer’s instructions (Boster Biotechnology).

### Statistical Analysis

All experimental data were collected from triplicate independently conducted experiments and presented as the mean ± standard deviation. The data were analyzed using the two-tailed Student’s *t*-test and analysis of variance. A *P*-value < 0.05 was considered significant.

## Results

### Knockdown of MAGL Reduces Osteoclast Formation and Bone Resorption

Monoacylglycerol lipase is a major enzyme involved in triglyceride degradation and arachidonic acid synthesis in particular tissues and has been considered a promising therapeutic target for treating diverse disorders, including autoimmune, cancer, neurodegenerative, and inflammatory diseases (Nomura et al., 2010; Jiang et al., 2015; Zhu et al., 2016; Pasquarelli et al., 2017). However, whether MAGL mediates osteoclastogenesis remains unknown.

**FIGURE 1:**
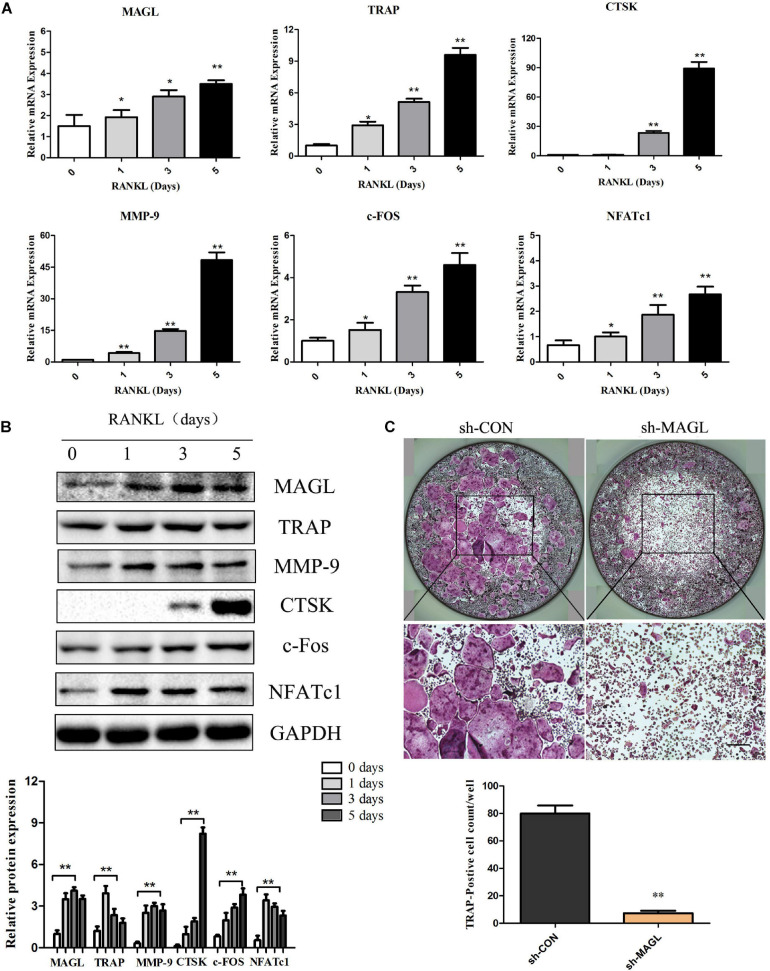
MAGL expression is regulated during RANKL-induced osteoclast differentiation. BMMs were treated with M-CSF (30 ng/ml) and RANKL (75 ng/ml) for the indicated times (0–5 days). **(A)** The mRNA expression of MAGL and osteoclast-specific genes, such as TRAP, CTSK, MMP9, c-FOS, and NFATc1, were determined by qPCR analysis. Data are presented as means ± SD of three independent experiments. **P < 0.01 vs. day 0. **(B)** Representative images of Western blots showing the expression of MAGL, TRAP, CTSK, MMP9, c-FOS, and NFATc1 in BMMs stimulated with RANKL for the indicated times. GAPDH served as the loading control for Western blot analysis. Data are presented as means ± SD of three independent experiments. **P < 0.01 vs. day 0. **(C)** BMMs were transfected with an adenovirus carrying MAGL-specific shRNA or a control adenovirus and cultured in the presence of M-CSF (30 ng/ml) and RANKL (75 ng/ml). The cells were subjected to TRAP staining, and TRAP-positive cells (≥3 nuclei) were counted. Data are presented as means ± SD of three independent experiments. *P < 0.05 and **P < 0.01 vs. the control adenovirus.

We evaluated the expression of MAGL during osteoclast differentiation to determine if MAGL is essential for osteoclastogenesis. BMMs were cultured with M-CSF and RANKL for up to 5 days, and the mRNA and protein levels of the majority of the osteoclast-specific genes, including TRAP, CTSK, MMP-9, c-FOS, and NFATc1 increased gradually during osteoclast differentiation (Figures 1A,B). MAGL mRNA expression and protein expression also increased during osteoclast differentiation (Figures 1A,B). To further elucidate the effect of MAGL during osteoclast differentiation, BMMs were infected with MAGL-specific shRNA adenovirus or control adenovirus to knockdown MAGL during osteoclast differentiation. The results indicated that MAGL knockdown significantly inhibited RANKL-induced osteoclast differentiation (Figure 1C).

**FIGURE 2:**
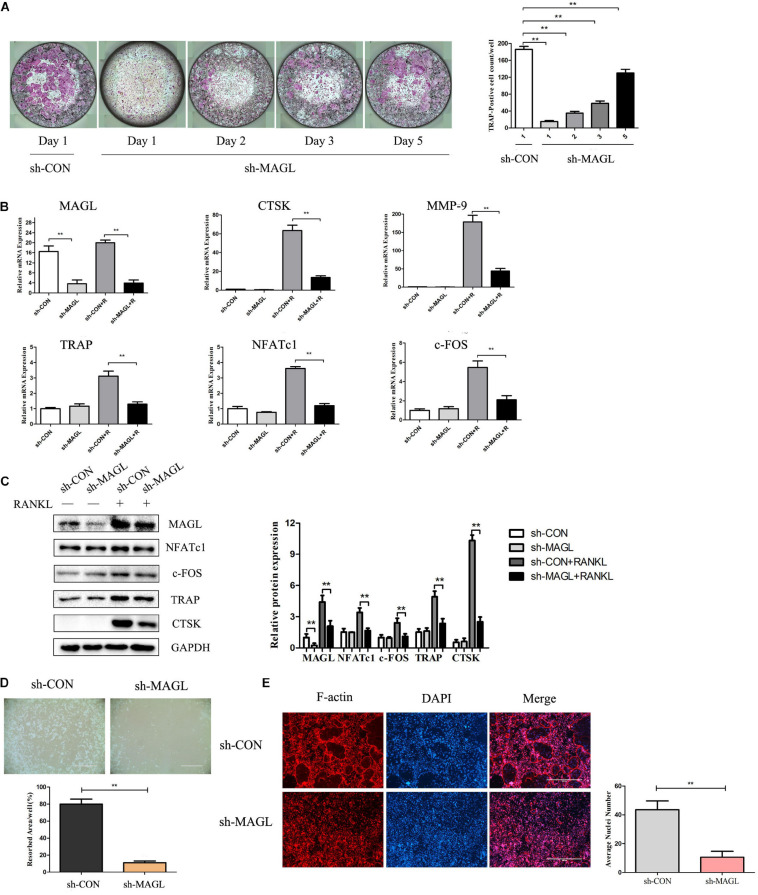
Knockdown of MAGL reduces osteoclast formation and bone resorption. BMMs were infected with adenovirus carrying MAGL-specific shRNA or a control adenovirus during osteoclast formation induced by M-CSF (30 ng/ml). **(A)** BMMs infected with control or sh-MAGL were stimulated with RANKL (75 ng/ml) and M-CSF (30 ng/ml) for the indicated times and analyzed for osteoclast formation. Representative images (left) and TRAP-positive cells (≥3 nuclei) were counted. Data are presented as means ± SD of three independent experiments. **P < 0.01 vs. day 0. **(B)** Control or sh-MAGL BMM cells were stimulated with RANKL (75 ng/ml) and M-CSF (30 ng/ml) for 3 days, and the expression of MAGL and osteoclast-specific genes was assessed by qPCR and **(C)** Western blotting. Data are presented as means ± SD of three independent experiments. **P < 0.01 vs. the control adenovirus. **(D)** Mature osteoclasts were seeded in Corning Osteo Assay strip wells and infected with control or sh-MAGL in the presence of RANKL (75 ng/ml) and M-CSF (30 ng/ml) for 3 days. Representative images of resorption pits are shown. **(E)** BMMs were infected with control or sh-MAGL and plated on bone slices in the presence of RANKL (75 ng/ml) and M-CSF (30 ng/ml) for 7 days. Representative F-actin images are shown. Data are presented as means ± SD of three independent experiments. **P < 0.01 vs. the control adenovirus.

We next analyzed MAGL knockdown at different time points during osteoclast differentiation to determine which differentiation stage was affected. The results demonstrated that MAGL knockdown suppressed osteoclast differentiation regardless of the differentiation stage, but that early phase knockdown of MAGL had a stronger inhibitory effect (Figure 2A). In addition, knockdown of MAGL significantly decreased mRNA and protein expression of osteoclast-specific genes, such as CTSK, TRAP, c-FOS, and NFATc1 (Figures 2B,C). The actin-rich sealing zone is an important structure for bone resorption. To further investigate the effect of MAGL on osteoclast resorption, we infected mature osteoclasts with MAGL-specific shRNA adenovirus and cultured them continuously with M-CSF and RANKL for 3 more days to evaluate the resorption activity of the osteoclasts. We observed a significant decrease in the bone resorption area in MAGL-knockdown mature osteoclasts (Figure 2D). Furthermore, knockdown of MAGL significantly inhibited RANKL-induced F-actin formation (Figure 2E).

### Pharmacological Inhibition of MAGL Suppresses RANKL-Induced Osteoclast Differentiation and Osteoclast-Specific Gene Expression

**FIGURE 3:**
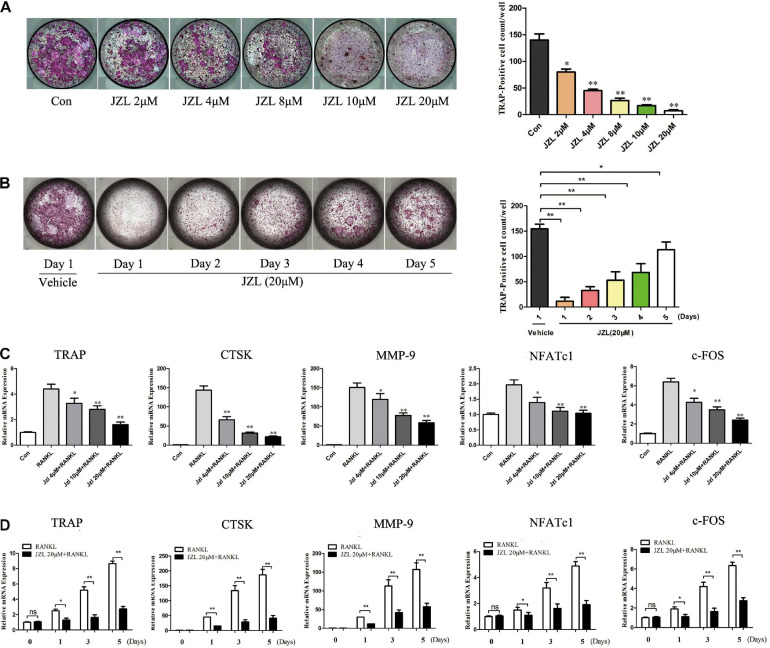
Inhibition of MAGL using JZL184 prevents osteoclast formation and osteoclast-specific gene expression. **(A)** BMMs were incubated with different concentrations of JZL184 or **(B)** stimulated with JZL184 (20 μM) for the indicated times in the presence of RANKL (75 ng/ml) and M-CSF (30 ng/ml). Representative images (left) and TRAP-positive cells (≥3 nuclei) were counted. Data are presented as means ± SD of three independent experiments. *P < 0.05 and **P < 0.01 vs. the vehicle. **(C)** BMMs were stimulated with different concentrations of JZL184 or **(D)** stimulated with JZL184 (20 μM) for the indicated times in the presence of M-CSF and RANKL for 3 days. The mRNA levels of osteoclast-related genes were assessed by qPCR. Data are presented as means ± SD of three independent experiments. *P < 0.05 and **P < 0.01 vs. the vehicle.

**FIGURE 4:**
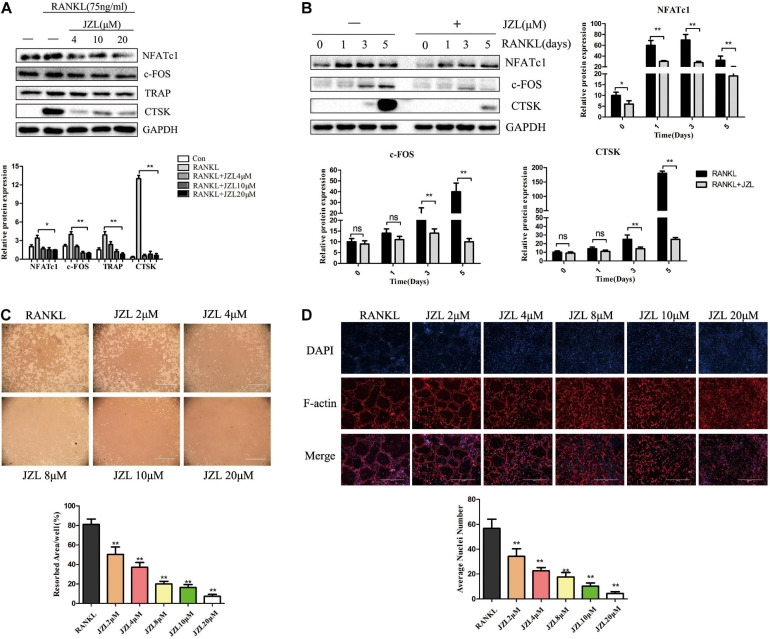
JZL184 inhibits RANKL-induced osteoclast-specific protein expression and osteoclast function. **(A)** BMMs were incubated with different concentrations of JZL184 or **(B)** stimulated with JZL184 (20 μM) for the indicated times in the presence of RANKL (75 ng/ml) and M-CSF (30 ng/ml), and the expression of osteoclast-specific protein was assessed by Western blotting. **(C)** Mature osteoclasts were seeded in Corning Osteo Assay strip wells and stimulated with different concentrations of JZL184 in the presence of RANKL (75 ng/ml) and M-CSF (30 ng/ml) for 3 days. Representative images of the resorption pits are shown. **(D)** BMMs stimulated with the indicated concentrations of JZL184 were plated on bone slices in the presence of RANKL (75 ng/ml) and M-CSF (30 ng/ml) for 7 days. Representative F-actin images are shown. Data are presented as means ± SD of three independent experiments. *P < 0.05 and **P < 0.01 vs. the vehicle.

The MAGL inhibitor JZL184 was applied to further determine whether inhibiting MAGL affects osteoclast differentiation. We determined the potential toxicity of JZL184 using CCK8 to measure BMM proliferation. JZL184 did not affect the proliferation of BMMs at doses < 20 μM (Supplementary Figure 1A). Next, we treated the BMMs with various concentrations of JZL184 (0, 2, 4, 8, 10, or 20 μM) to verify the role of MAGL in RANKL-induced osteoclast differentiation. TRAP staining revealed that JZL184 significantly suppressed osteoclast differentiation (Figure 3A). In addition, BMMs were treated with JZL184 at different time points, and the results indicated that JZL184 inhibited osteoclast differentiation mainly during the early phase (Figure 3B). We next examined the effect of JZL184 on osteoclast-specific gene expression. As shown in Figures 3C,D, the NFATc1 mRNA level was attenuated by JZL184. In addition, the NFATc1 downstream genes, including CTSK, MMP-9, TRAP, and c-FOS, were suppressed by JZL184. Our results also showed that JZL184 had strong concentration-dependent inhibitory effects on the osteoclast-specific protein expression of CTSK, MMP-9, and TRAP. The transcription factors NFATc1 and c-FOS were also suppressed dose-dependently by JZL184 treatment (Figures 4A,B).

### JZL184 Inhibits RANKL-Induced Osteoclast Function

We also evaluated the effects of JZL184 on osteoclast function. The results indicated that formation of the actin ring was significantly suppressed by JZL184 (Figure 4D). Furthermore, the hydroxyapatite resorption pit area per osteoclast decreased substantially in response to JZL184 treatment (Figure 4C). Together, these results suggest that JZL184 inhibits osteoclastic bone resorption *in vitro*.

### JZL184 Represses RANKL-Induced Activation of the NF-κB and MAPK Pathways

**FIGURE 5:**
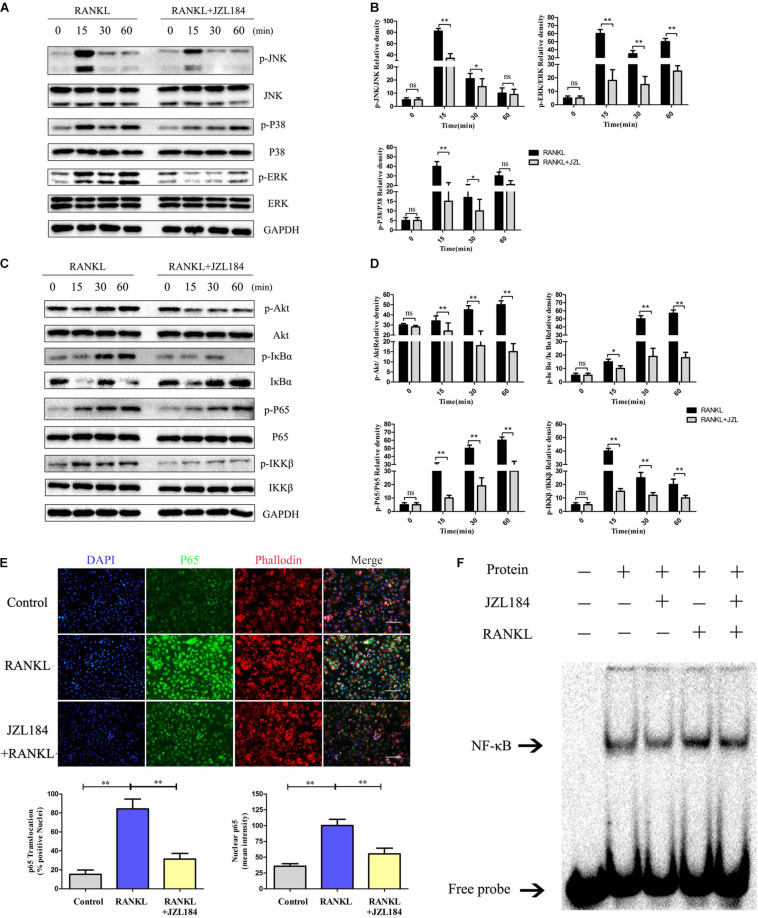
JZL184 represses RANKL-induced activation of the NF-κB and MAPK pathways. BMMs were cultured with α-MEM in the absence of FBS and pretreated with JZL184 (20 μM) or vehicle for 12 h. The BMMs were stimulated with or without RANKL (75 ng/mL) for the indicated times. **(A,B)** Total cell lysates were analyzed for cellular levels of p38, JNK, and ERK by Western blot analysis (left). **(C,D)** Total cell lysates were analyzed for levels of p65, IKKα/β, Akt, and IκBα by Western blot analysis (left). Right panel, densitometric analysis results from three independent experiments (right). Data are presented as means ± SD of three independent experiments. *P < 0.05 and **P < 0.01 vs. the vehicle. ns, not significant. **(E)** BMMs were pretreated with JZL184 (20 μM) for 12 h and then stimulated with RANKL for 30 min. Representative fluorescence micrographs of RANKL-induced translocation of p65 after JZL184 treatment are shown. **(F)** BMMs were pretreated with vehicle or 20 μM JZL184 for 24 h and stimulated with RANKL for 30 min. The nuclear proteins were prepared to evaluate binding of NF-κB to DNA by EMSA. Data are presented as means ± SD of three independent experiments. *P < 0.05 and **P < 0.01 vs. the vehicle or day 0.

The NF-κB and MAPK pathways were examined to further investigate whether JZL184 inhibits osteoclastogenesis. As shown in Figures 5A,B, phosphorylation of ERK, JNK, and p38 was significantly upregulated after RANKL stimulation. JZL184 treatment suppressed phosphorylated ERK, JNK, and p38 kinases after 15 min. Furthermore, phosphorylation of p65, IκBα, and IKKβ in the NF-κB signaling pathway was significantly repressed by JZL184 treatment (Figures 5C,D). The results indicated that JZL184 also partially suppressed RANKL-induced Akt phosphorylation. The immunofluorescence results revealed that JZL184 reduced RANKL-induced p65 nuclear translocation (Figure 5E). In addition, the EMSA results showed that the NF-κB DNA-binding capacity was partially suppressed by JZL184 (Figure 5F).

### Knockdown of MAGL Suppresses RANKL-Induced NF-κB and MAPK Activation

**FIGURE 6:**
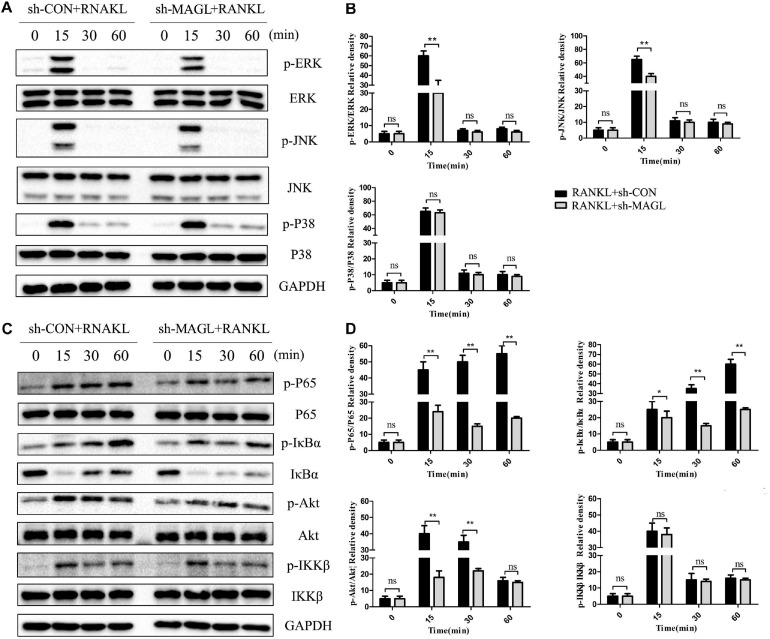
Knockdown of MAGL suppresses RANKL-induced NF-κB and MAPK activation. BMMs were infected with an adenovirus carrying MAGL-specific shRNA or a control adenovirus for 2 days and then cultured with α-MEM in the absence of FBS for 12 h. The BMMs were stimulated with or without RANKL (75 ng/mL) for the indicated times. **(A,B)** Total cell lysates were analyzed for levels of p38, JNK, and ERK by Western blot analysis (left). **(C,D)** Total cell lysates were analyzed for levels of p65, IKKα/β, Akt, and IκBα by Western blot analysis (left). Right panel, densitometric analysis results from three independent experiments (right). Data are presented as means ± SD of three independent experiments. *P < 0.05 and **P < 0.01 vs. the vehicle. ns, not significant.

We also evaluated whether deleting MAGL represses activation of the NF-κB and MAPK signaling pathways. Deleting MAGL decreased the phosphorylated ERK and JNK kinase levels after 15 min, whereas phosphorylation of p38 was not significantly affected (Figures 6A,B). Additionally, deleting MAGL inhibited p65, Akt, and IκB-α phosphorylation. However, IKKβ phosphorylation was not altered significantly after MAGL knockdown (Figures 6C,D). Although MAGL deletion and JZL184 treatment did not exactly have the same effects on family members of the MAPK and NF-κB pathways, they suppressed activation of the NF-κB and MAPK signaling pathways.

### JZL184 Ameliorates OVX-Induced Bone Loss

**FIGURE 7:**
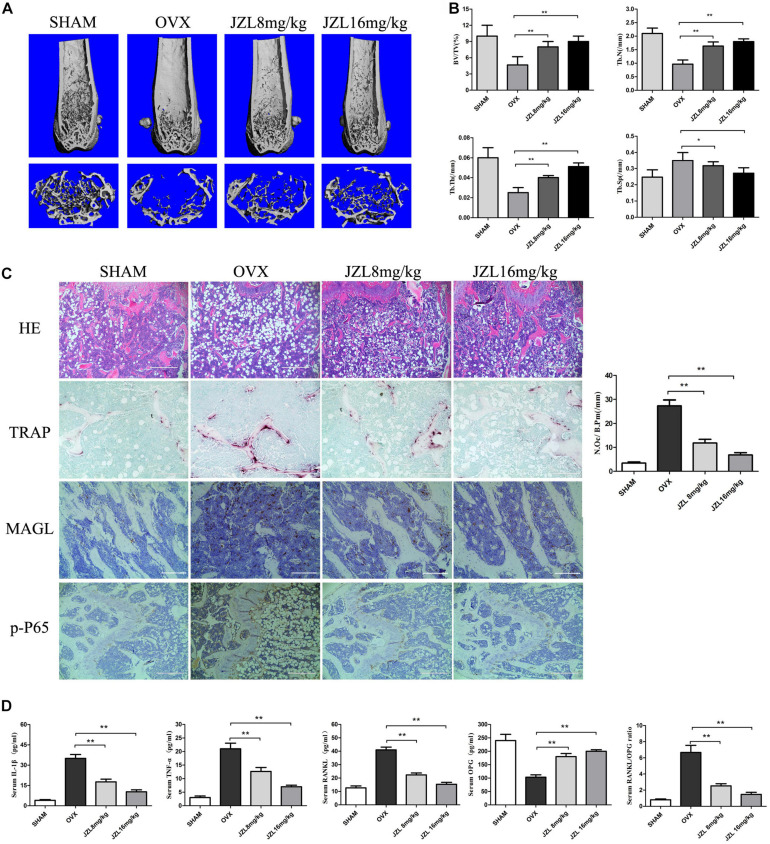
JZL184 ameliorates OVX-induced bone loss *in vivo*. Forty female C57BL/6 mice (12 weeks of age) were randomly divided into four groups: sham group (*n* = 10), OVX group (*n* = 10), OVX + JZL low-dose group (*n* = 10), and OVX + JZL high-dose group (*n* = 10). **(A)** The right femurs of all mice were scanned using high-resolution μCT, and representative 3D μCT reconstructed images are shown (scale bar = 1.00 mm) (*n* = 10). **(B)** Graphical representation of bone structural parameters, including bone volume/tissue volume (BV/TV), trabecular thickness (Tb.Th), trabecular number (Tb.N), and trabecular separation (Tb.Sp), are shown. **(C)** Sections of the left femurs of all mice were stained with H&E (scale bar = 400 μm), TRAP (scale bar = 200 μm), MAGL (scale bar = 200 μm), and p-P65 (scale bar = 400 μm). The number of osteoclasts per field of tissue (No. Oc/B.Pm) was counted. **(D)** Serum concentrations of RANKL, OPG, TNF-α, and IL-1β were measured. Data are presented as means ± SD. *P < 0.05, **P < 0.01 vs. the OVX group.

The above results demonstrated that inhibiting MAGL had a strong inhibitory effect on osteoclast formation and bone resorption. We further verified the ability of the MAGL inhibitor JZL184 to prevent OVX-induced osteoporosis *in vivo*. After injecting mice with either 8 or 16 mg/kg JZL184 or vehicle for 8 weeks postoperatively, they were euthanized and their right femurs scanned by μCT. The μCT images indicated that the bone trabecule of the OVX group were decreased significantly compared with the sham group, but this effect was rescued by JZL184 treatment, particularly high-dose JZL184. Additionally, JZL184 administration increased bone volume/tissue volume and trabecular number compared with those in the OVX mice (Figures 7A,B).

The histological analysis also demonstrated protective effects of JZL184 on OVX-induced bone loss. TRAP-stained sections showed that the number of TRAP + osteoclasts per bone surface area was decreased significantly in the distal femurs in the JZL184 group compared with the OVX group (Figure 7C). In addition, the IHC analysis showed that the expression of MAGL and p-P65 were decreased in the bone tissues of JZL84-treated OVX mice than in the OVX group (Figure 7C). We also evaluated the serum levels of RANKL, OPG, TNF-α, and IL-1β; the serum RANKL, TNF-α, and IL-1β levels were lower in the JZL184 group than in the OVX group, but the OPG level was higher in the JZL184 group than in the OVX group (Figure 7D).

### Overexpression of CNN1 Alleviates the Inhibitory Effects of JZL184 and MAGL Deletion on Osteoclastogenesis

**FIGURE 8:**
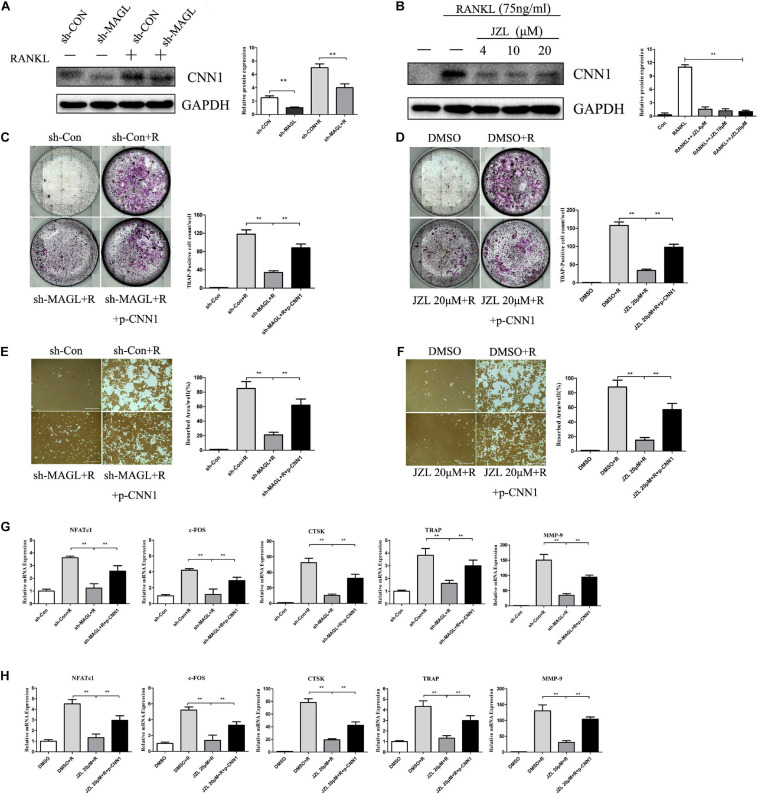
Overexpression of CNN1 alleviates the inhibitory effects of JZL184 or MAGL deletion on osteoclast formation and bone resorption. BMMs were transfected with a CNN1-overexpressing plasmid and then infected with sh-MAGL or pretreated with JZL184. The BMMs were stimulated with or without RANKL (75 ng/mL). **(A,B)** Total cell lysates were analyzed for CNN1 by Western blot analysis. **(C,D)** Representative images and TRAP-positive cells (≥3 nuclei) were counted. **(E,F)** Representative images of resorption pits are shown. **(G,H)** Total cell lysates were analyzed for the levels of osteoclast-specific genes by qPCR. Data are presented as means ± SD of three independent experiments. **P < 0.01 vs. the vehicle or the control adenovirus or sh-MAGL group.

CNN1 is an important smooth muscle-specific actin-binding protein (Taniguchi, 2005). Previous studies have shown that overexpressing CNN1 in osteoblast lineage cells promotes the formation of osteoclasts (Su et al., 2013). We found that CNN1 expression was upregulated after RANKL stimulation, but this effect was inhibited by JZL184 treatment and MAGL deletion (Figures 8A,B). Next, we assessed the effect of CNN1 overexpression in BMMs treated with JZL184 or deleted for MAGL by transfecting the cells with a plasmid expressing mouse CNN1. Overexpression of CNN1 alleviated the inhibitory effects of JZL184 or MAGL deletion on osteoclast differentiation (Figures 8C,D). Moreover, overexpressing CNN1 alleviated the inhibitory effect of JZL184 or MAGL deletion on bone resorption (Figures 8E,F). We also demonstrated that overexpressing CNN1 mitigated the inhibitory effect of JZL184 or MAGL deletion on the expression of osteoclast-specific genes, including NFATc1, CTSK, MMP-9, TRAP, and c-FOS (Figures 8G,H). We conclude that MAGL is essential for osteoclastogenesis partially via regulation of CNN1 expression.

## Discussion

Osteoporosis is an increasingly common chronic metabolic bone disease, characterized by reduced trabecular bone and increased bone fragility, that occurs frequently in developed and developing countries (Binder et al., 2009). The balance between bone formation by osteoblasts and bone resorption by osteoclasts is essential for healthy bone growth and maintenance. However, a variety of factors, such as hormones and cytokines, activate osteoclast activity, which in turn causes progressive destruction of bone mass and structure. Clinical therapies for osteoporosis have focused primarily on anti-resorptive and anabolic agents. Although these agents alleviate some symptoms, the side effects, such as breast cancer and atypical femoral fractures, are receiving increasing attention (Andreopoulou and Bockman, 2015; Chan et al., 2016).

In this study, we investigated the role of MAGL in osteoclast differentiation and function. We found that expression of MAGL protein was increased during osteoclast differentiation. Knocking down MAGL via adenovirus-mediated shRNA demonstrated a suppressive effect on osteoclast formation and bone resorption. In addition, pharmacological inhibition of MAGL using JZL184 suppressed osteoclast differentiation in a dose-dependent manner. Activation of the MAPK and NF-κB pathways was inhibited by the MAGL inhibitor JZL184 or by MAGL deletion. The MAGL inhibitor ameliorated bone loss in the OVX mouse model, suggesting that a decreased level or impaired enzymatic activity of MAGL may increase bone density in menopausal women. Furthermore, overexpressing CNN1 partially alleviated the inhibitory effect of JZL184 or MAGL deletion on osteoclastogenesis.

Monoacylglycerol lipase is a lipolytic enzyme that catalyzes the hydrolysis of monoglycerides into glycerol and fatty acids and specifically degrades 2-AG (Nomura et al., 2011). Several studies have reported that 2-AG is the main source of arachidonic acid. In addition, 2-AG is the most abundant endogenous agonist of the cannabinoid receptor (Grabner et al., 2017; Turcotte et al., 2015). Considering the close relationship between inflammatory mediators/cannabinoid receptors and bone metabolism, we further investigated whether MAGL is involved in osteoclast differentiation. Therefore, we knocked down MAGL by adenovirus-mediated shRNA in BMMs during osteoclast differentiation, and the results of TRAP staining, F-actin belts, and hydroxyapatite resorption assays demonstrated that MAGL knockdown significantly inhibited osteoclast differentiation and resorption. Moreover, pharmacological inhibition of MAGL using JZL184 had consistent inhibitory effects on osteoclast differentiation and function. These results suggest that MAGL is essential for RANKL-induced osteoclastogenesis and bone resorption activity.

Nuclear factor of activated T-cell cytoplasmic 1 has been demonstrated to be a key transcription factor involved in osteoclast differentiation, cooperating with other transcription factors to activate the expression of osteoclast-specific genes (Asagiri and Takayanagi, 2007; Takayanagi, 2007). We investigated the effect of MAGL on NFATc1. Treatment with the MAGL inhibitor or knockdown of MAGL repressed NFATc1 expression. Furthermore, osteoclast marker genes, including c-FOS, CTSK, TRAP, and MMP-9, which are regulated by NFATc1, decreased in response to JZL184 and MAGL knockdown.

Osteoclastogenesis is regulated by complex signaling cascades and by the NF-κB and MAPK signaling pathways (Park-Min, 2018). Activation of the NF-κB transcription factor is required for sufficient osteoclast differentiation (Abu-Amer, 2013). Accumulating studies have shown strong correlations of MAGL with MAPK and NF-κB pathways. Zhu et al. reported that MAGL promotes the progression of hepatocellular carcinoma via the NF-κB-mediated epithelial–mesenchymal transition (Zhu et al., 2016). Here, pretreatment with JZL184 suppressed the DNA-binding capacity of NF-κB and inhibited RANKL-induced phosphorylation of IKK and IκBα, as well as translocation of nuclear p65. In addition, JZL184 attenuated phosphorylation of ERK, JNK, p38, and Akt, and knockdown of MAGL suppressed RANKL-induced NF-κB and MAPK activation. However, MAGL knockdown and JZL184 did not have the same effects on family members of the MAPK and NF-κB pathways; thus, we speculate that the MAGL inhibitor JZL184 may have some MAGL-independent effects.

CNN1 is an actin filament-associated regulatory protein. Calponin proteins have three isoforms, of which CNN1 is an important smooth muscle-specific protein, whereas CNN2 is expressed mainly in smooth muscle and certain non-muscle cells (Liu and Jin, 2016). Studies have shown that CNN1 is closely related to various cellular physiological processes, including differentiation and apoptosis (Wang et al., 2013; Zhang et al., 2016). Feng et al. (2019) demonstrated that CNN1 and CNN2 play important roles in smooth muscle, and that double deletion of these genes decreases systemic blood pressure by blunting the length–tension response of aortic smooth muscle. One study founded that overexpressing CNN1 in Col1a1-Cnn1 transgenic mice inhibited bone formation at embryonic stage and decreased bone mass at adult stage. In addition to impaired bone formation, the decreased bone mass was also associated with enhanced osteoclastogenesis. TRAP staining revealed the number of osteoclast numbers increased in tibias of 2-month-old Col1a1-Cnn1 mice (Su et al., 2013). In our study, we found that CNN1 expression was unregulated after stimulating BMMs with RANKL, but this effect was inhibited by JZL184 or MAGL deletion. So, we speculated that CNN1 played an active role in osteoclasts and may be a downstream gene of MAGL. Therefore, we performed the CNN1 overexpression in BMMs and attempted to verify whether inhibiting MAGL suppress RANKL-induced osteoclastogenesis by regulating CNN1. The results indicated that overexpressing CNN1 alleviated the inhibitory effects of JZL184 or MAGL deletion on osteoclast differentiation and bone resorption. In addition, overexpressing CNN1 mitigated the inhibitory effect of JZL184 or MAGL deletion on the expression of osteoclast-specific genes. Thus, we concluded that MAGL is essential for osteoclastogenesis, in part by regulating CNN1 expression.

We further verified the potential of the MAGL inhibitor JZL184 to prevent OVX-induced osteoporosis *in vivo*. The μCT and H&E staining results suggested that JZL184 alleviated OVX-induced bone loss in a mouse model. Additionally, the TRAP staining results indicated that osteoclast formation and function decreased in response to JZL184 treatment, which was consistent with the *in vitro* study. Furthermore, there were no significant histopathological alterations in the liver and kidney of jzl184-treated mice compared with vehicle-treated mice (Supplementary Figure 2).

This study had some limitations. We used the MAGL inhibitor JZL184 rather than MAGL conditional knockout mice to verify the effect of MAGL activity in the mouse model of OVX-induced bone loss. We plan to further evaluate the effect of MAGL activity on osteogenesis.

**FIGURE 9:**
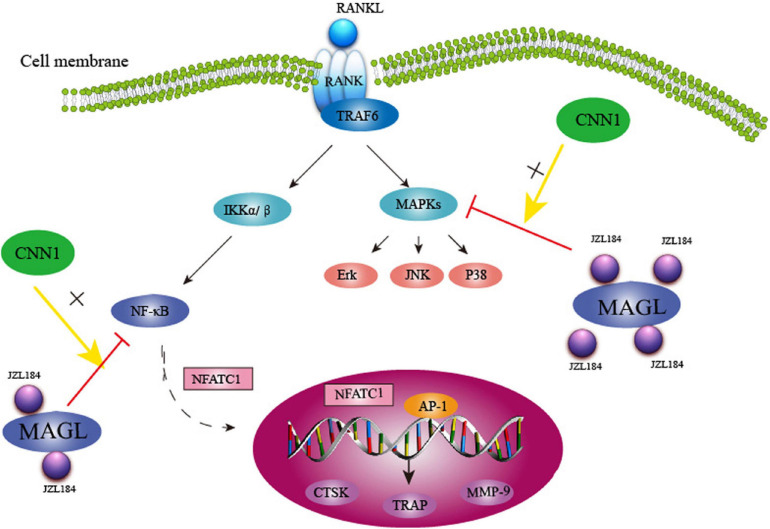
Schematic representation of MAGL regulates RANKL-induced osteoclastogenesis and alleviates ovariectomy-induced bone loss.

Collectively, our findings show that MAGL plays a critical role in RANKL-induced osteoclast formation and bone resorption. Pharmacological inhibition or knockdown of MAGL suppressed RANKL-induced osteoclastogenesis by suppressing MAPK, NF-κB, and CNN1 activities *in vitro* and alleviating OVX-induced bone loss *in vivo*(Figure 9). These results suggest that targeting MAGL may be a novel therapeutic strategy for osteoclast-associated diseases.

## Data Availability Statement

The original contributions presented in the study are included in the manuscript/Supplementary Material, further inquiries can be directed to the corresponding author/s.

## Ethics Statement

The animal study was reviewed and approved by the Animal Ethics Committee of Tongji Hospital.

## Author Contributions

HL performed the experiments and analyzed the data and produced the initial draft of the manuscript. CZ, DQ, YG, MZ, TT, and XS analyzed the data. JX designed the study and revised the manuscript. All authors have read and approved the final submitted manuscript.

## Conflict of Interest

The authors declare that the research was conducted in the absence of any commercial or financial relationships that could be construed as a potential conflict of interest.
