# Supplementary material for: Inhibiting Monoacylglycerol Lipase Suppresses RANKL-Induced Osteoclastogenesis and Alleviates Ovariectomy-Induced Bone Loss
Source: Front Cell Dev Biol. 2021 Mar 12;9:640867. doi: 10.3389/fcell.2021.640867 (PMC7994615; doi:10.3389/fcell.2021.640867)
Supplement: Supplementary file 1 [file Data_Sheet_1.zip › Rename our supplementary files/Supplemental Figure 1.The effect of JZL184 on the proliferation of BMM cells..docx]

**
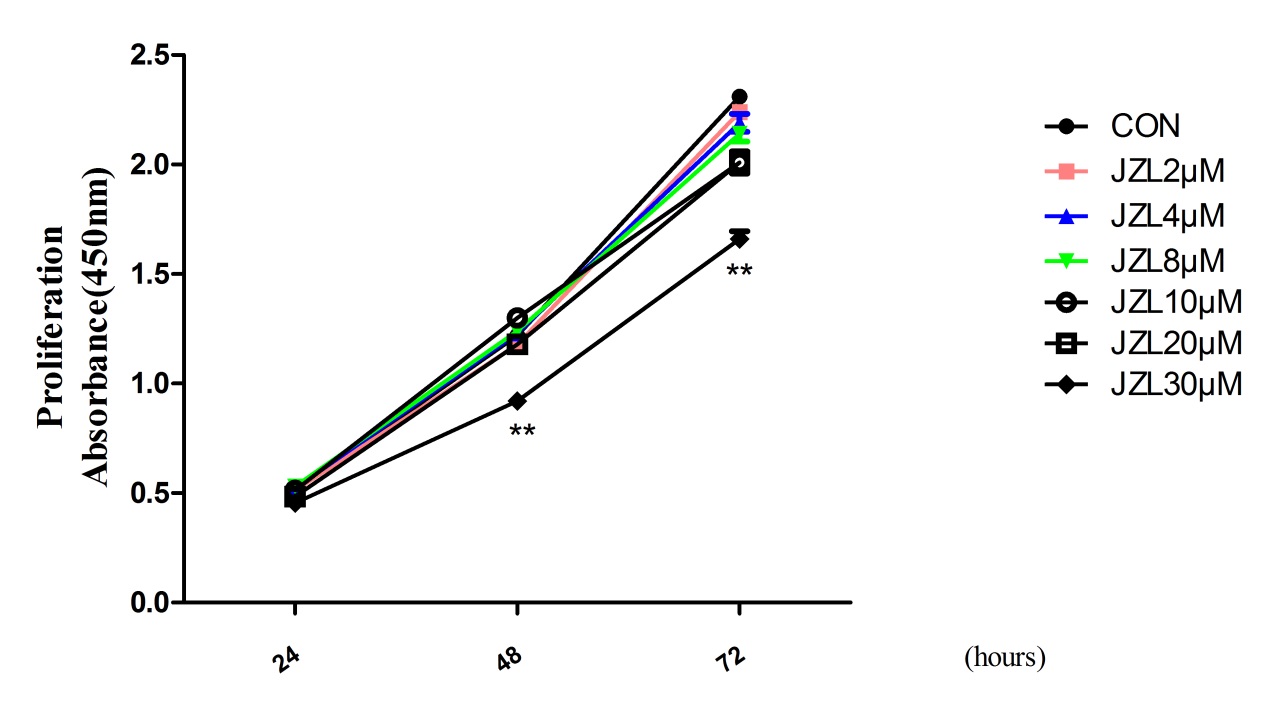
**

**Supplemental Fig 1.** BMM cells were treated with various concentrations of JZL184 (0,2,4, 8,10,20 and 30 μM) for different periods of time (24, 48, and 72 hours). BMM cells proliferation was assessed using CCK-8 at 24, 48, and 72 hours days after treatment. JZL184 did not significantly inhibit the proliferation of BMM cells at doses below 20 μM.
