# Supplementary material for: Inhibiting Monoacylglycerol Lipase Suppresses RANKL-Induced Osteoclastogenesis and Alleviates Ovariectomy-Induced Bone Loss
Source: Front Cell Dev Biol. 2021 Mar 12;9:640867. doi: 10.3389/fcell.2021.640867 (PMC7994615; doi:10.3389/fcell.2021.640867)
Supplement: Supplementary file 1 [file Data_Sheet_1.zip › Rename our supplementary files/Supplemental Figure 2. The effect of JZL184 on the liver and kidney of OVX mice..docx]

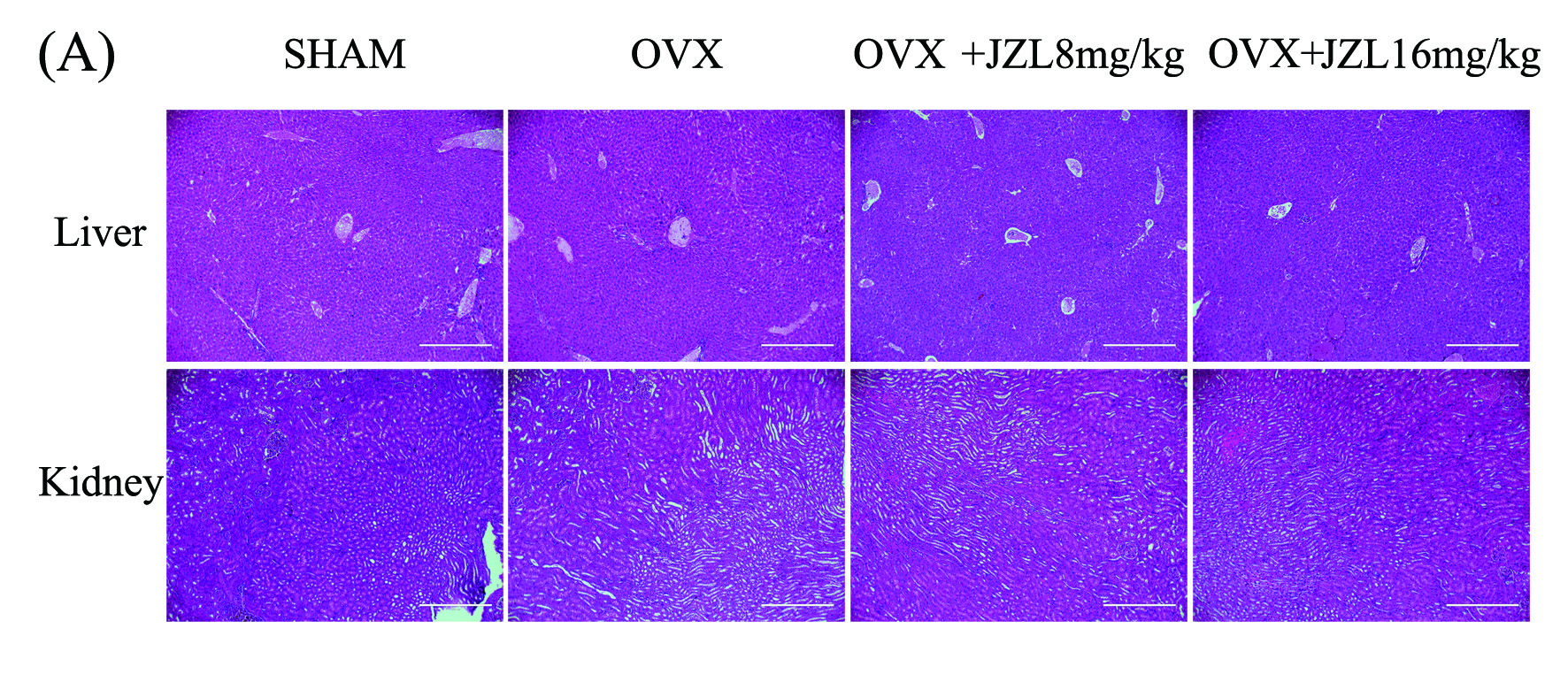


**Supplemental Fig 2. Effect of JZL184 on the liver and kidney of OVX mice.** Photomicrographs show the histopathology of the liver and kidney from Vehicle- or JZL184-treated mice (scale bar = 400 μm).
