# Supplementary material for: Inhibiting Monoacylglycerol Lipase Suppresses RANKL-Induced Osteoclastogenesis and Alleviates Ovariectomy-Induced Bone Loss
Source: Front Cell Dev Biol. 2021 Mar 12;9:640867. doi: 10.3389/fcell.2021.640867 (PMC7994615; doi:10.3389/fcell.2021.640867)
Supplement: Supplementary file 1 [file Data_Sheet_1.zip › Rename our supplementary files/Supplemental Figure 3.The knockdown efficiency of MAGL in BMM cells.docx]

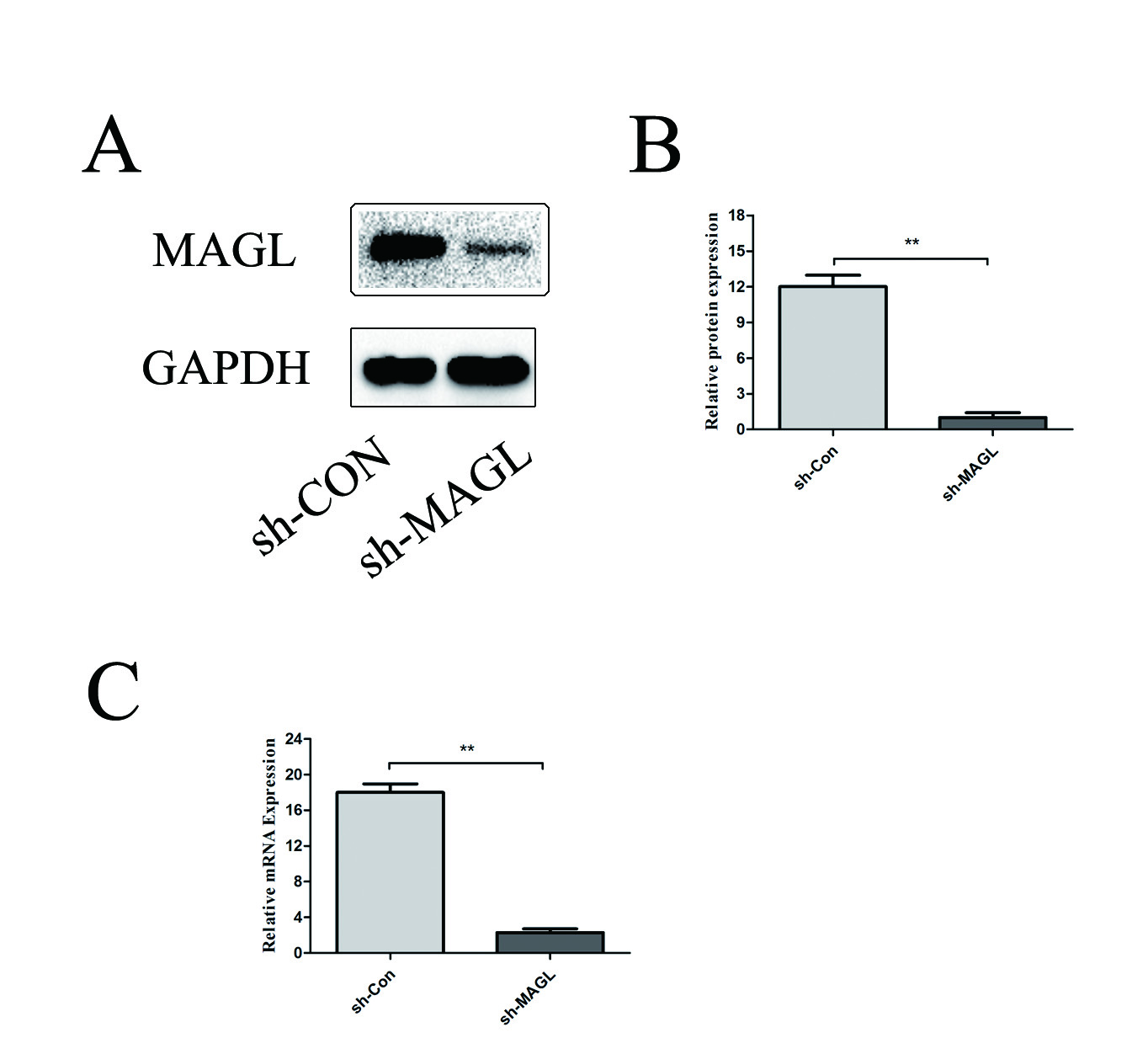


**Supplemental Fig 3.** BMMs were seeded in a 12-well plate at the density of 4 × 10^4^ cells/well the day before transfection and infected with the virus for 12 h. Expression changes in MAGL protein (A and B) and m RNA (C) were detected after 48 h.
