# Supplementary material for: Inhibiting Monoacylglycerol Lipase Suppresses RANKL-Induced Osteoclastogenesis and Alleviates Ovariectomy-Induced Bone Loss
Source: Front Cell Dev Biol. 2021 Mar 12;9:640867. doi: 10.3389/fcell.2021.640867 (PMC7994615; doi:10.3389/fcell.2021.640867)
Supplement: Supplementary file 1 [file Data_Sheet_1.zip › Rename our supplementary files/Supplemental Figure 4. The uncropped images of the original western blots..pdf]

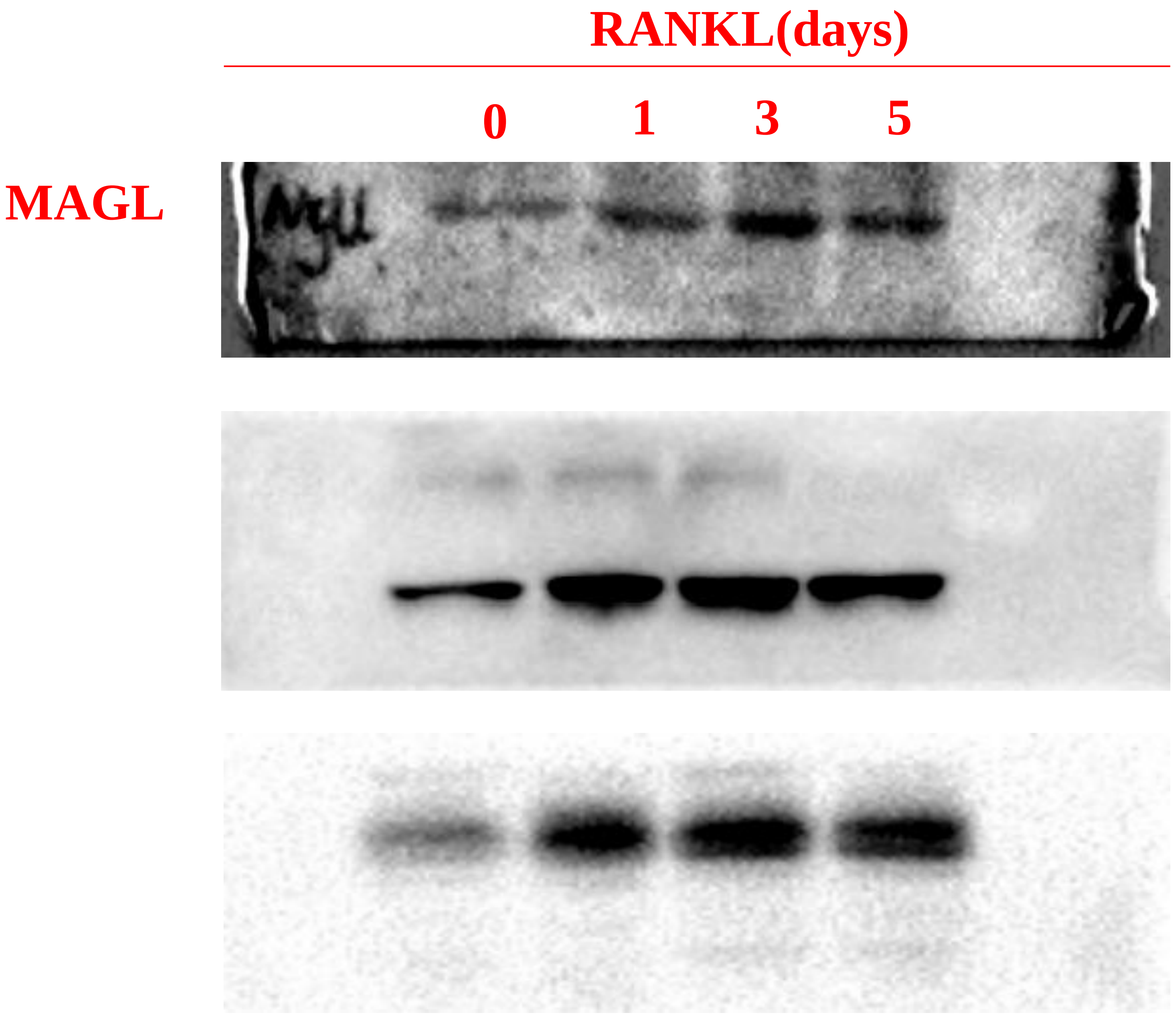
RANKL(days)
0
1
3
5
MAGL

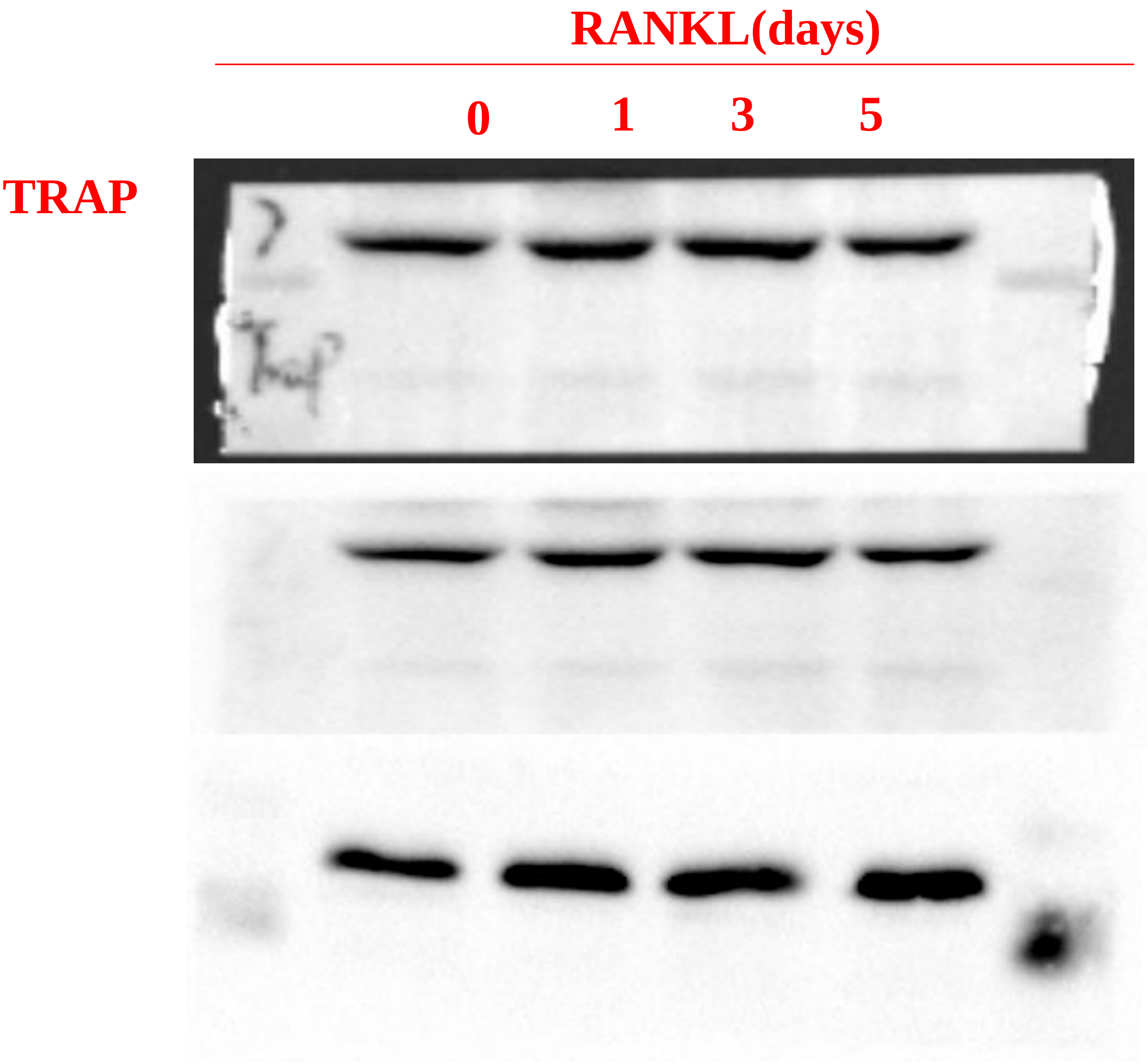
RANKL(days)
0
1
3
5
TRAP

RANKL(days)

0 1 3 5

CTSK

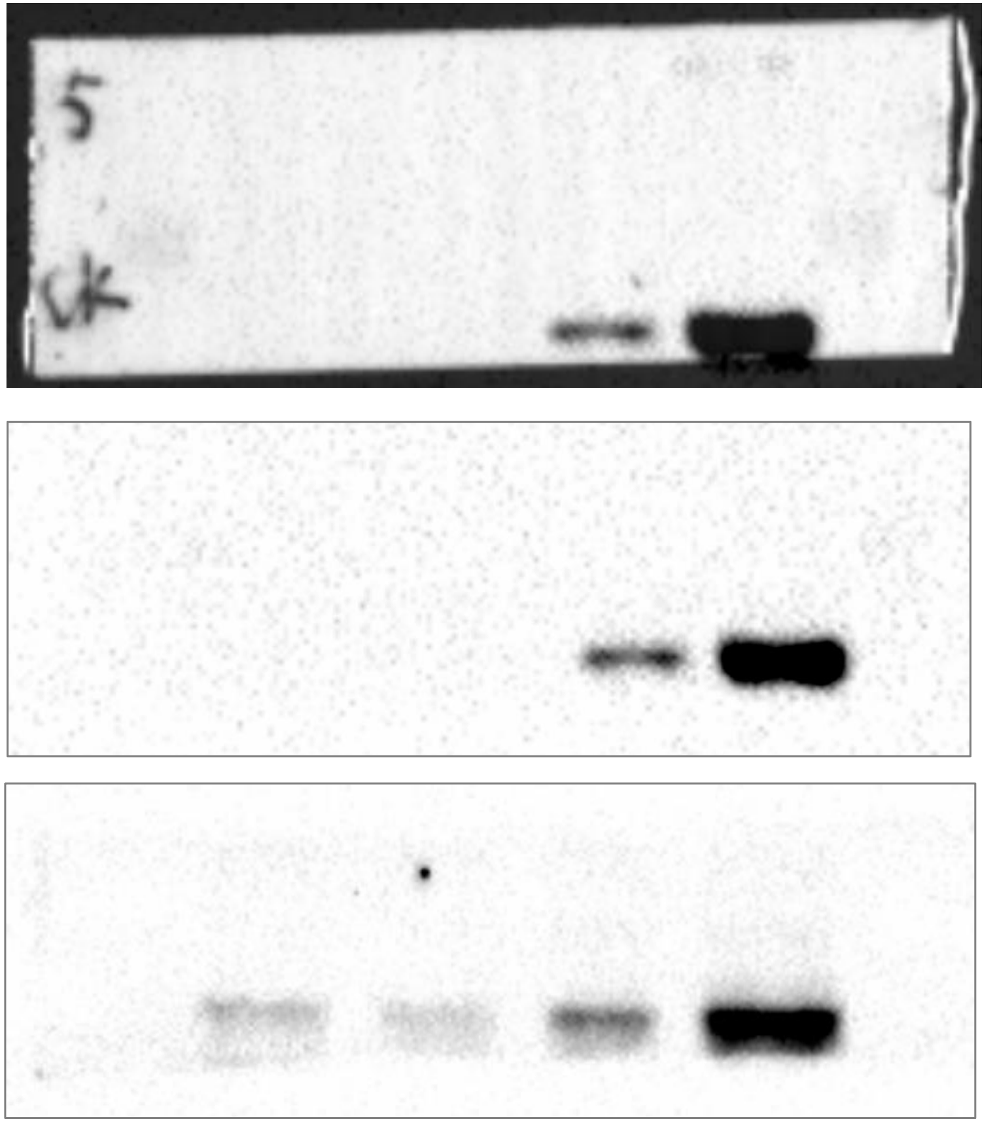

RANKL(days)

0 1 3 5

c-FOS

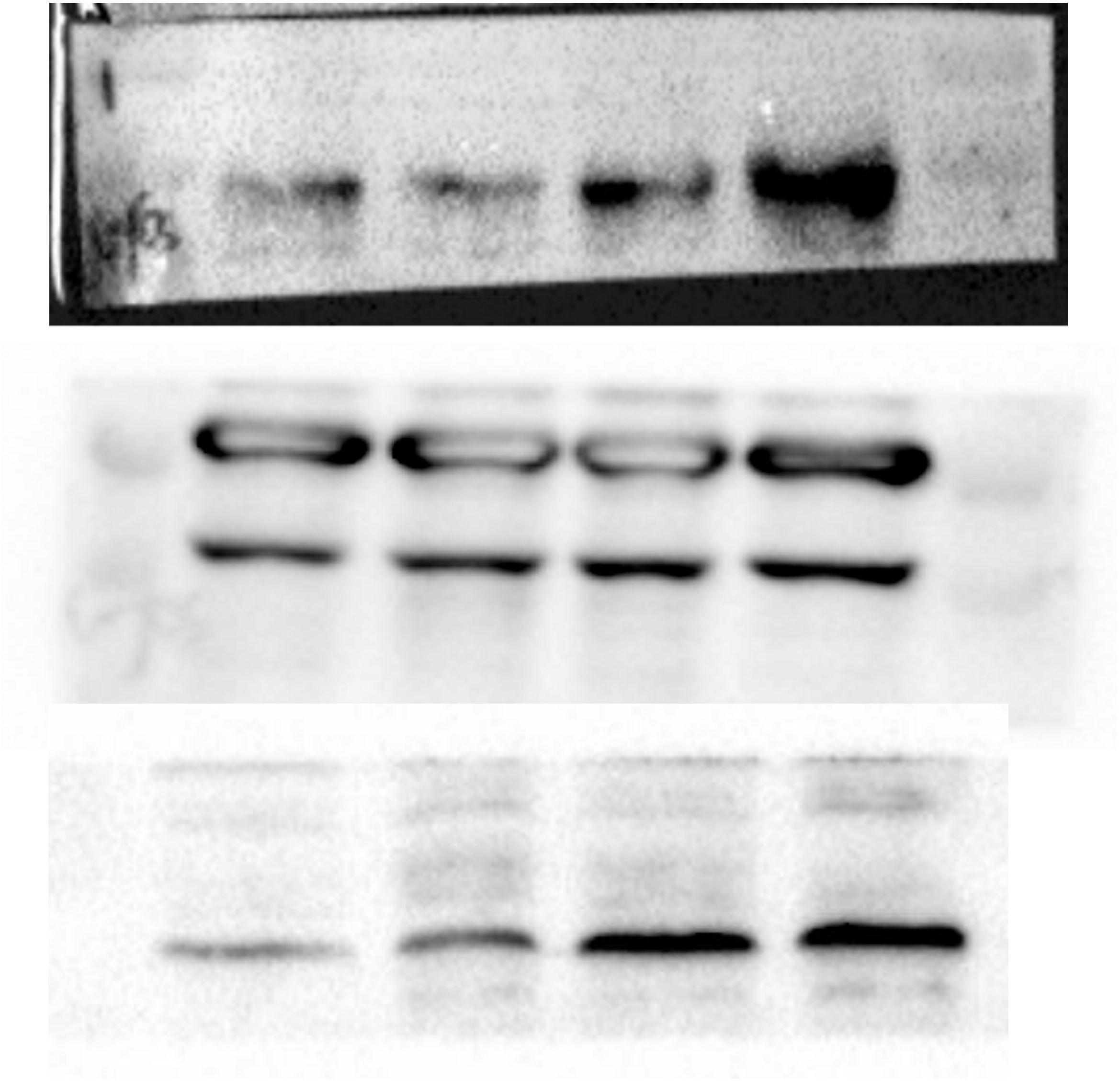

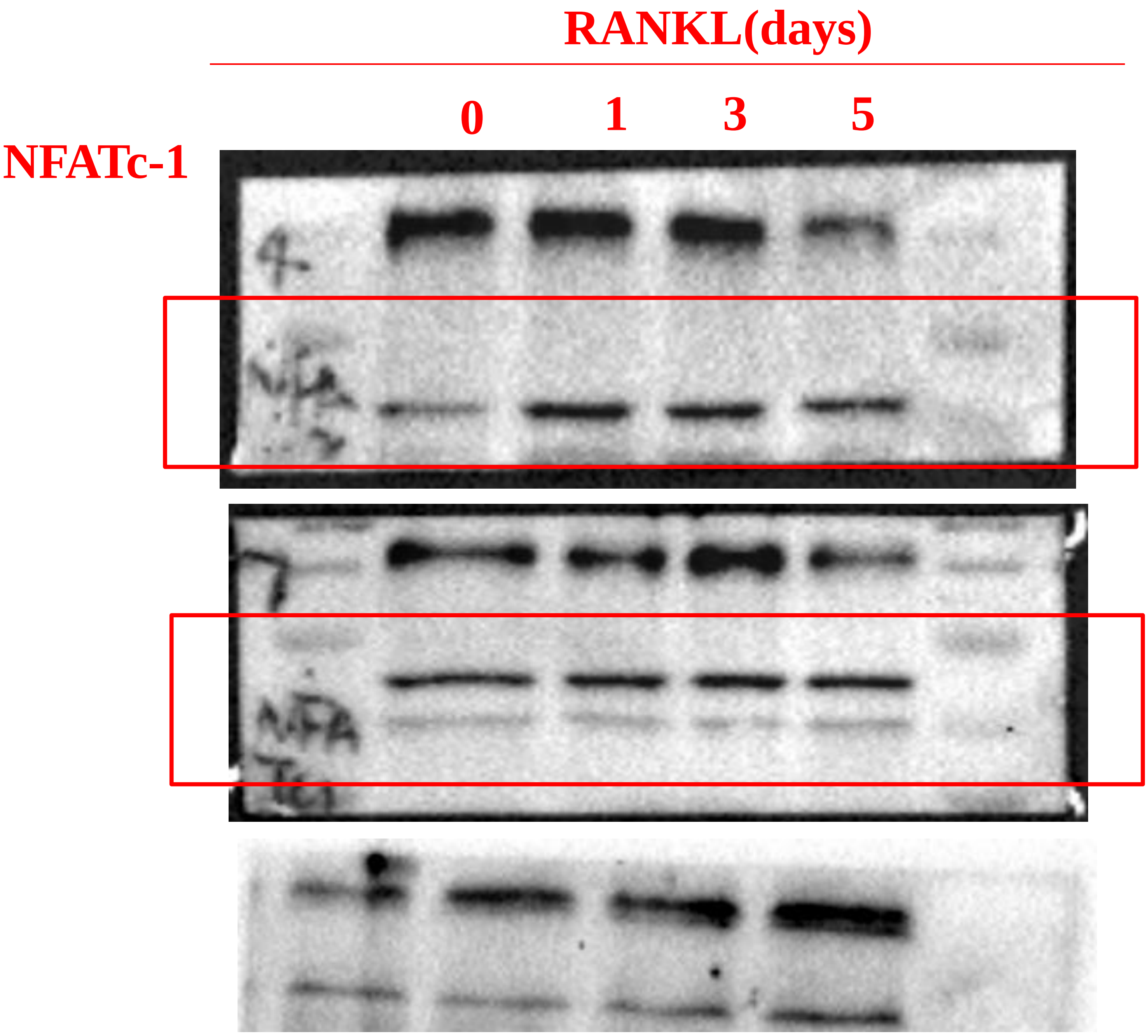

RANKL(days)
0
1
3
5
NFATc-1

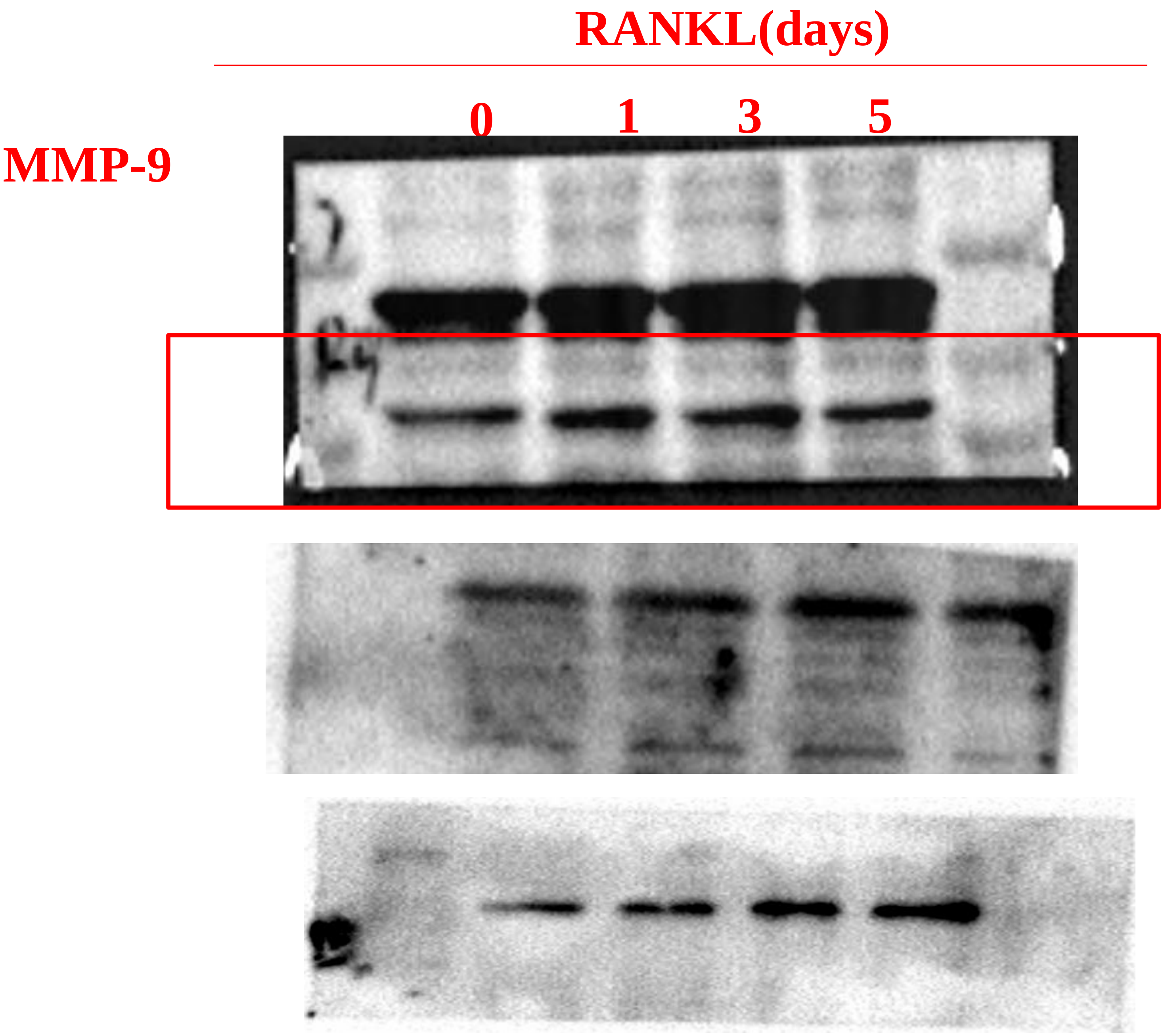
RANKL(days)
0
1
3
5
MMP-9

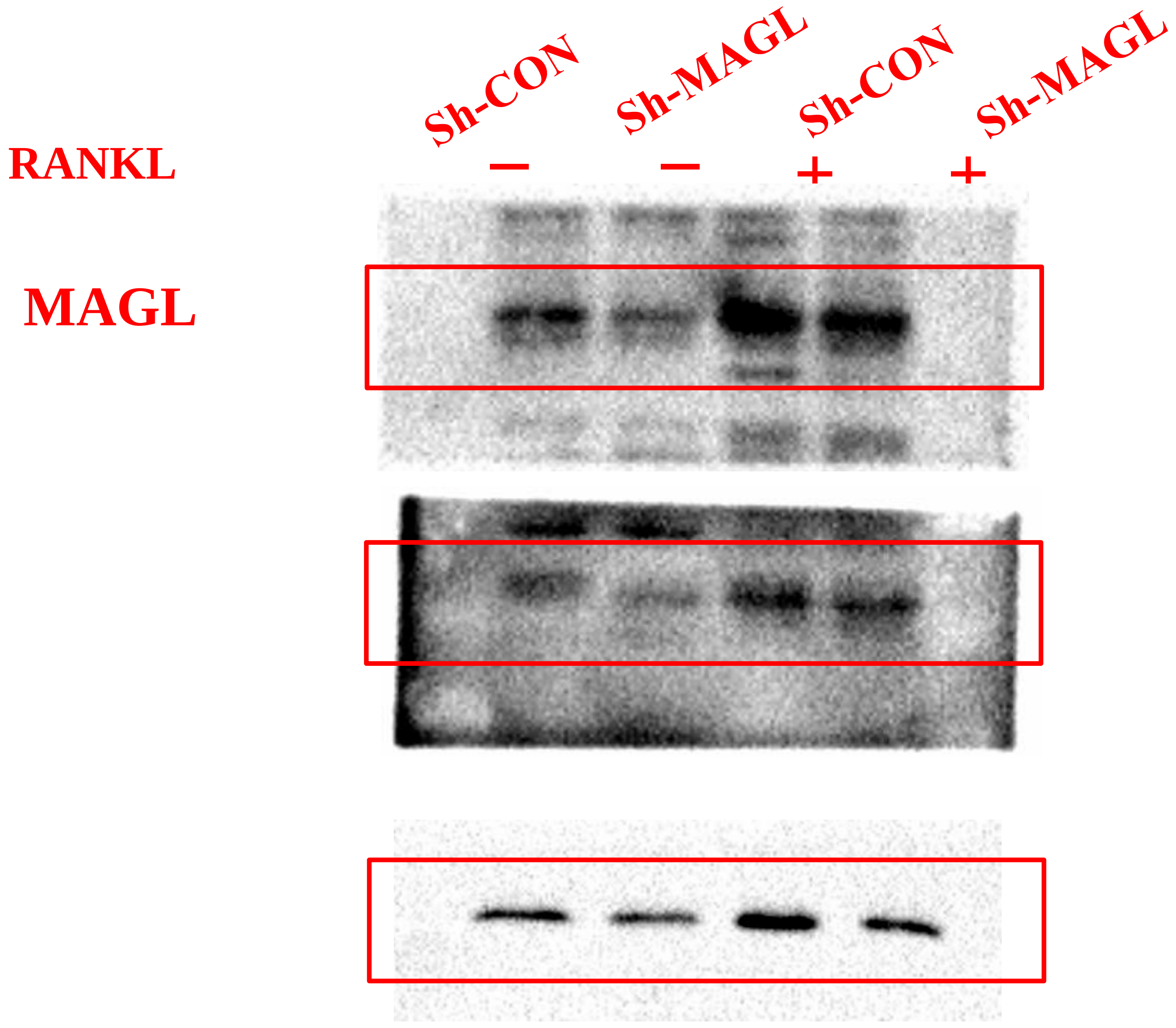
Sh-CON
Sh-MAGL
Sh-CON
Sh-MAGL
RANKL
−
−
+
+
MAGL

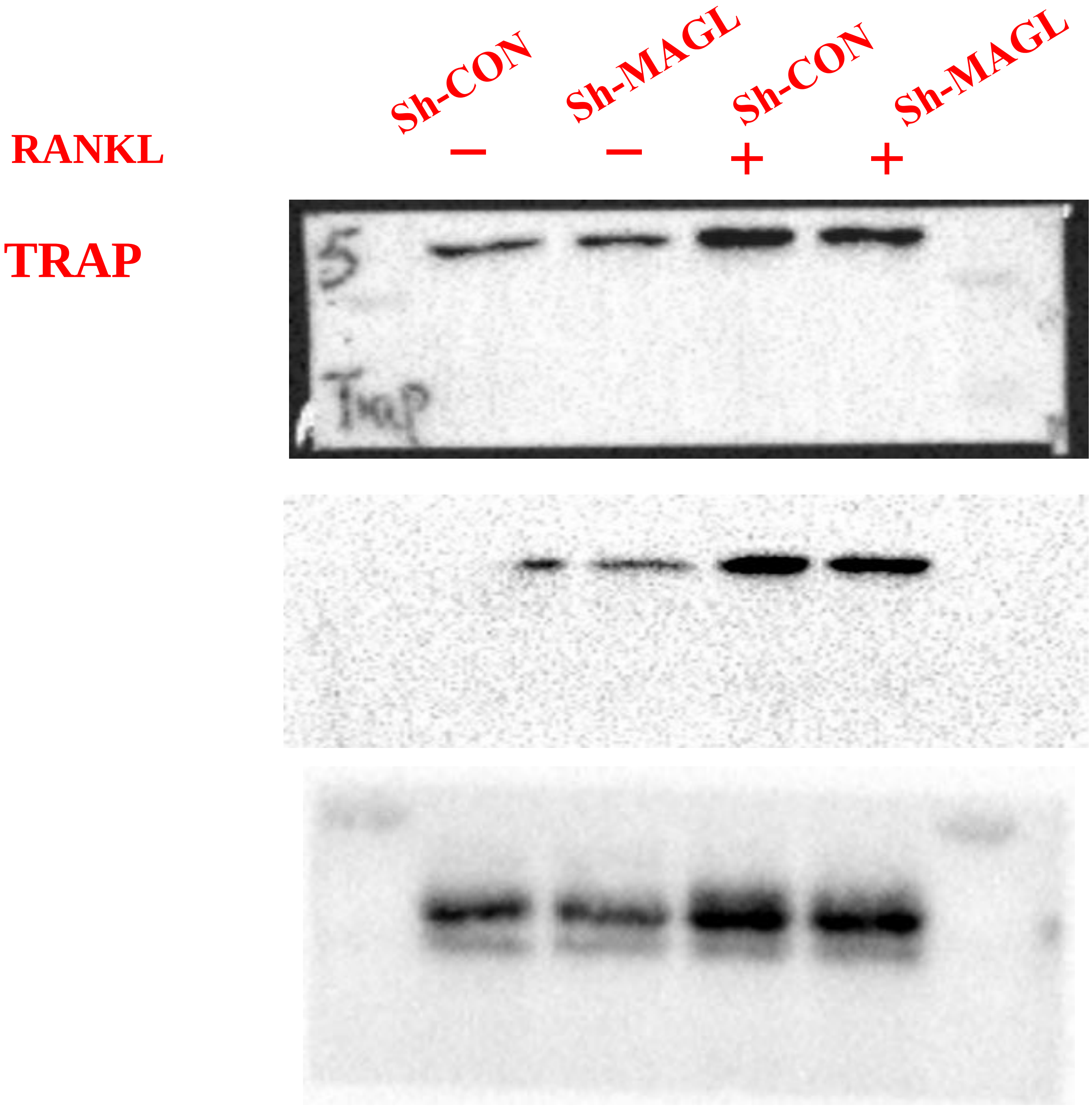
Sh-CON
Sh-MAGL
Sh-CON
Sh-MAGL
RANKL
−
−
+
+
TRAP

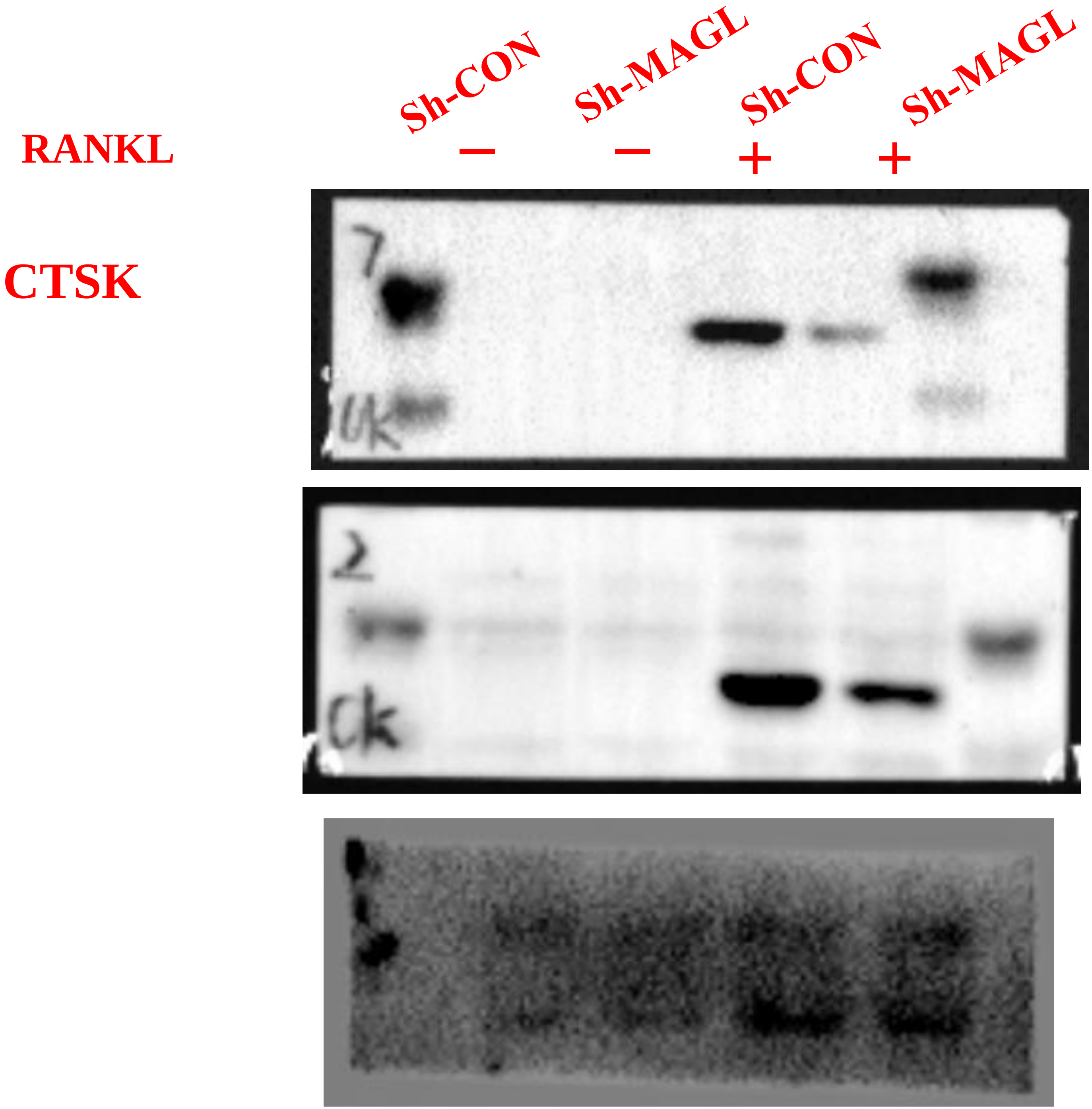

Sh-CON
Sh-MAGL
Sh-CON
Sh-MAGL
RANKL
–
–
+
+
CTSK

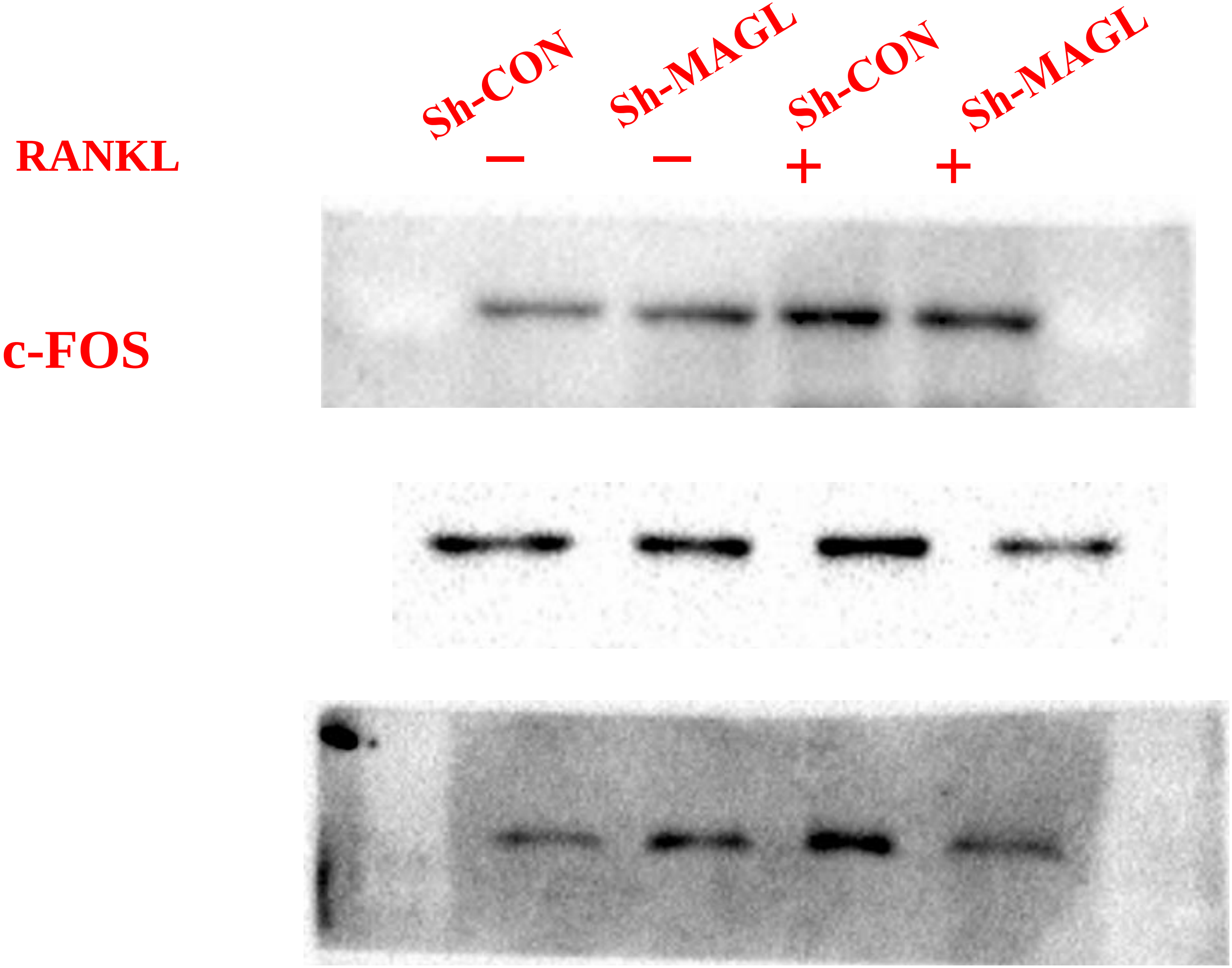
Sh-CON
Sh-MAGL
Sh-CON
Sh-MAGL
RANKL
−
−
+
+
c-FOS

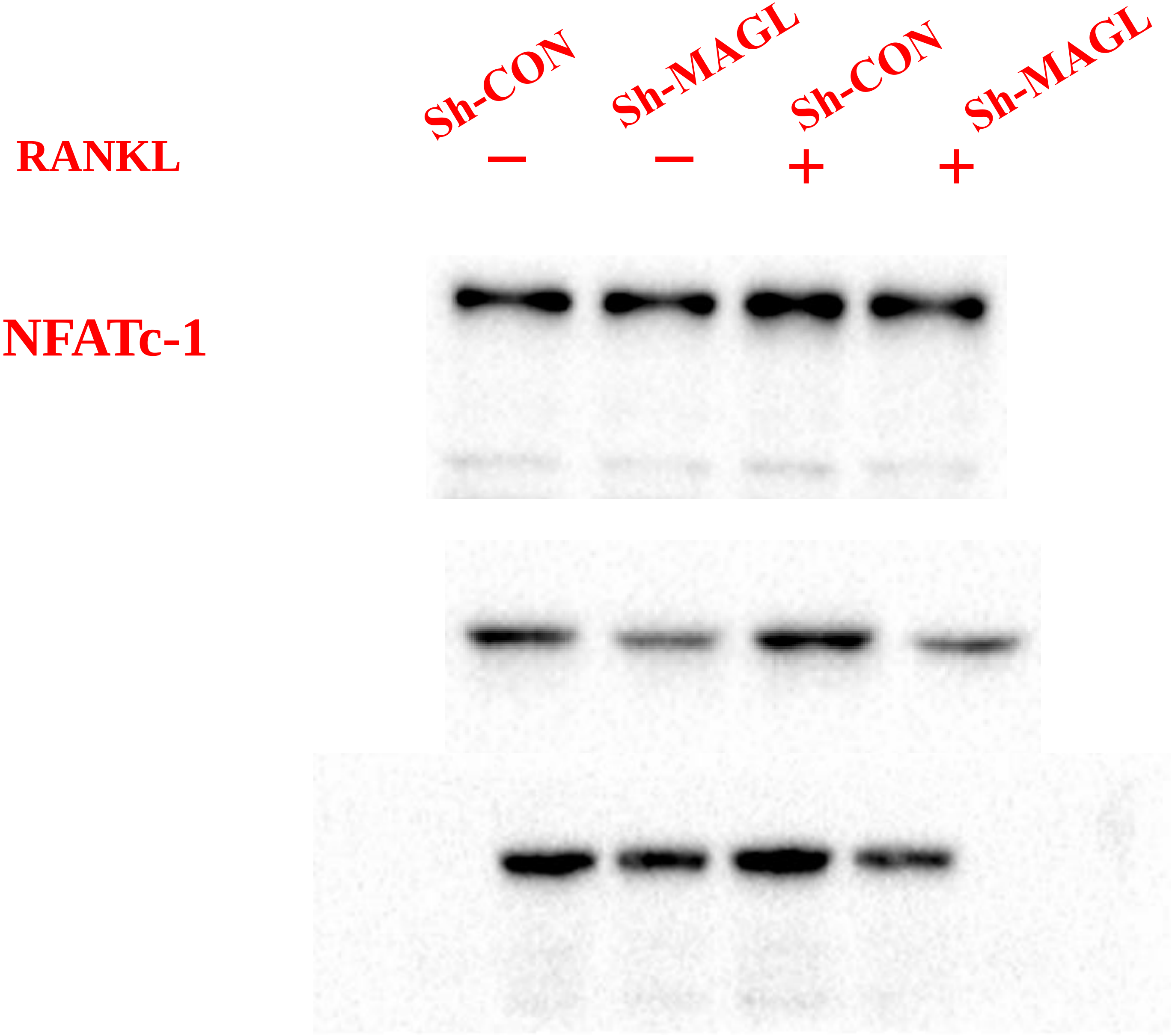
Sh-CON
Sh-MAGL
Sh-CON
Sh-MAGL
RANKL
−
−
+
+
NFATc-1

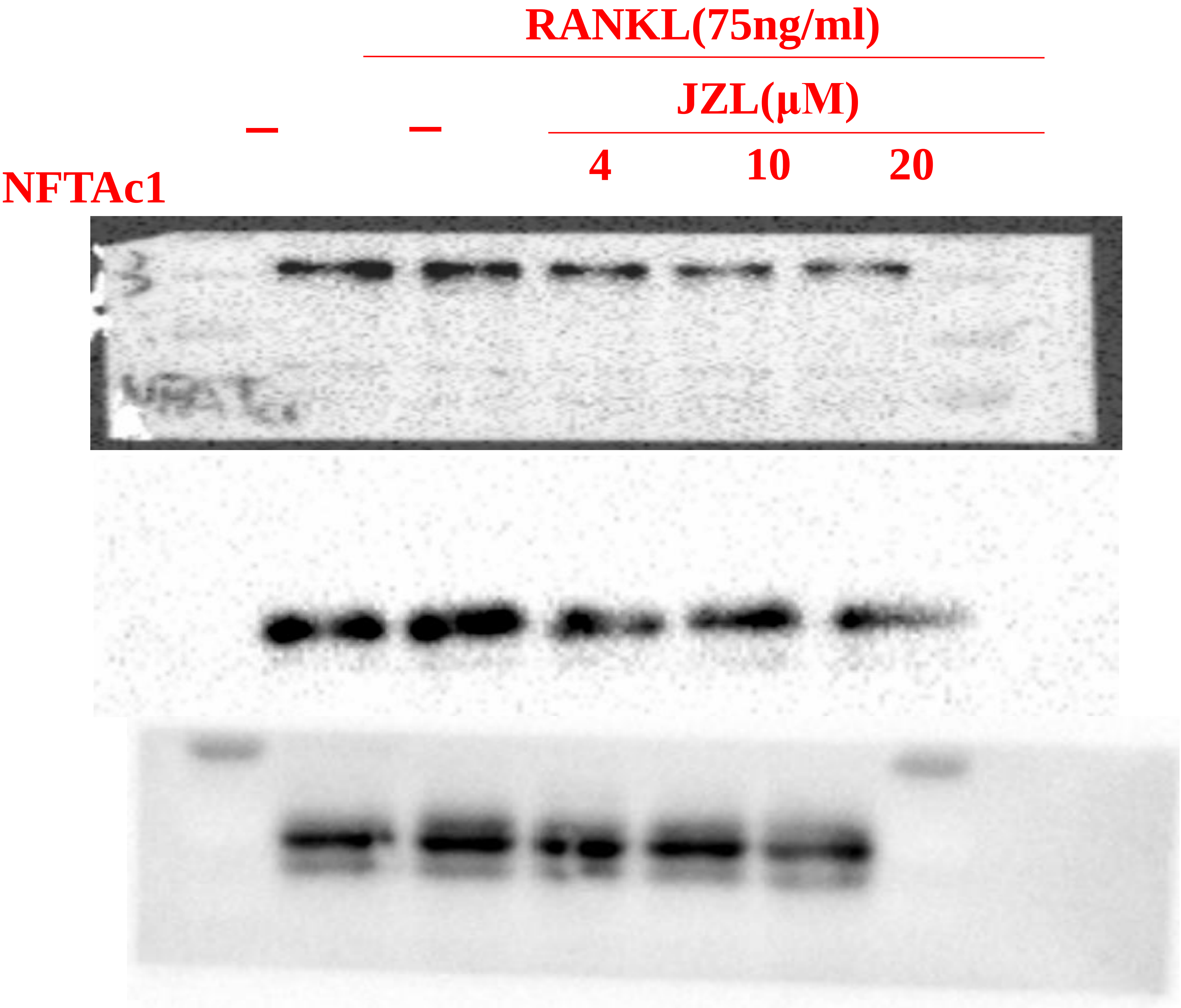

RANKL(75ng/ml)
JZL(μM)
–
–
4
10
20
NFTAc1

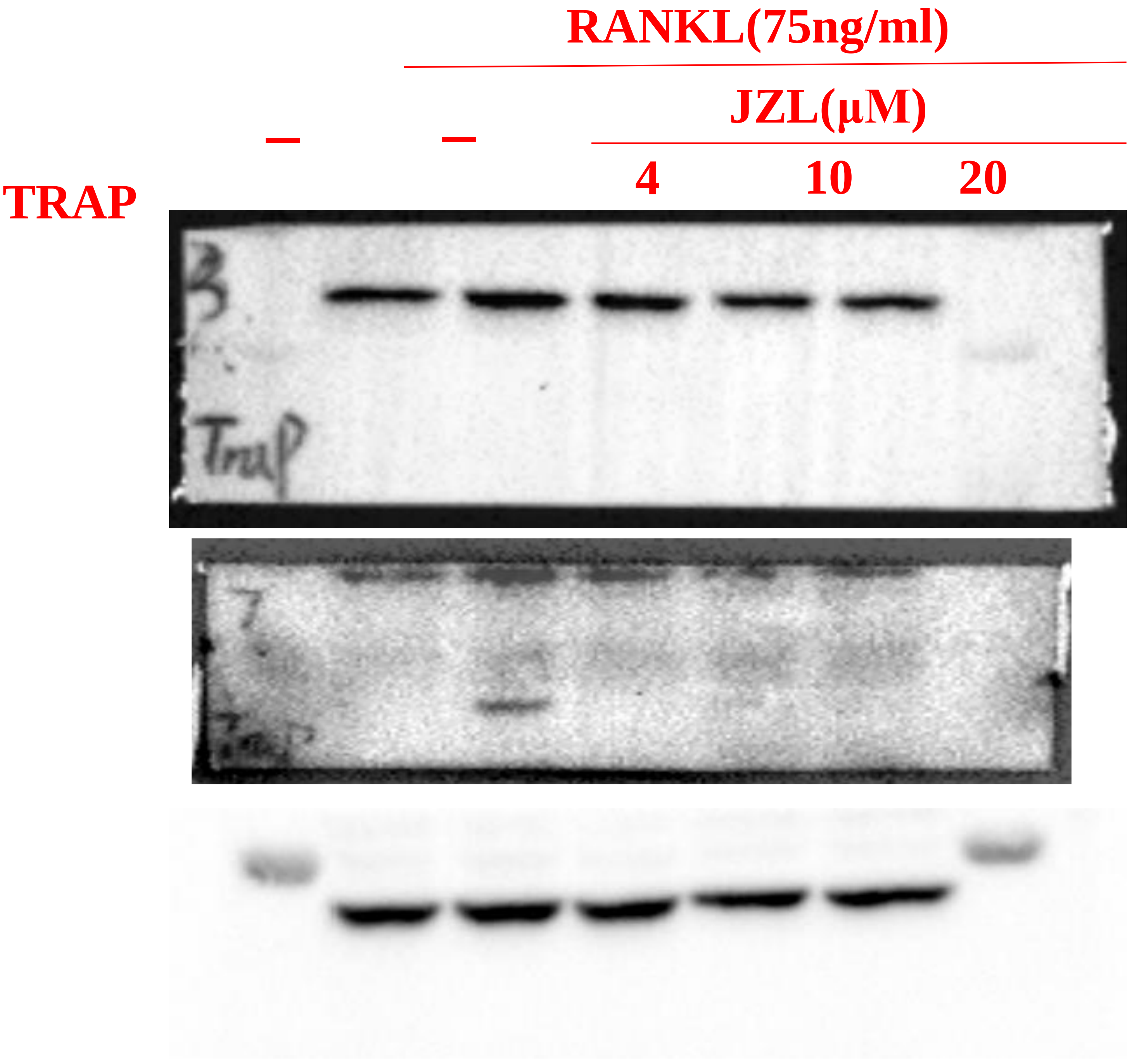
RANKL(75ng/ml)
JZL(μM)
–
–
4
10
20
TRAP

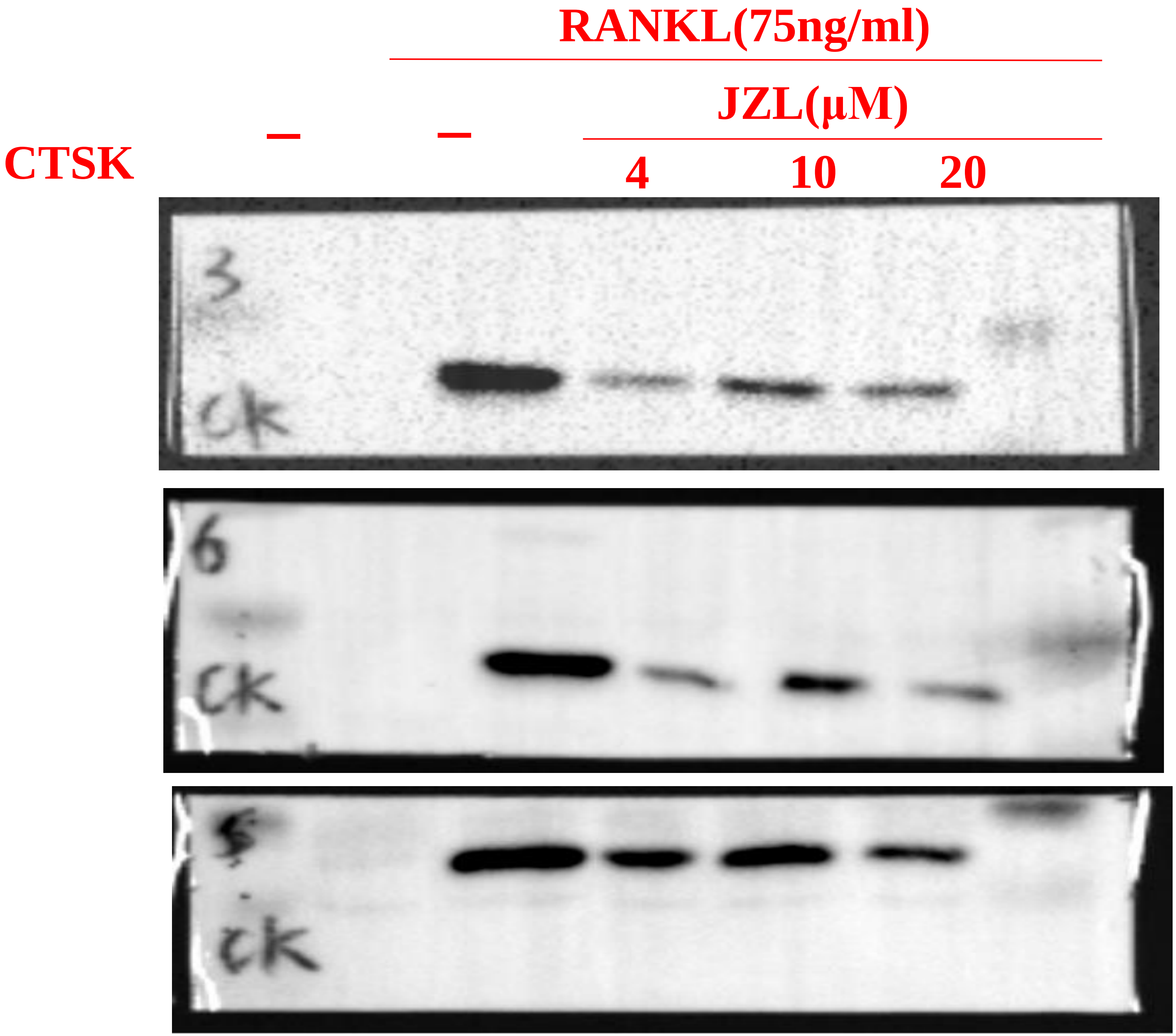
RANKL(75ng/ml)
JZL(μM)
CTSK
–
–
4
10
20

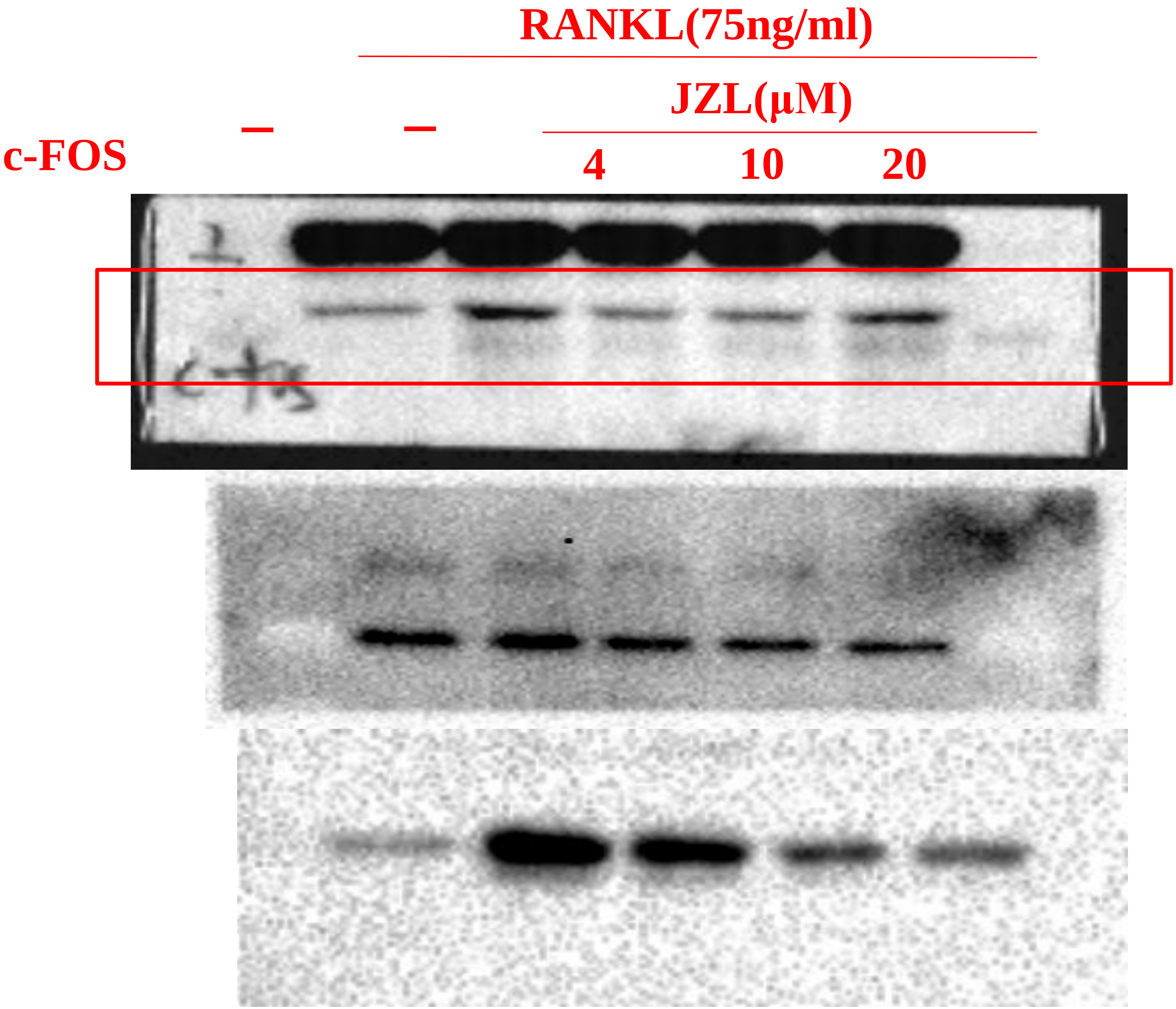
RANKL(75ng/ml)
JZL(μM)
c-FOS
–
–
4
10
20

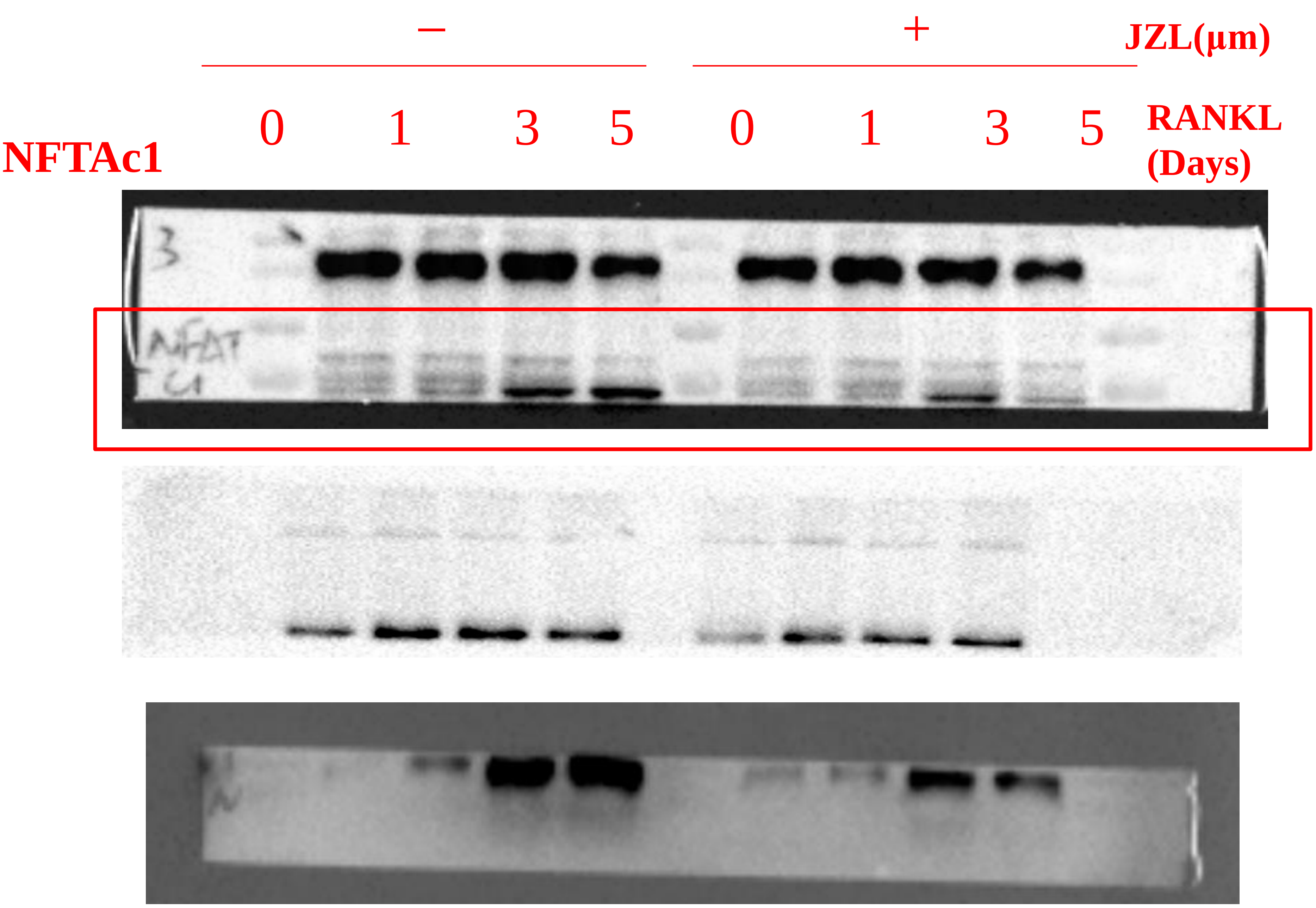

–
+
JZL(μm)
0
1
3
5
0
1
3
5
RANKL
(Days)
NFTAc1

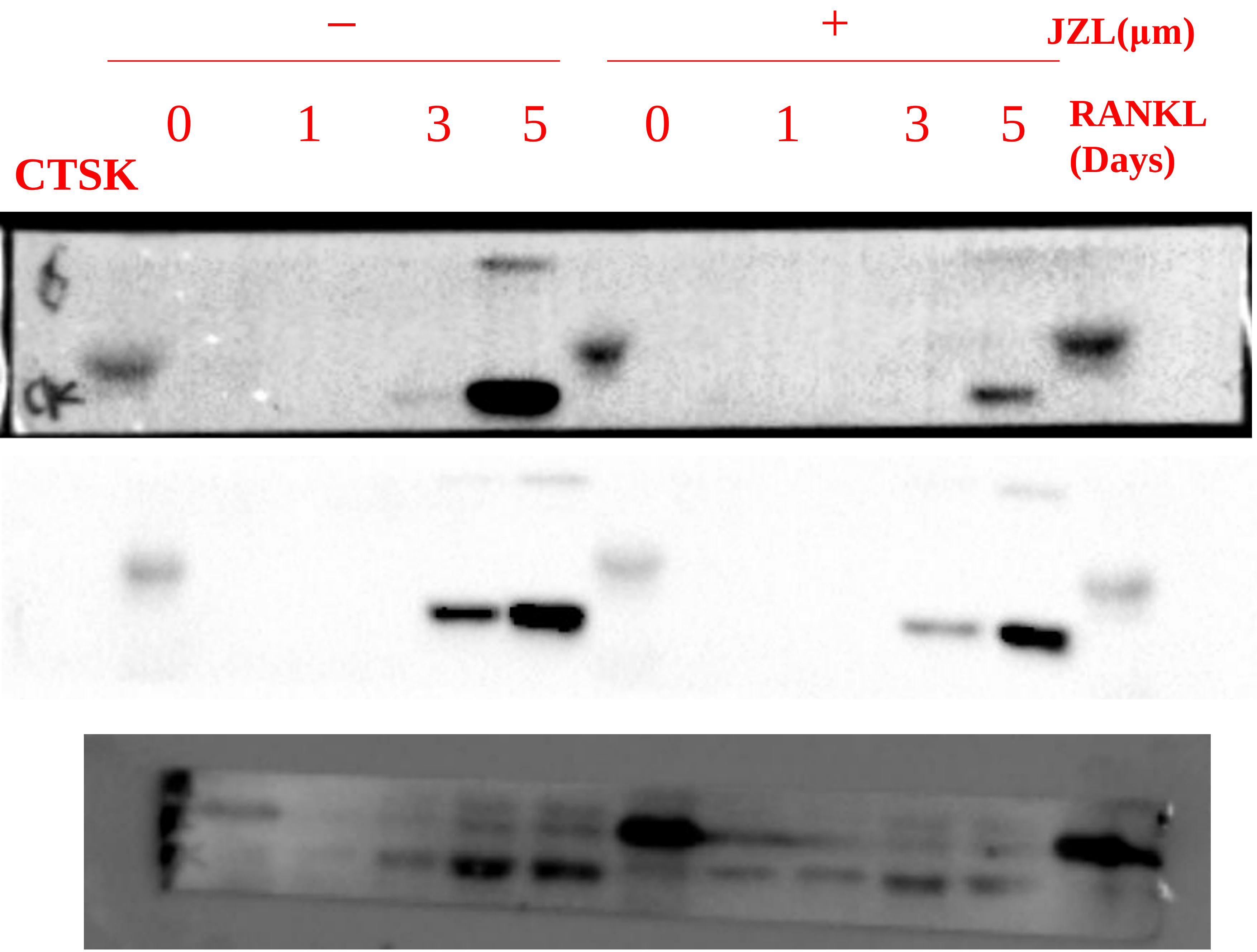

–
+
JZL(μm)
0
1
3
5
0
1
3
5
RANKL
(Days)
CTSK

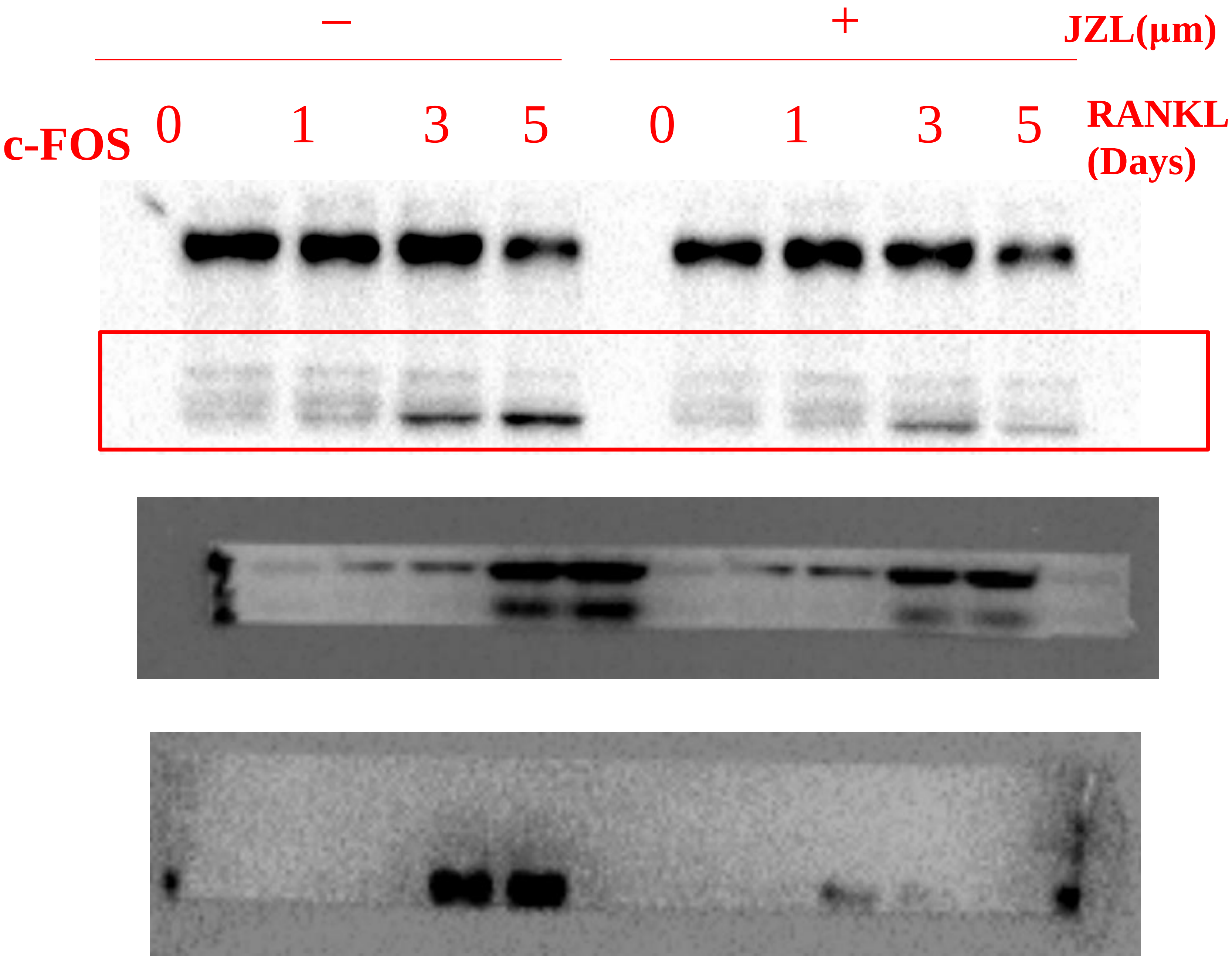

–
+
JZL(μm)
c-FOS
0
1
3
5
0
1
3
5
RANKL
(Days)

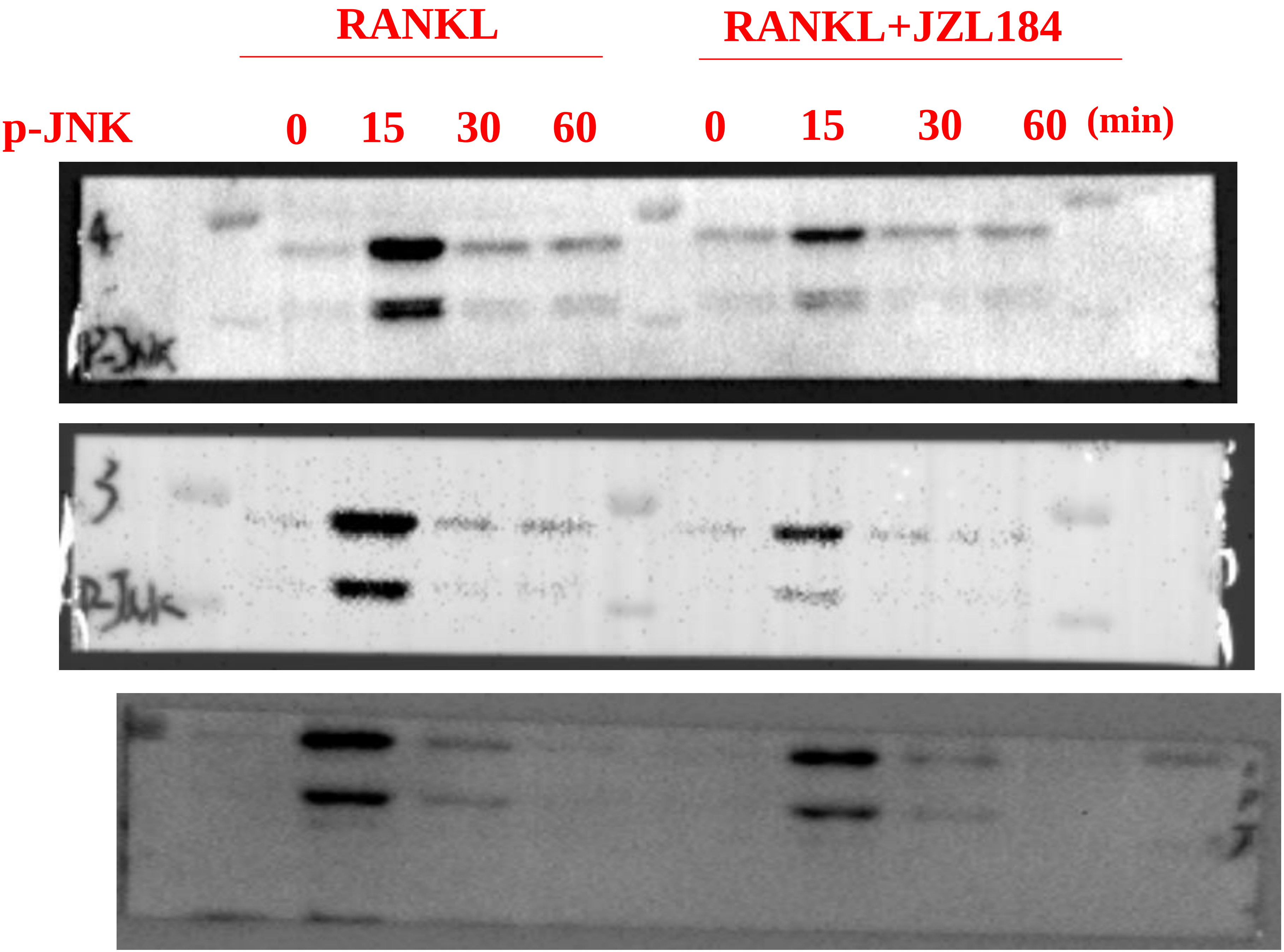
RANKL
RANKL+JZL184
p-JNK
0 15 30 60
0 15 30 60 (min)

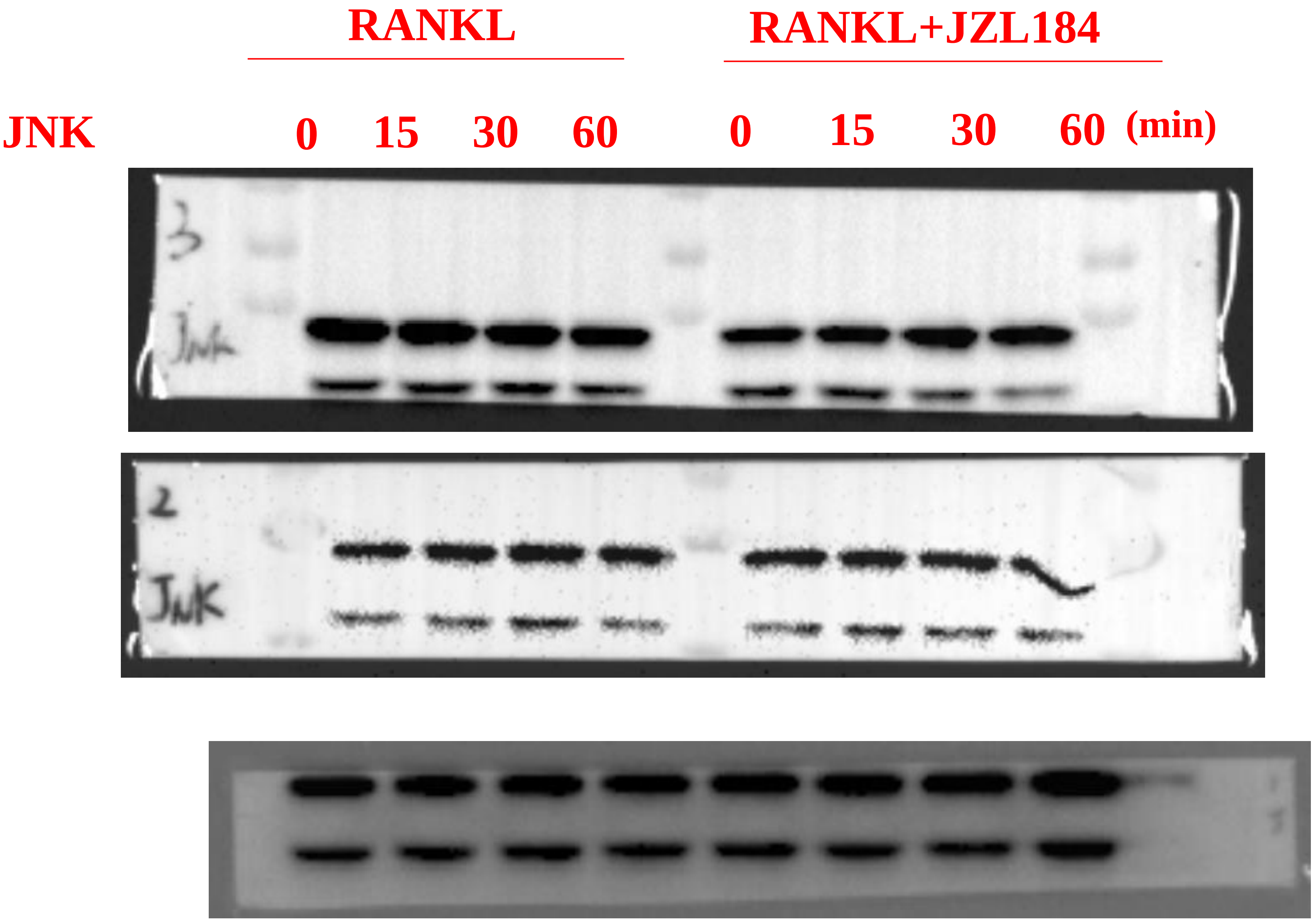
RANKL
RANKL+JZL184
JNK
0 15 30 60
0 15 30 60 (min)

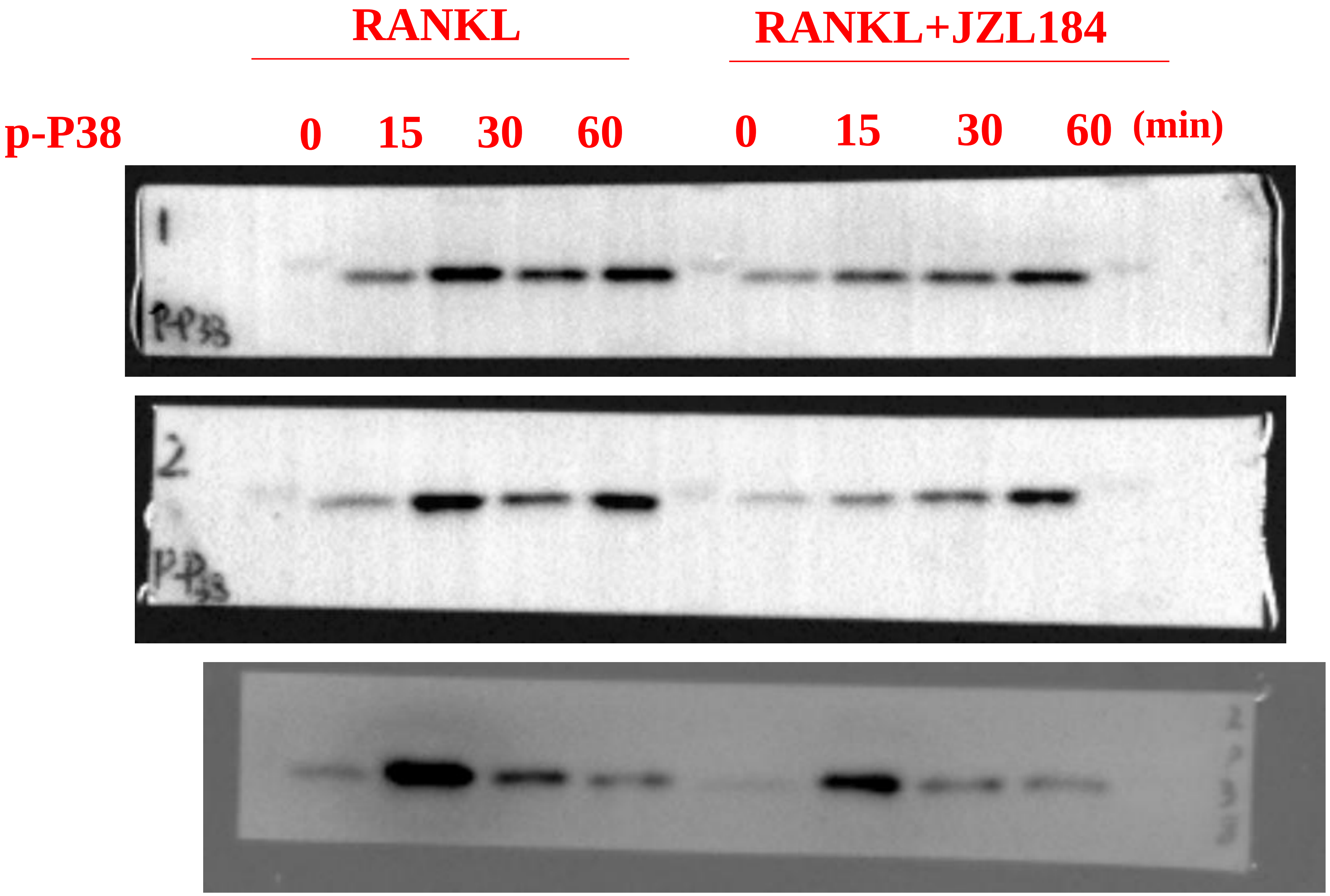
RANKL
RANKL+JZL184
p-P38
0
15
30
60
0
15
30
60
(min)

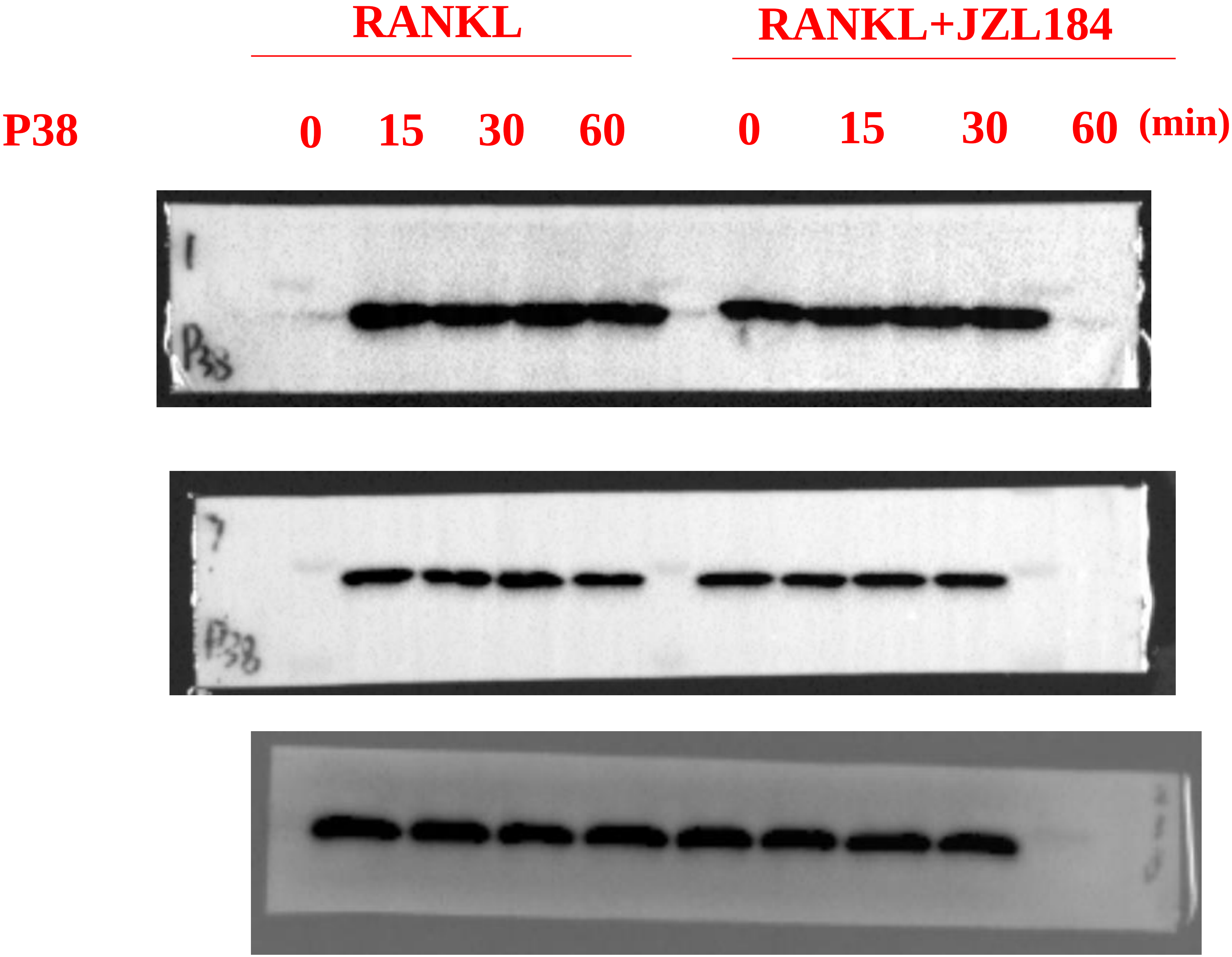
RANKL
RANKL+JZL184
P38
0 15 30 60
0 15 30 60 (min)

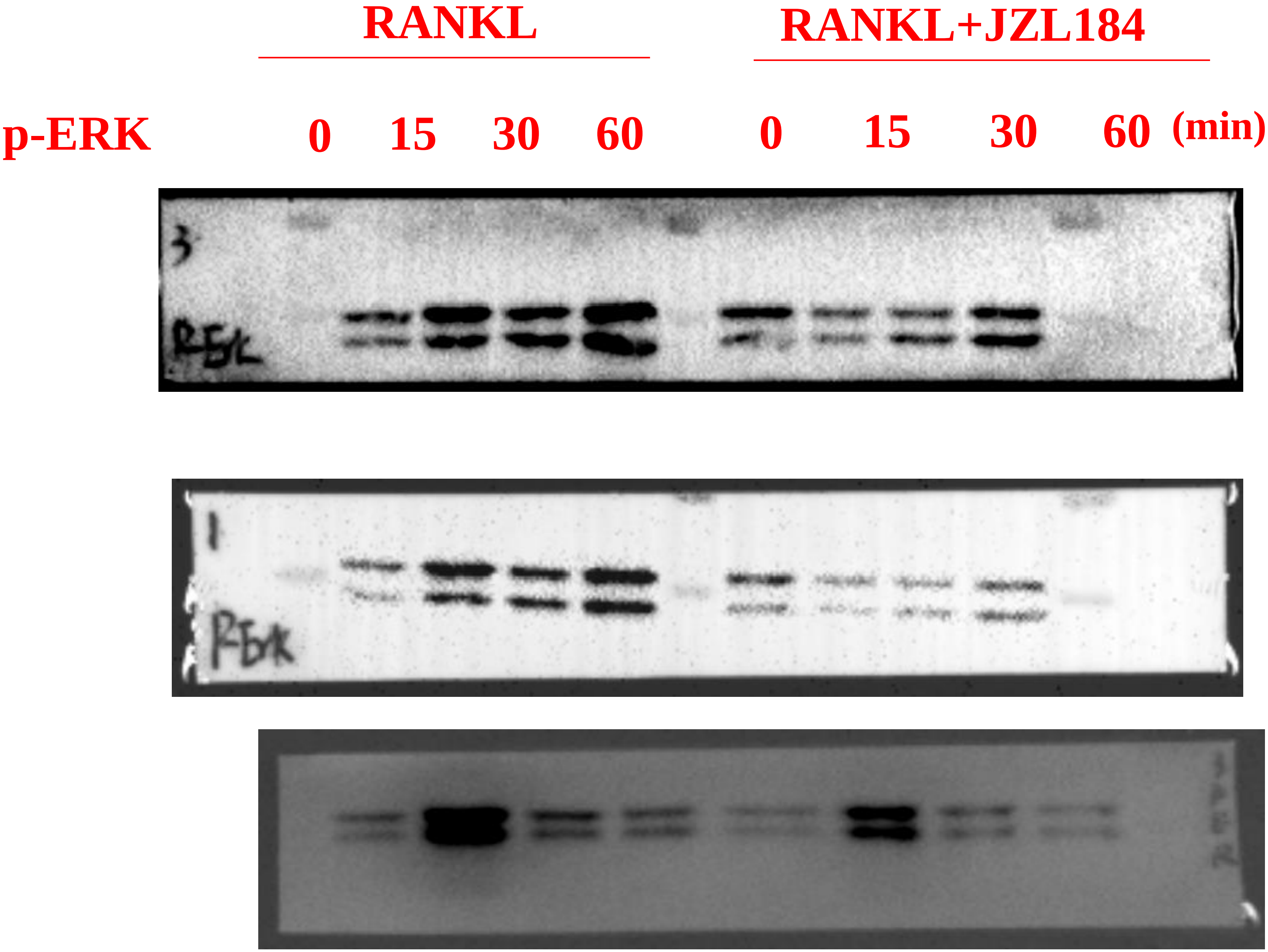
RANKL
RANKL+JZL184
p-ERK
0 15 30 60
0 15 30 60 (min)

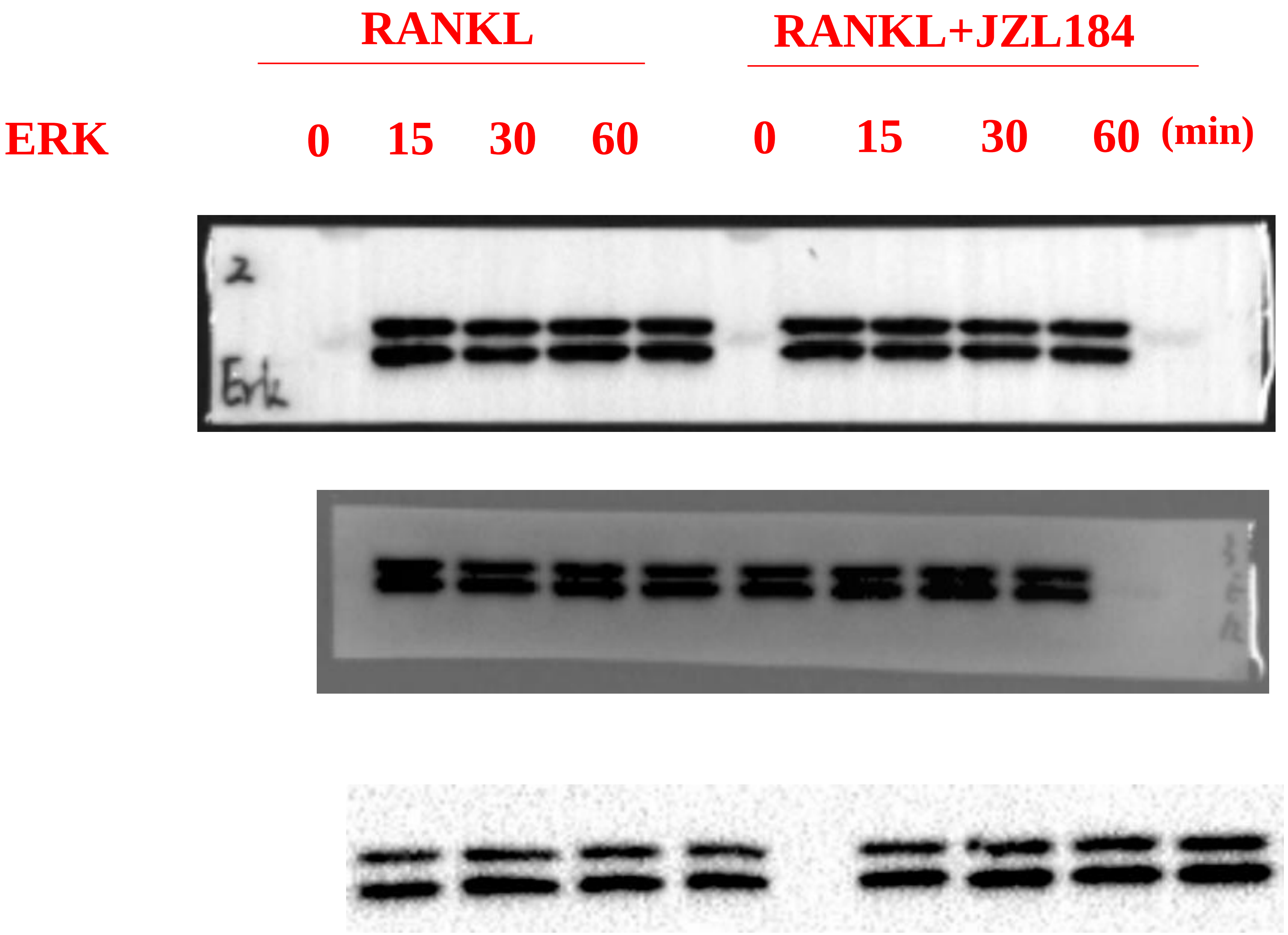
RANKL
RANKL+JZL184
ERK
0 15 30 60
0 15 30 60 (min)
2
Erk

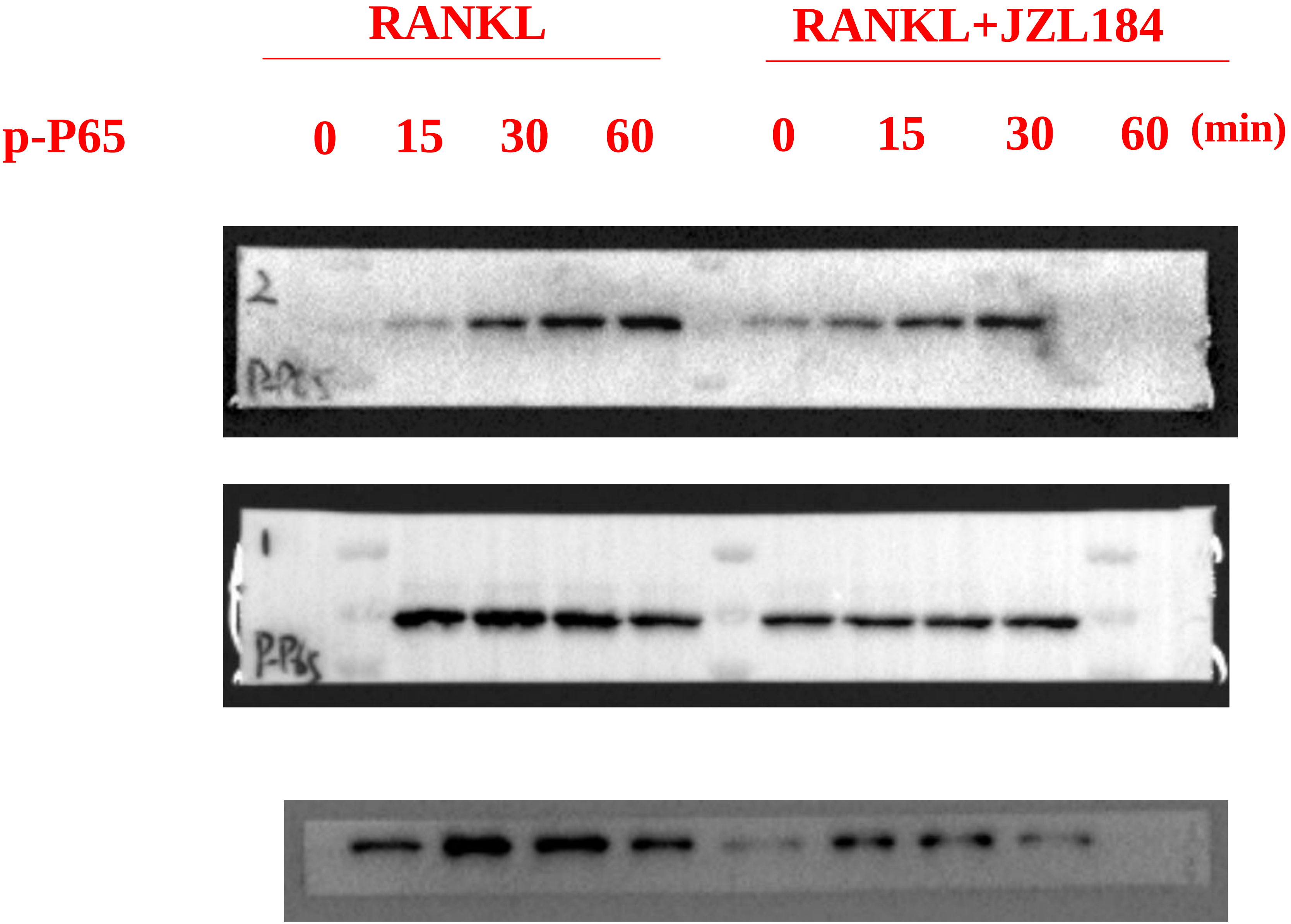
RANKL
RANKL+JZL184
p-P65
0
15
30
60
0
15
30
60
(min)

RANKL | RANKL+JZL184

P65 | 0 15 30 60 | 0 15 30 60 (min)

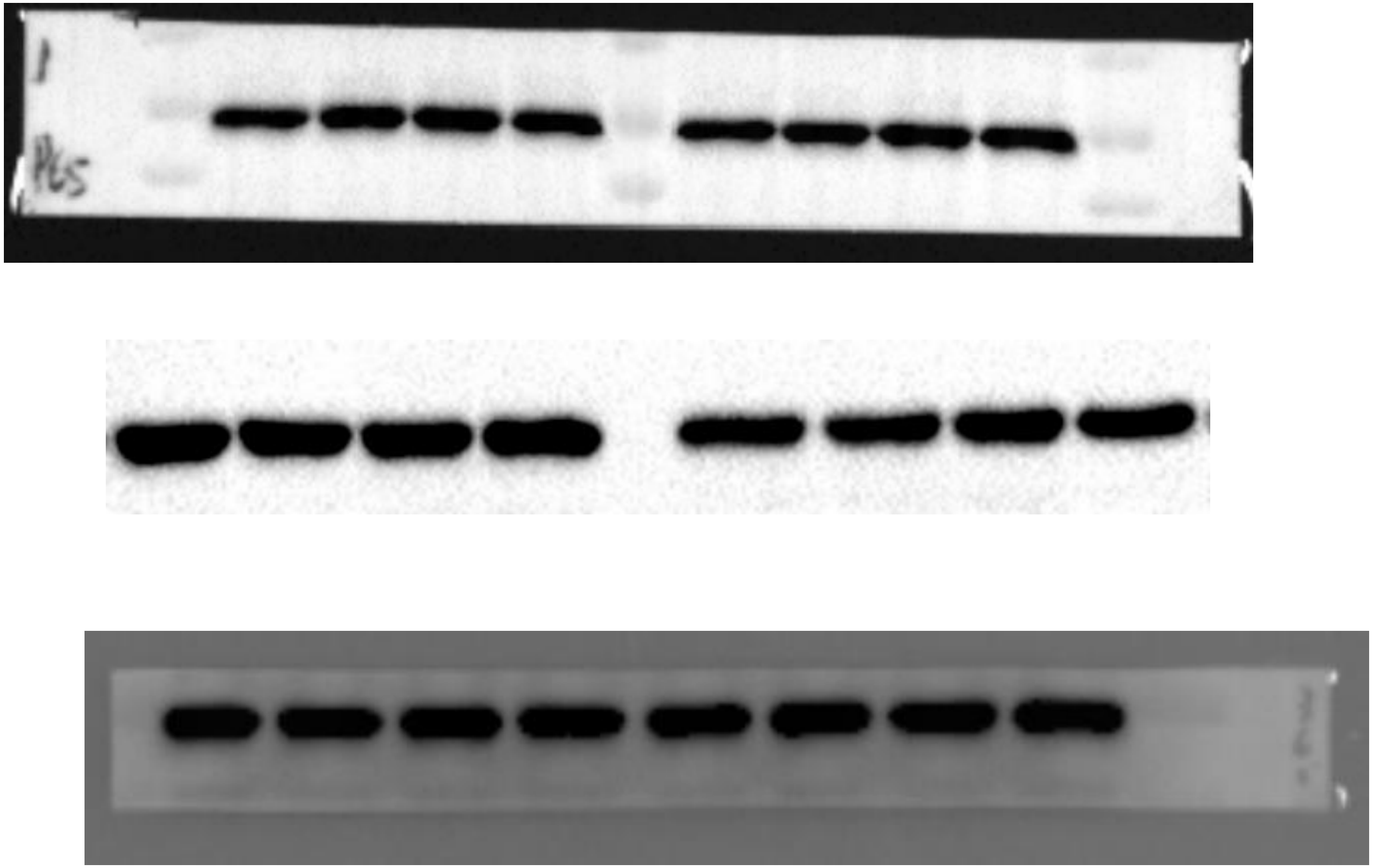

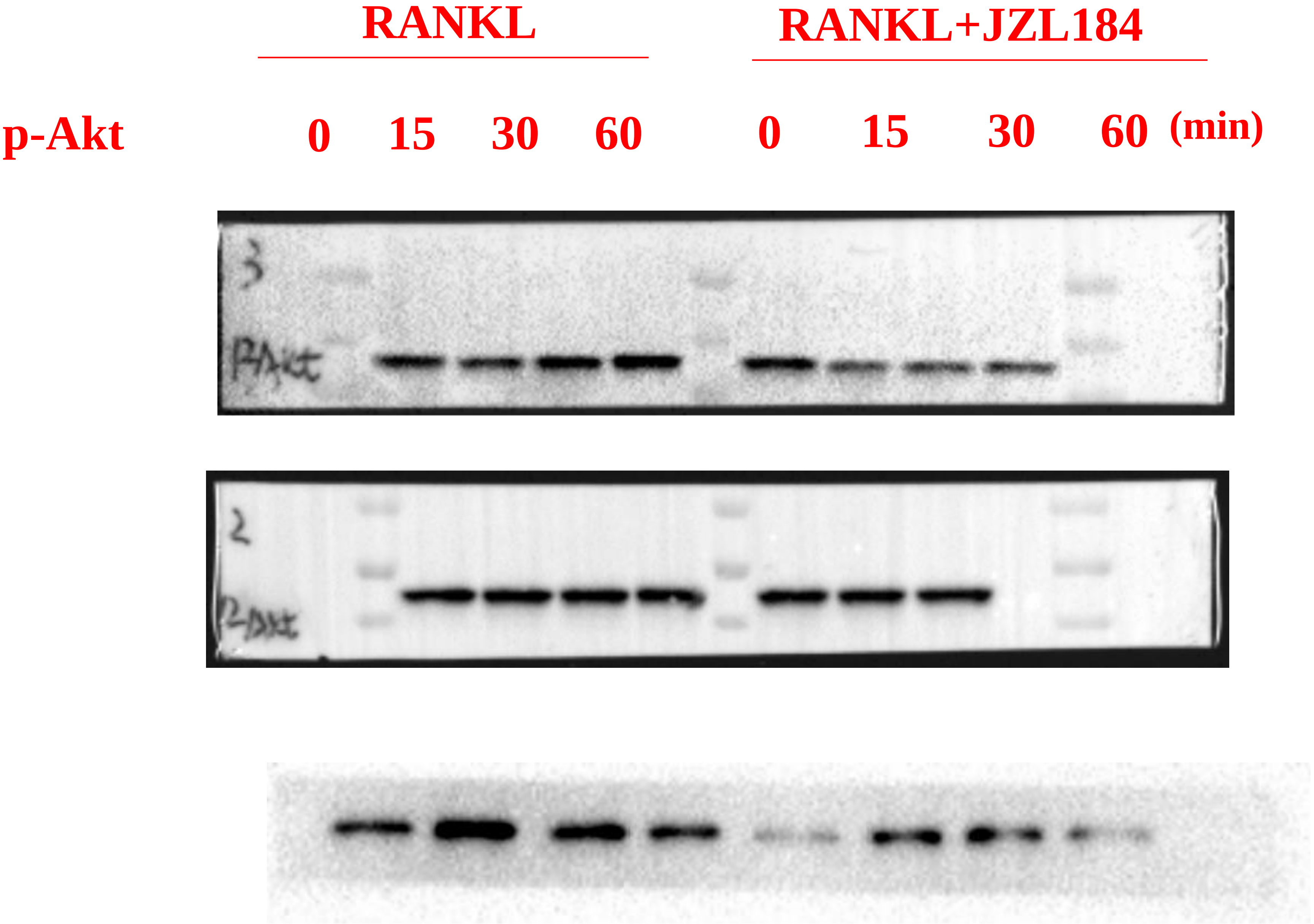
RANKL
RANKL+JZL184
p-Akt
0
15
30
60
0
15
30
60
(min)

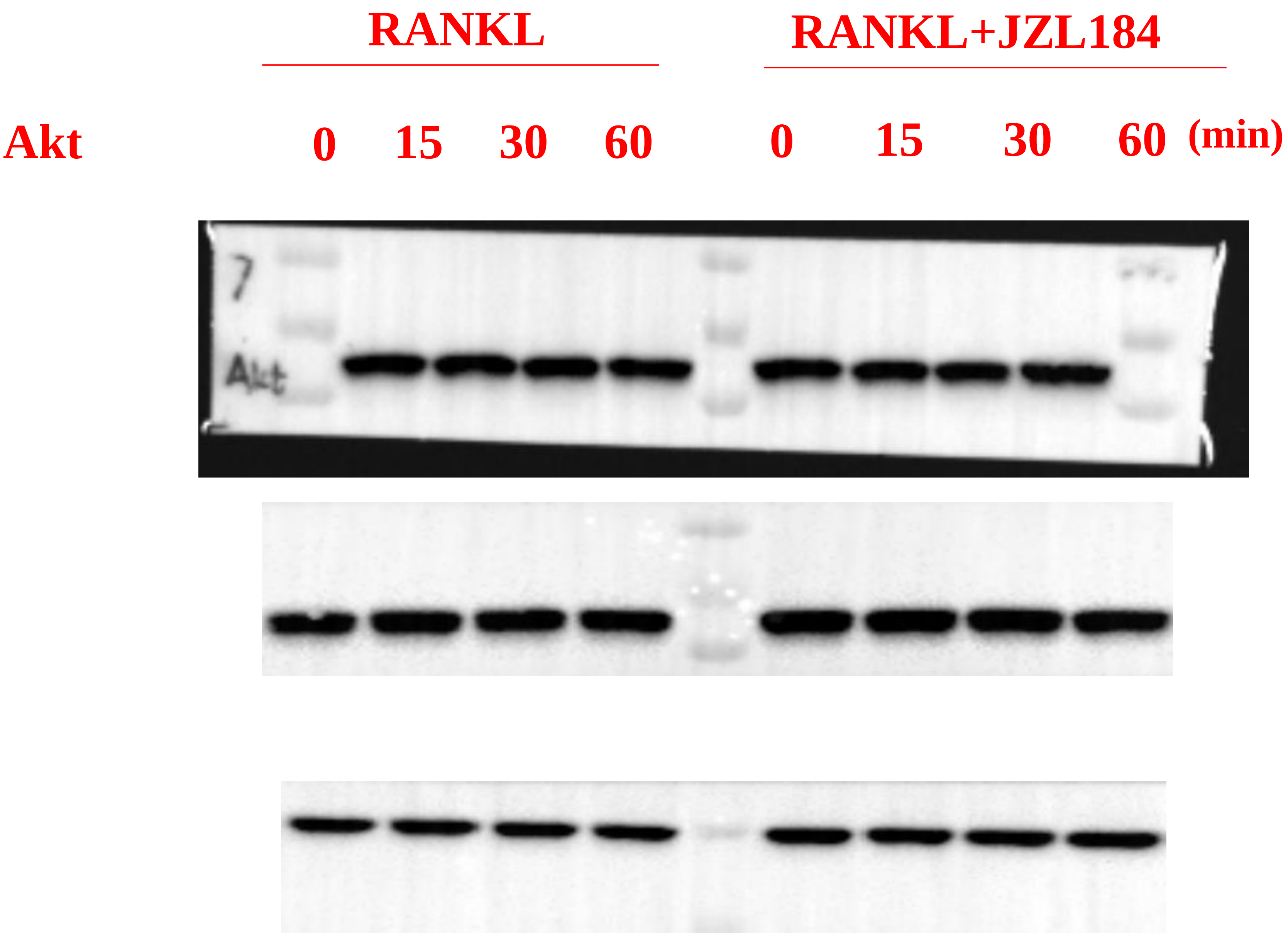
RANKL
RANKL+JZL184
Akt
0 15 30 60
0 15 30 60 (min)

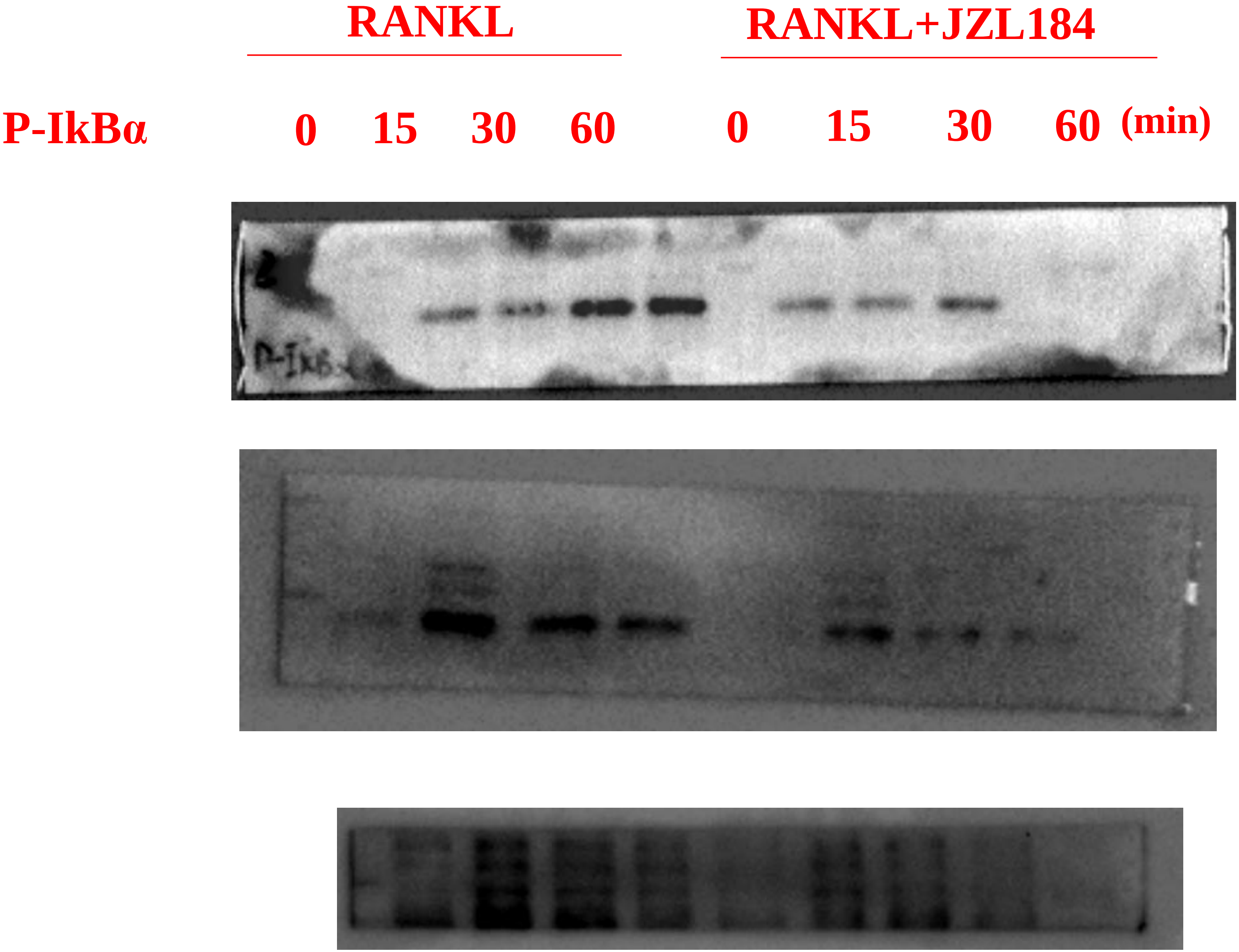
RANKL
RANKL+JZL184
P-IkBα
0
15
30
60
0
15
30
60
(min)

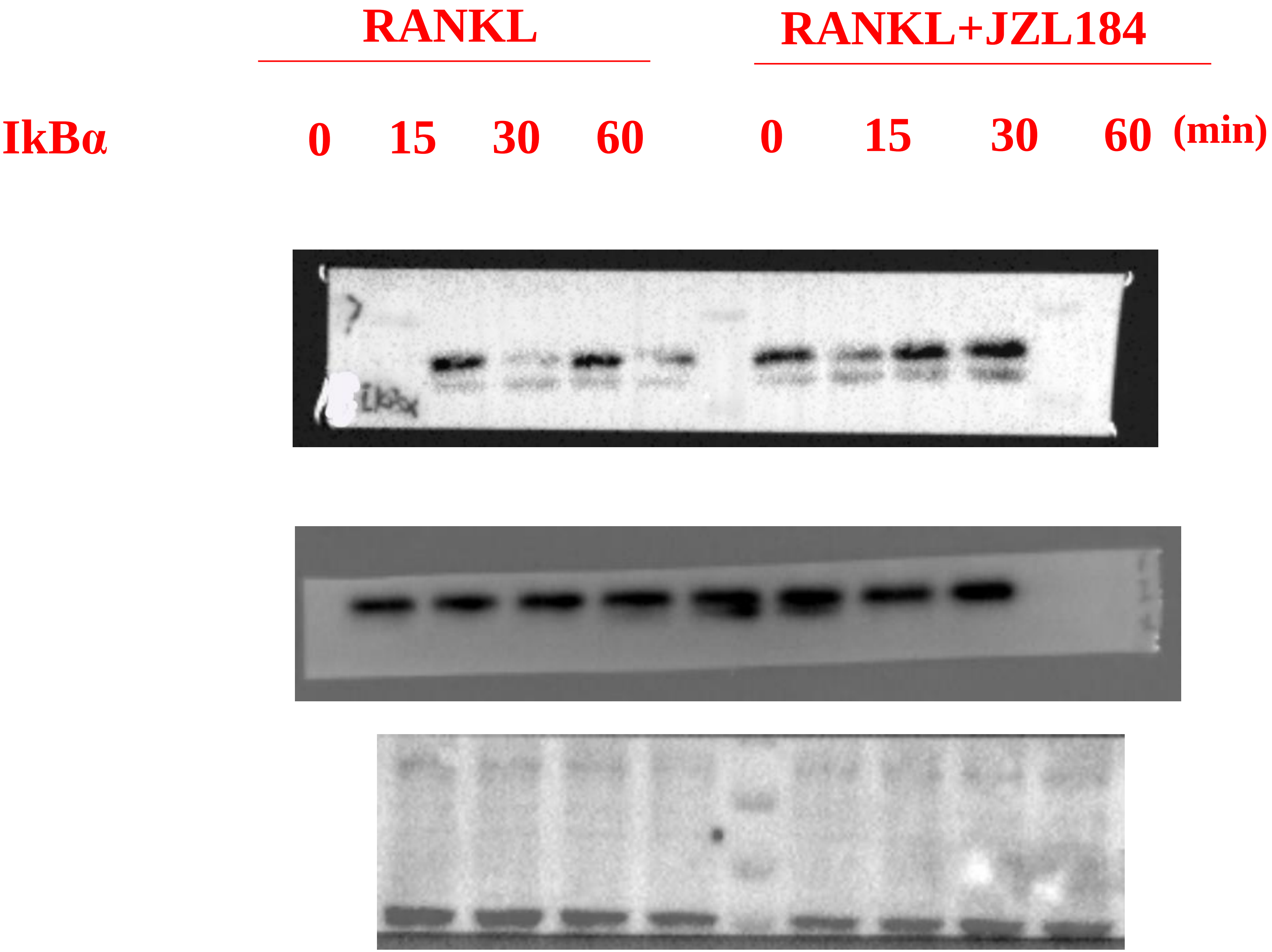
RANKL
RANKL+JZL184
IkBα
0 15 30 60
0 15 30 60 (min)

RANKL RANKL+JZL184

P-IKKβ 0 15 30 60 0 15 30 60 (min)

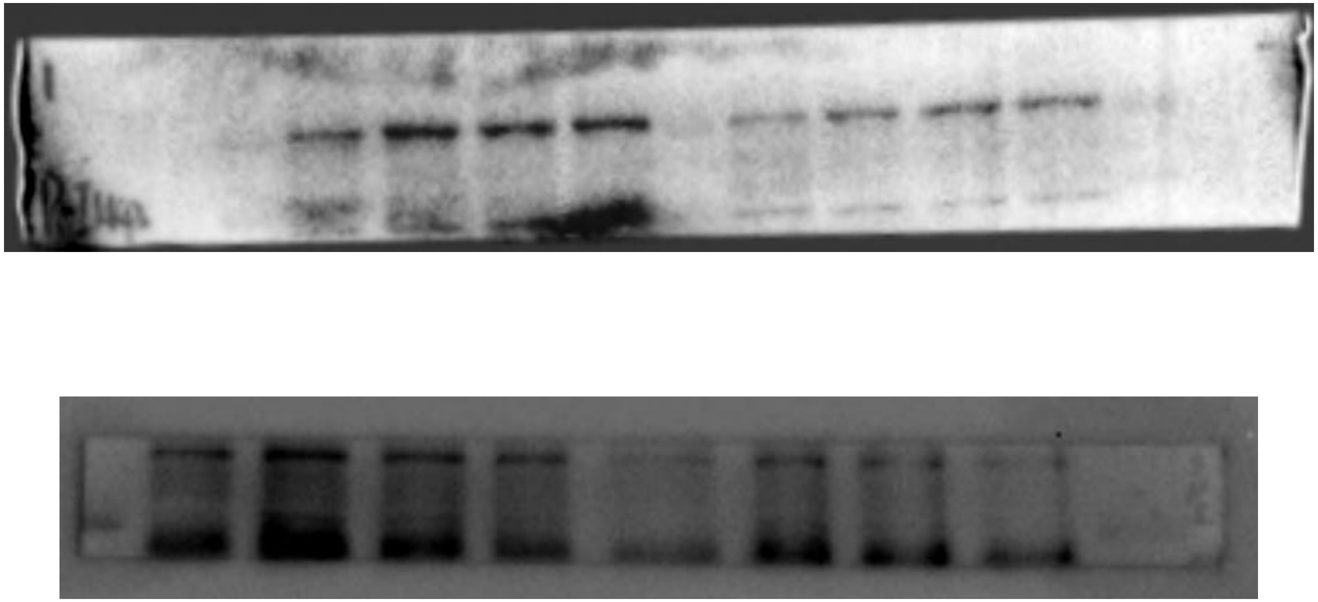

RANKL | RANKL+JZL184

IKKβ 0 15 30 60 0 15 30 60 (min)

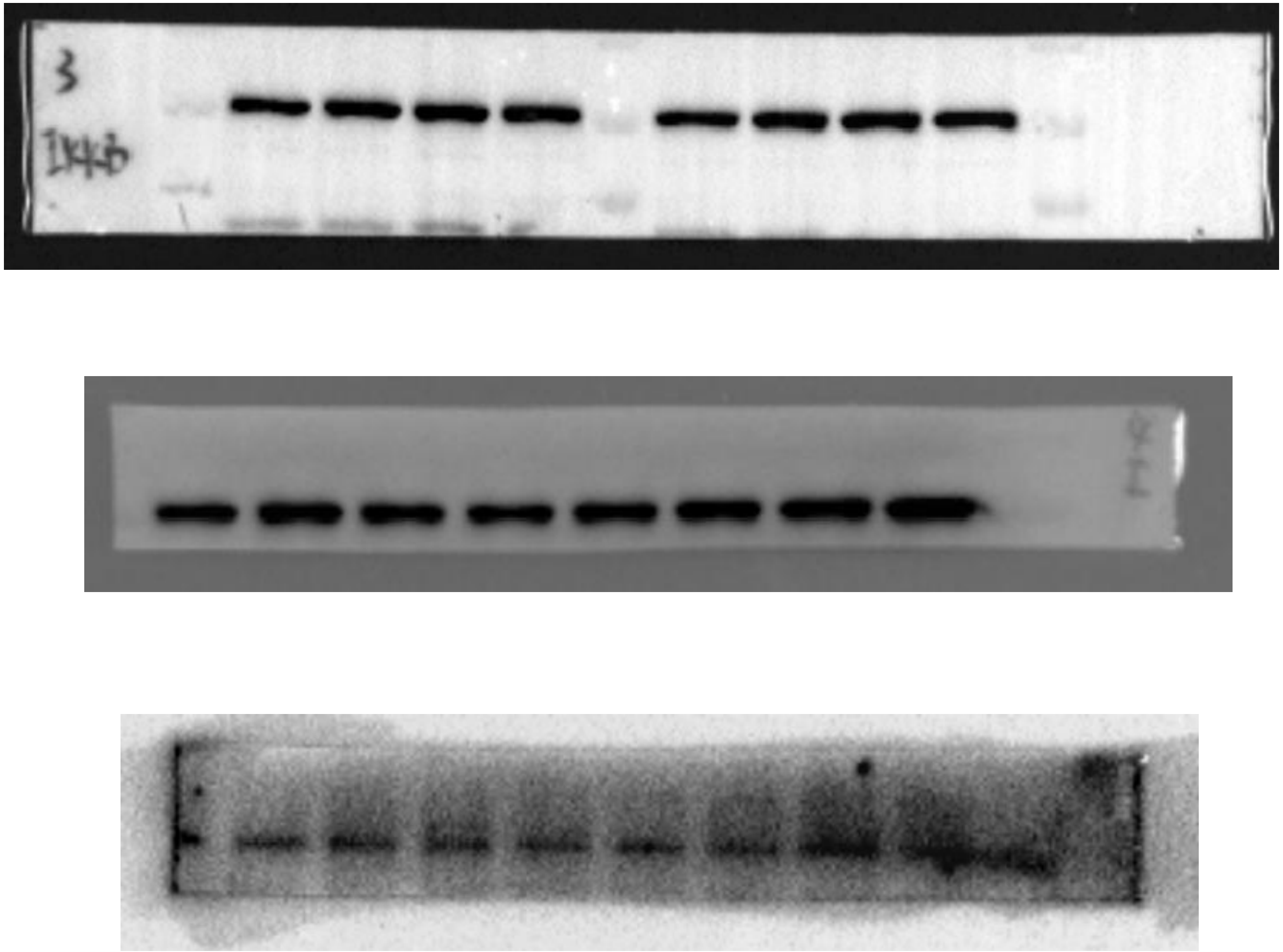

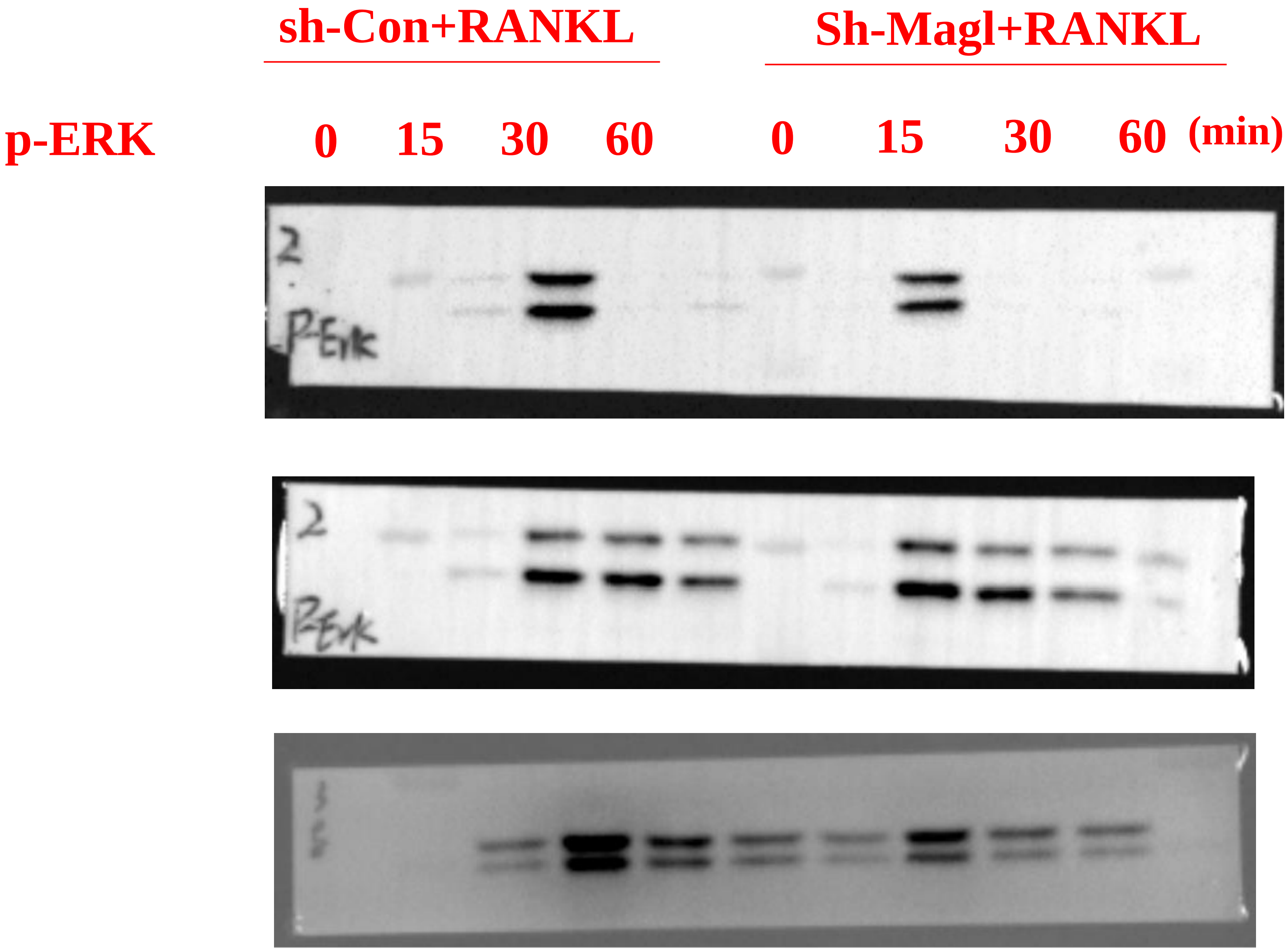
sh-Con+RANKL
Sh-Magl+RANKL
p-ERK
0 15 30 60 0 15 30 60 (min)

sh-Con+RANKL | Sh-Magl+RANKL

ERK 0 15 30 60 0 15 30 60 (min)

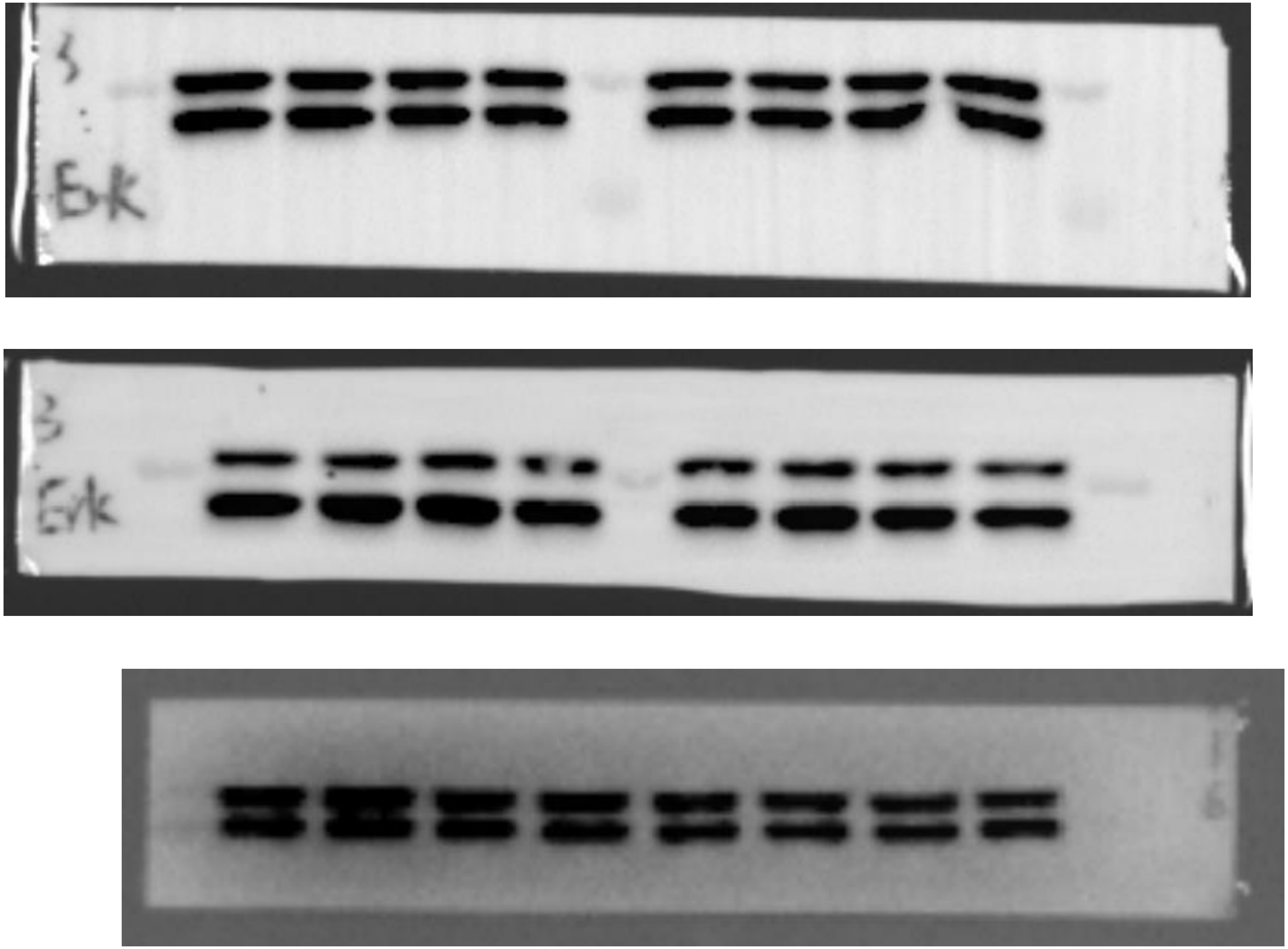

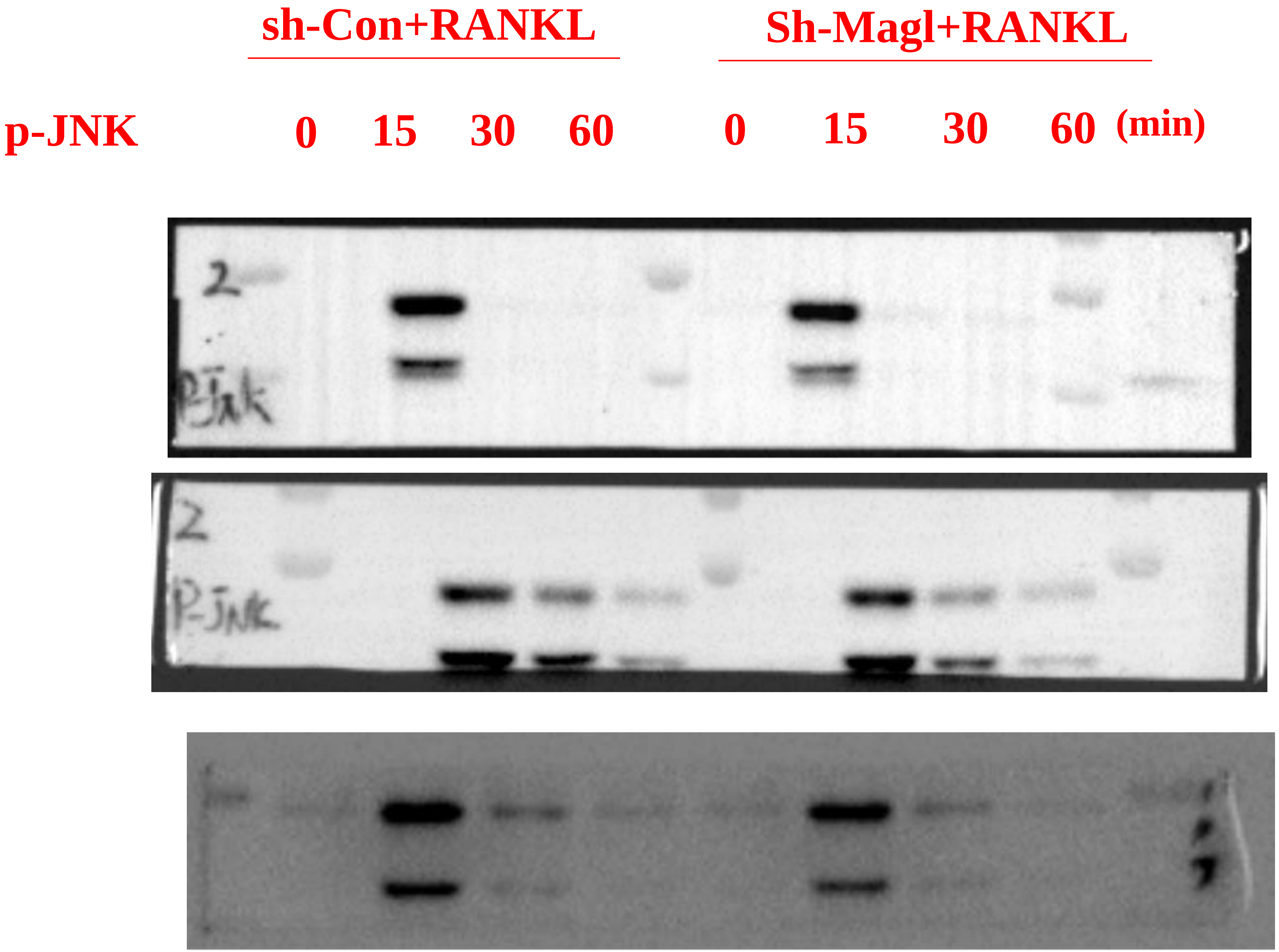
sh-Con+RANKL
Sh-Magl+RANKL
p-JNK
0
15
30
60
0
15
30
60
(min)

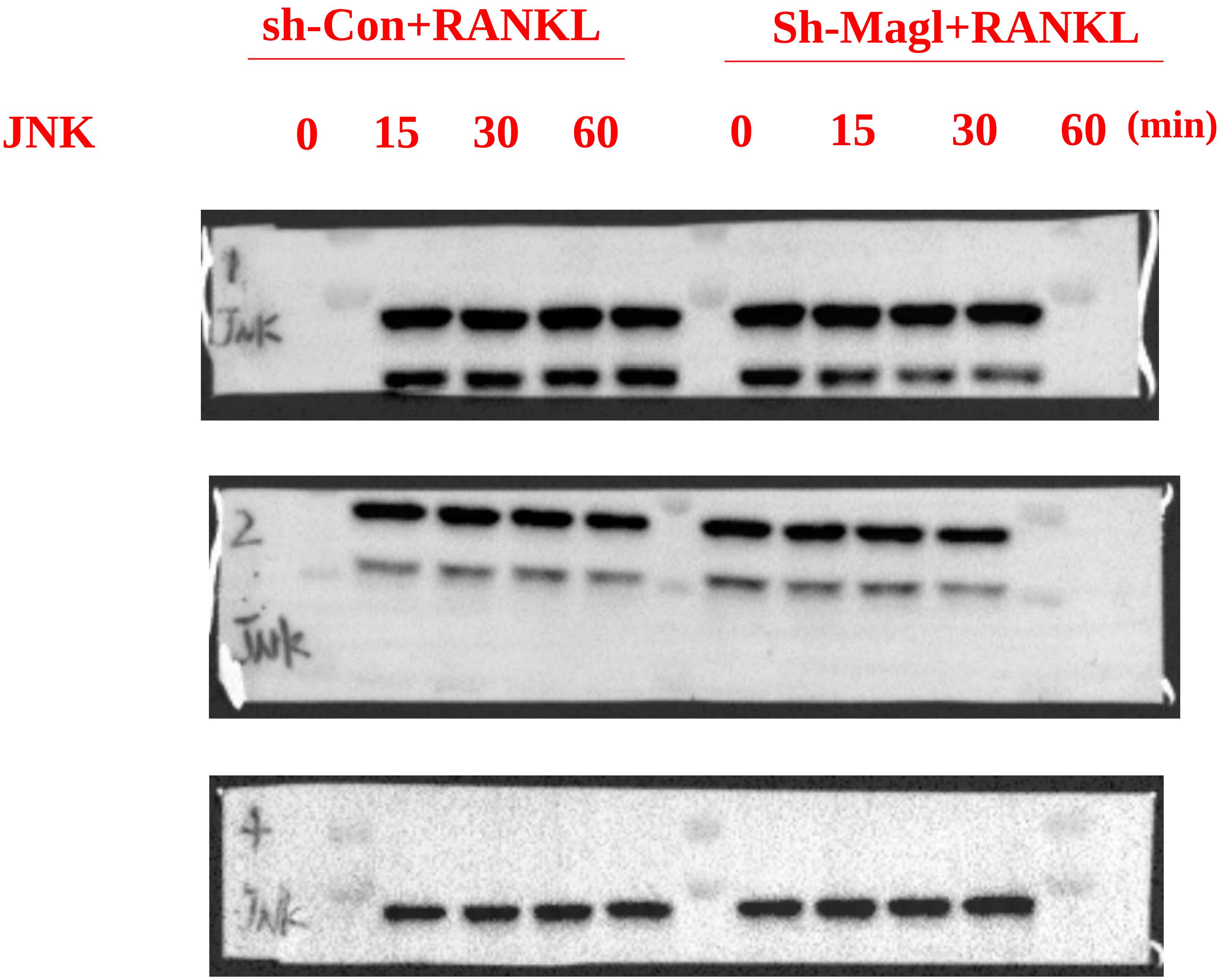
sh-Con+RANKL
Sh-Magl+RANKL
JNK
0 15 30 60
0 15 30 60 (min)

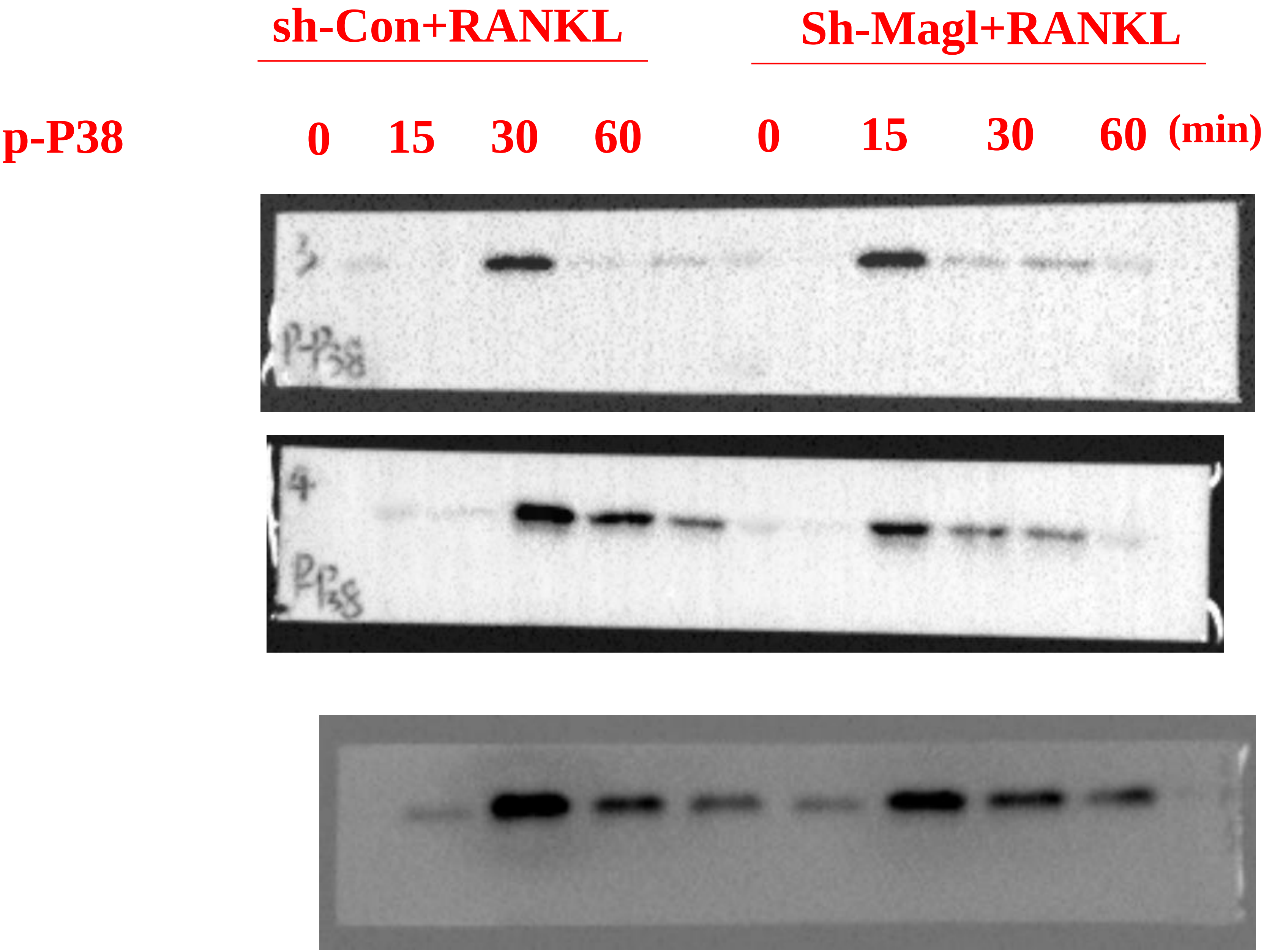
sh-Con+RANKL
Sh-Magl+RANKL
p-P38
0 15 30 60
0 15 30 60 (min)

sh-Con+RANKL Sh-Magl+RANKL

P38 0 15 30 60 0 15 30 60 (min)

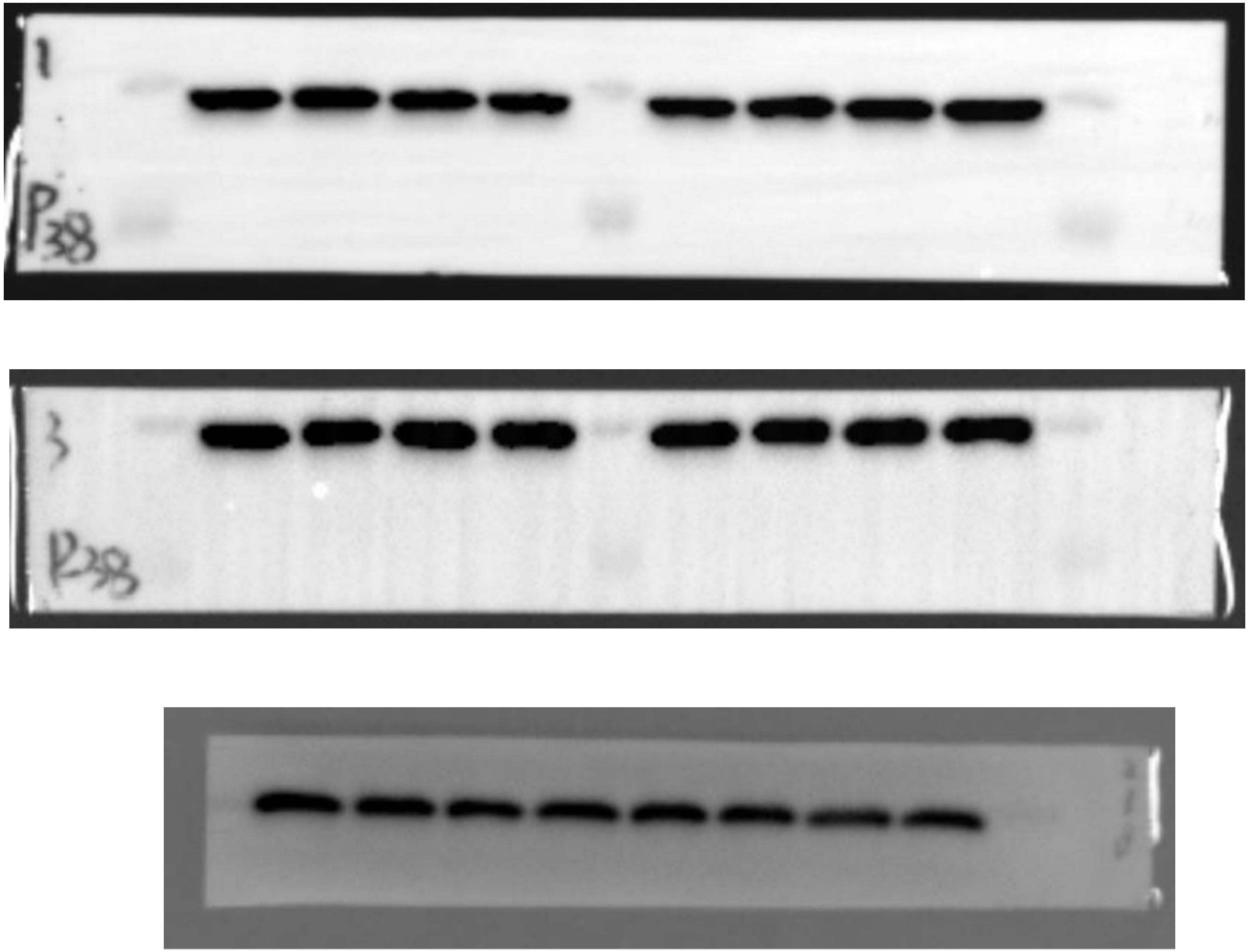

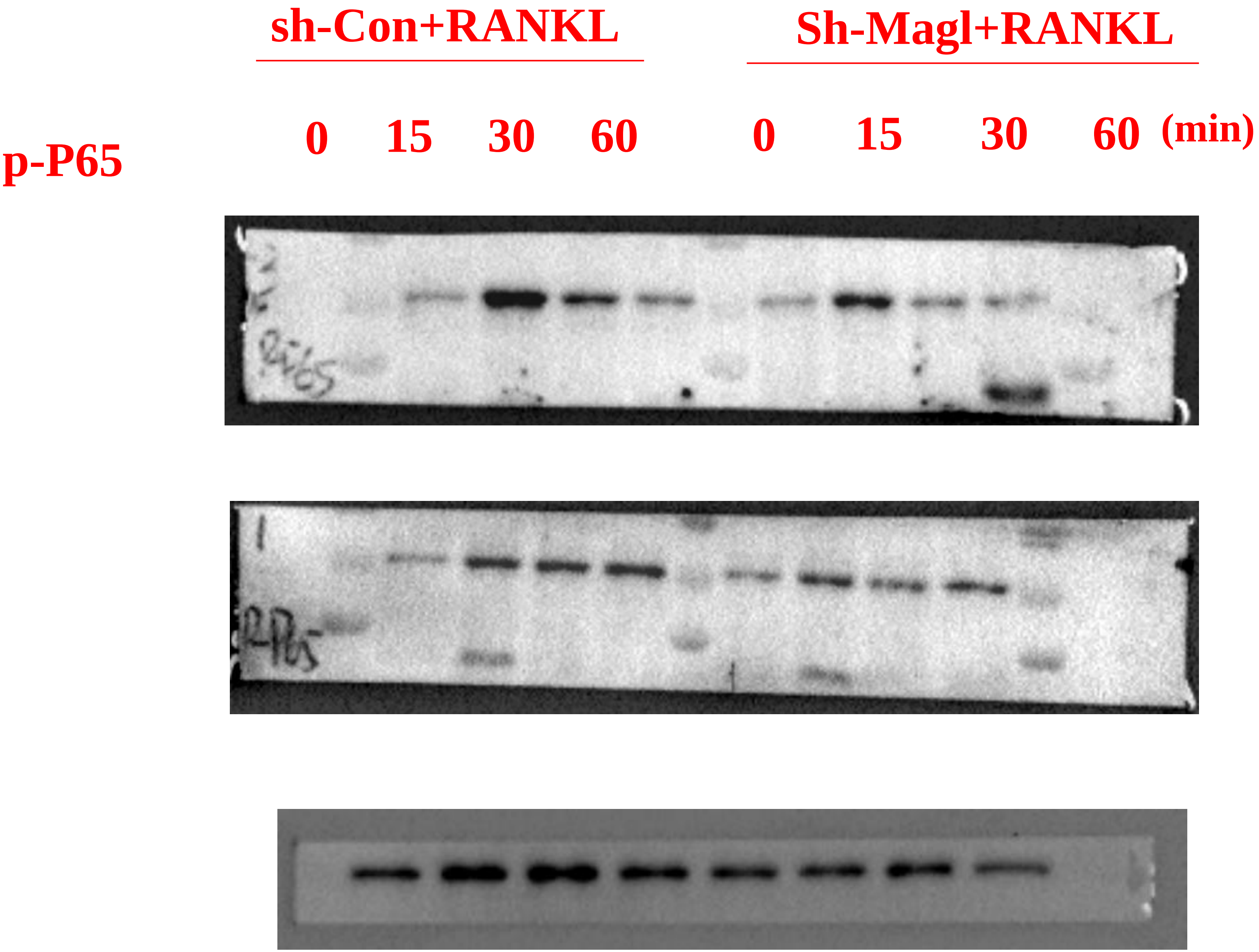
sh-Con+RANKL
Sh-Magl+RANKL
0 15 30 60 0 15 30 60 (min)
p-P65

	sh-Con+RANKL				Sh-Magl+RANKL				
P65	0	15	30	60	0	15	30	60	(min)

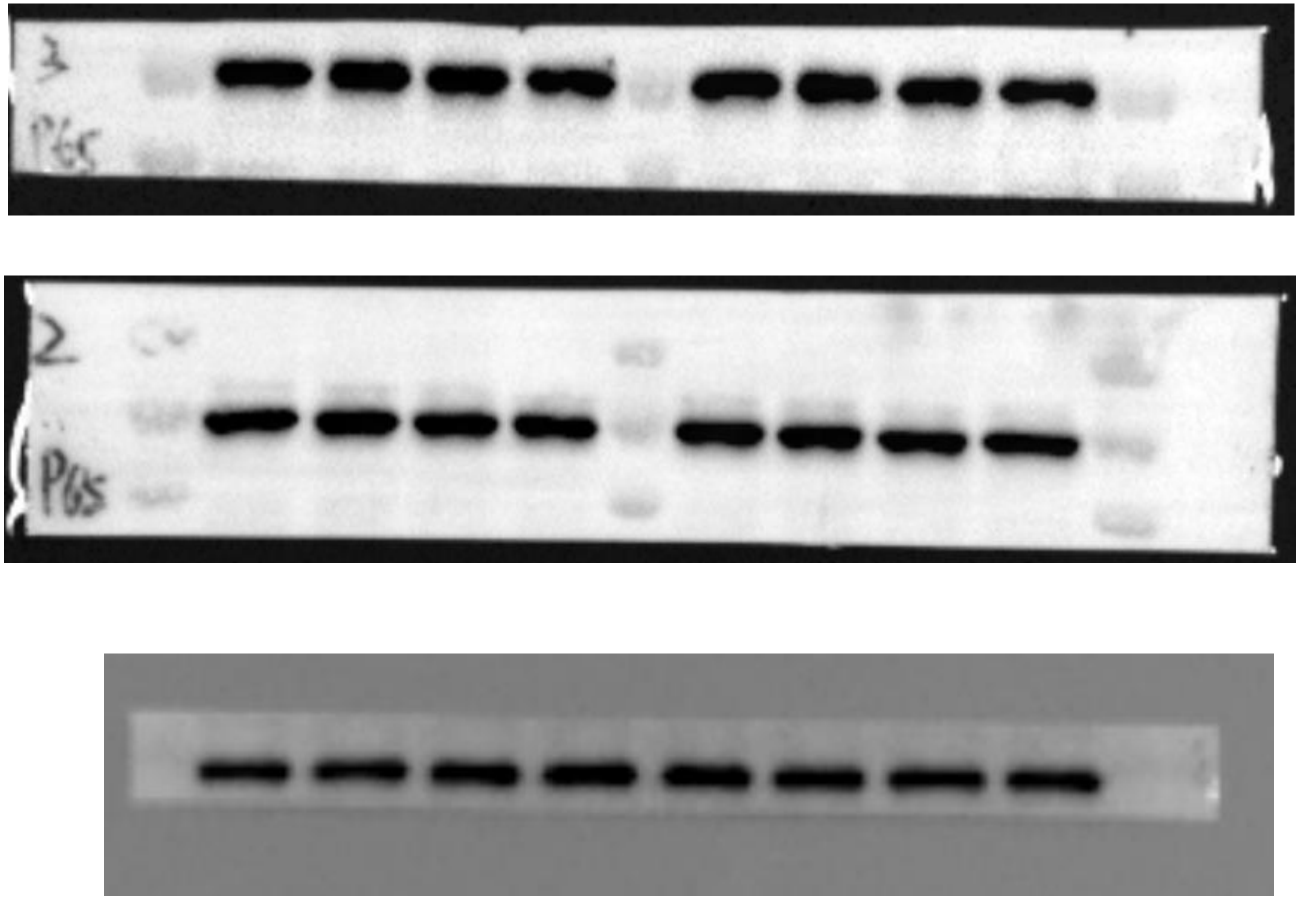

sh-Con+RANKL | Sh-Magl+RANKL

P-Akt 0 15 30 60 0 15 30 60 (min)

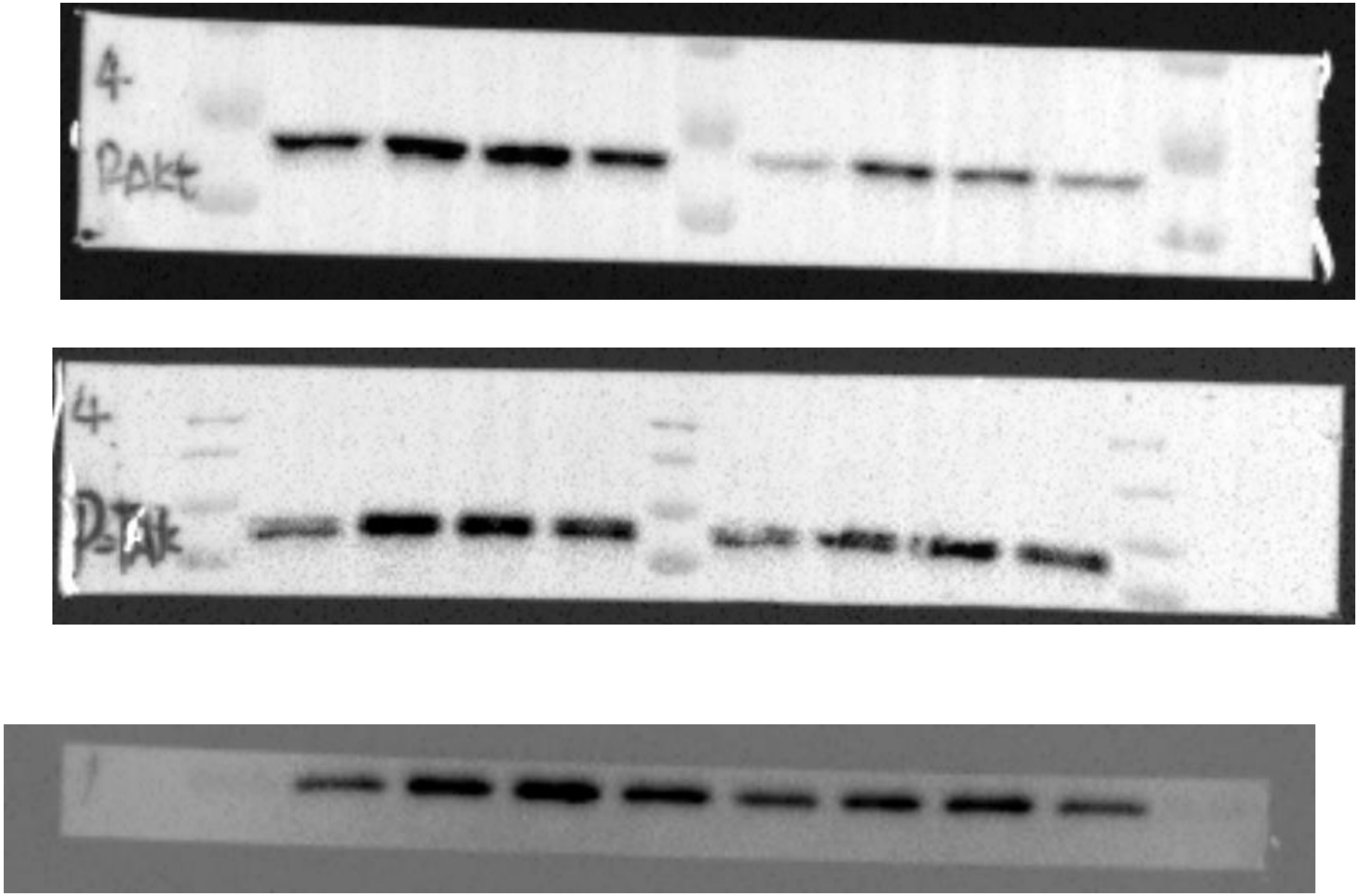

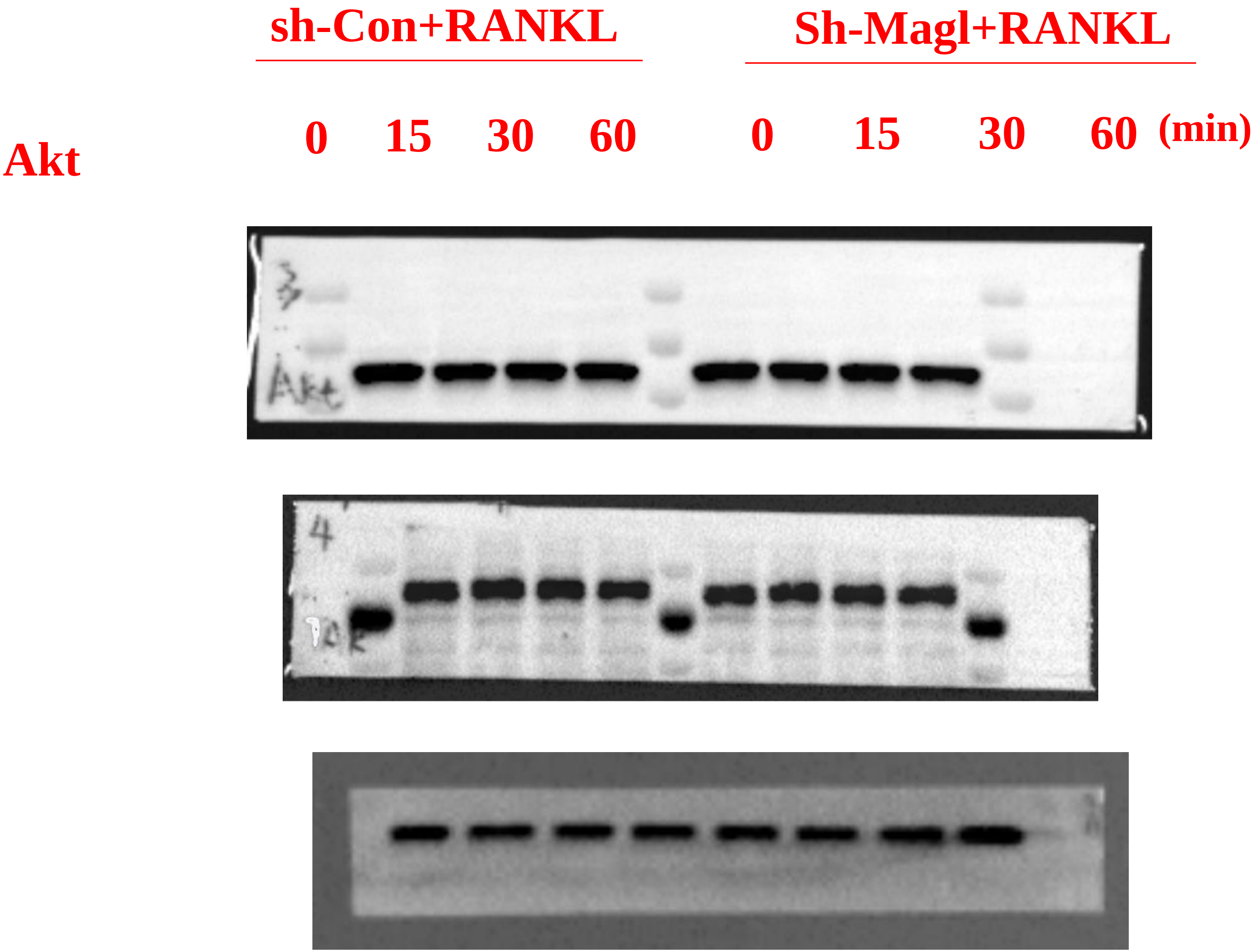
sh-Con+RANKL
Sh-Magl+RANKL
0 15 30 60 0 15 30 60 (min)
Akt

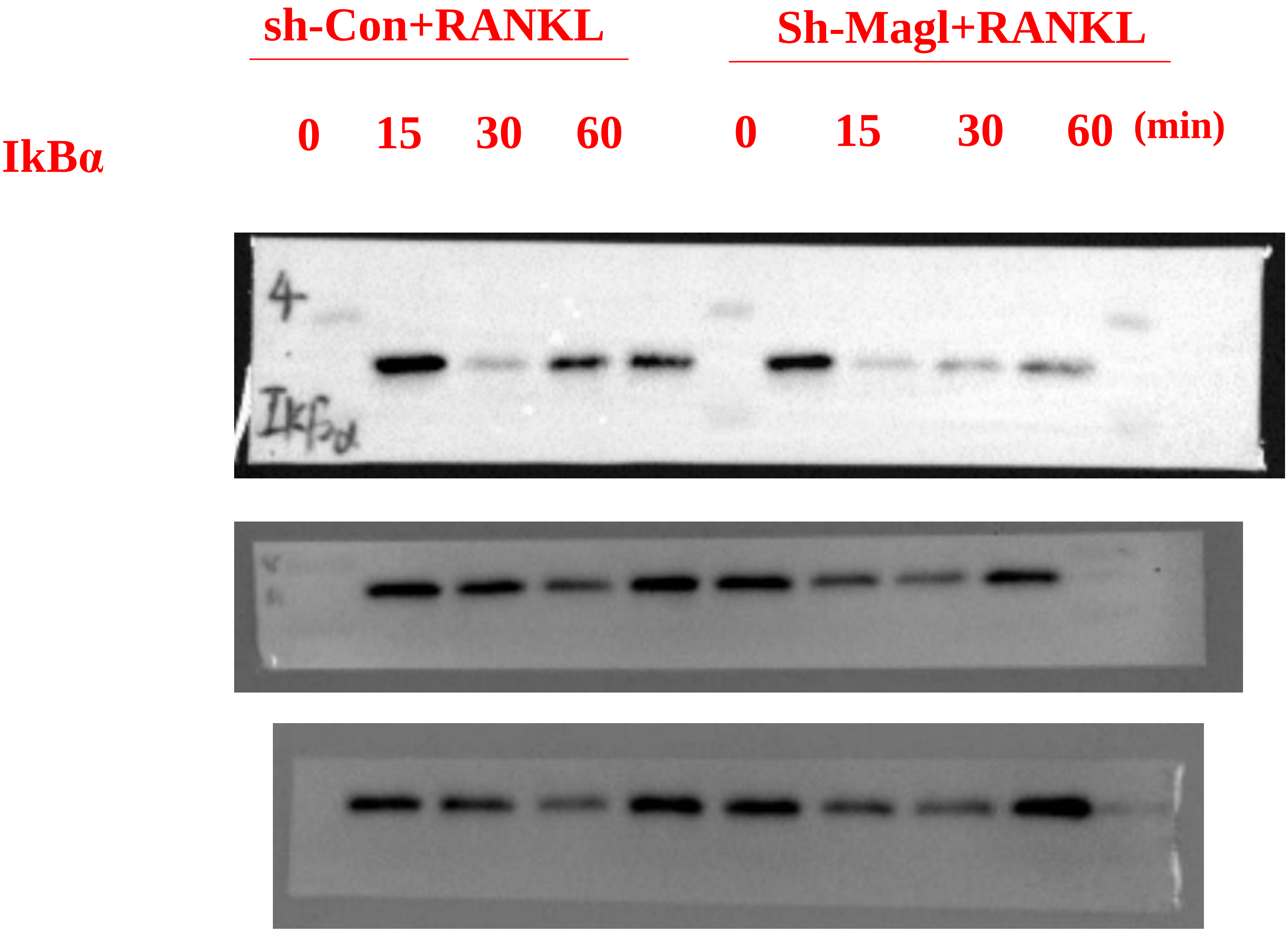
sh-Con+RANKL
Sh-Magl+RANKL
0 15 30 60 0 15 30 60 (min)
IkBα

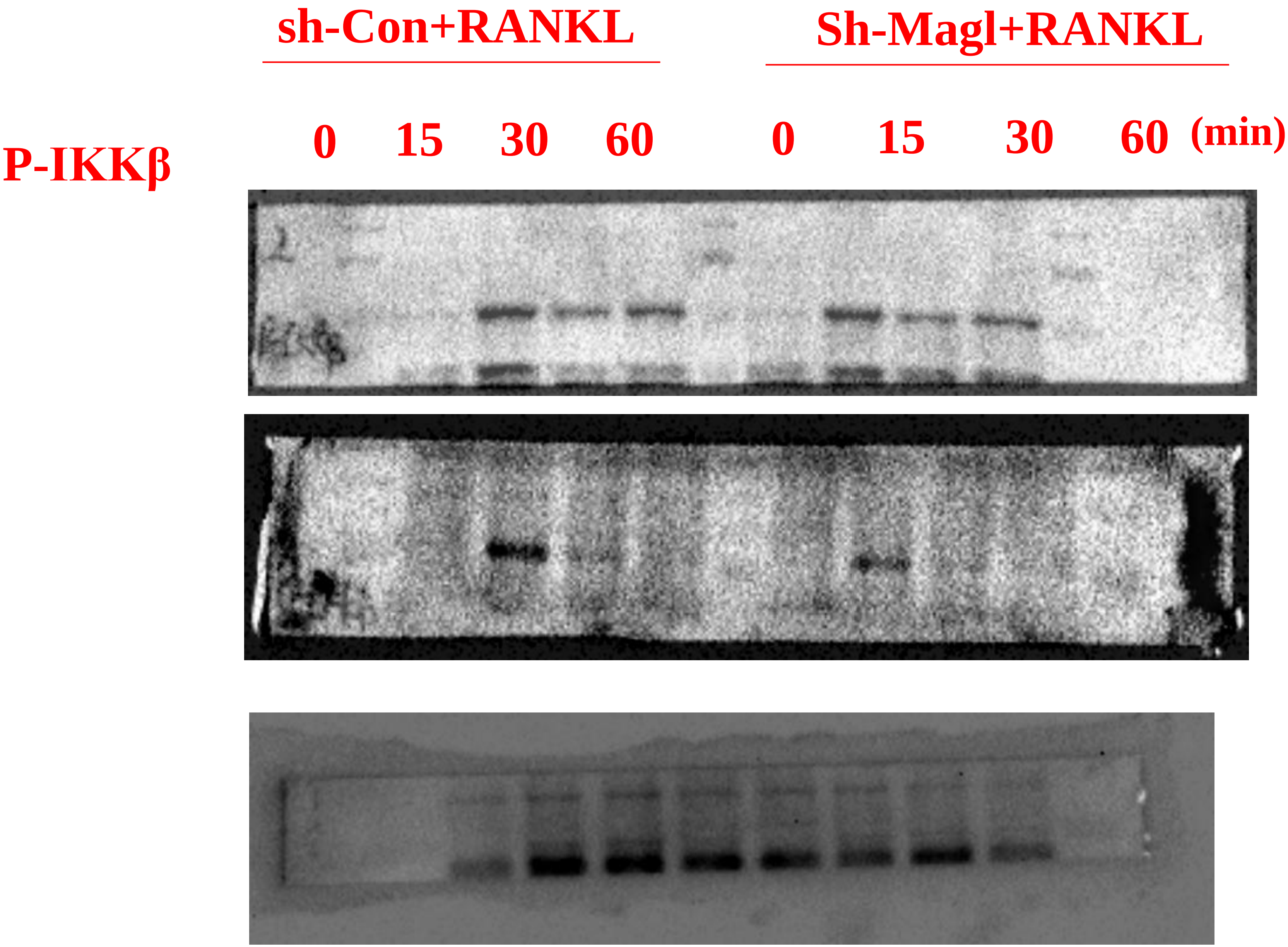
sh-Con+RANKL
Sh-Magl+RANKL
0 15 30 60
0 15 30 60 (min)
P-IKKβ

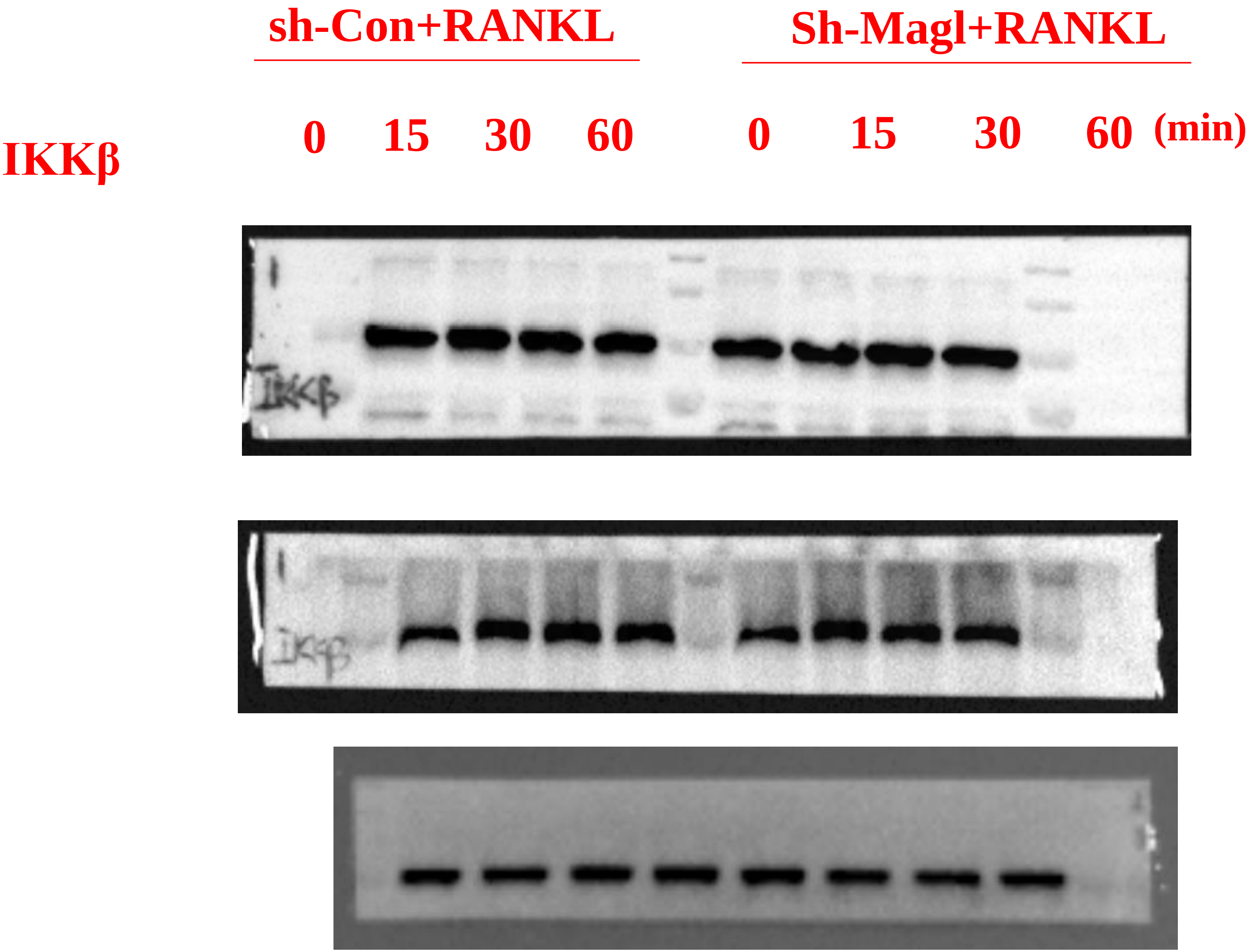
sh-Con+RANKL
Sh-Magl+RANKL
IKKβ
0
15
30
60
0
15
30
60
(min)

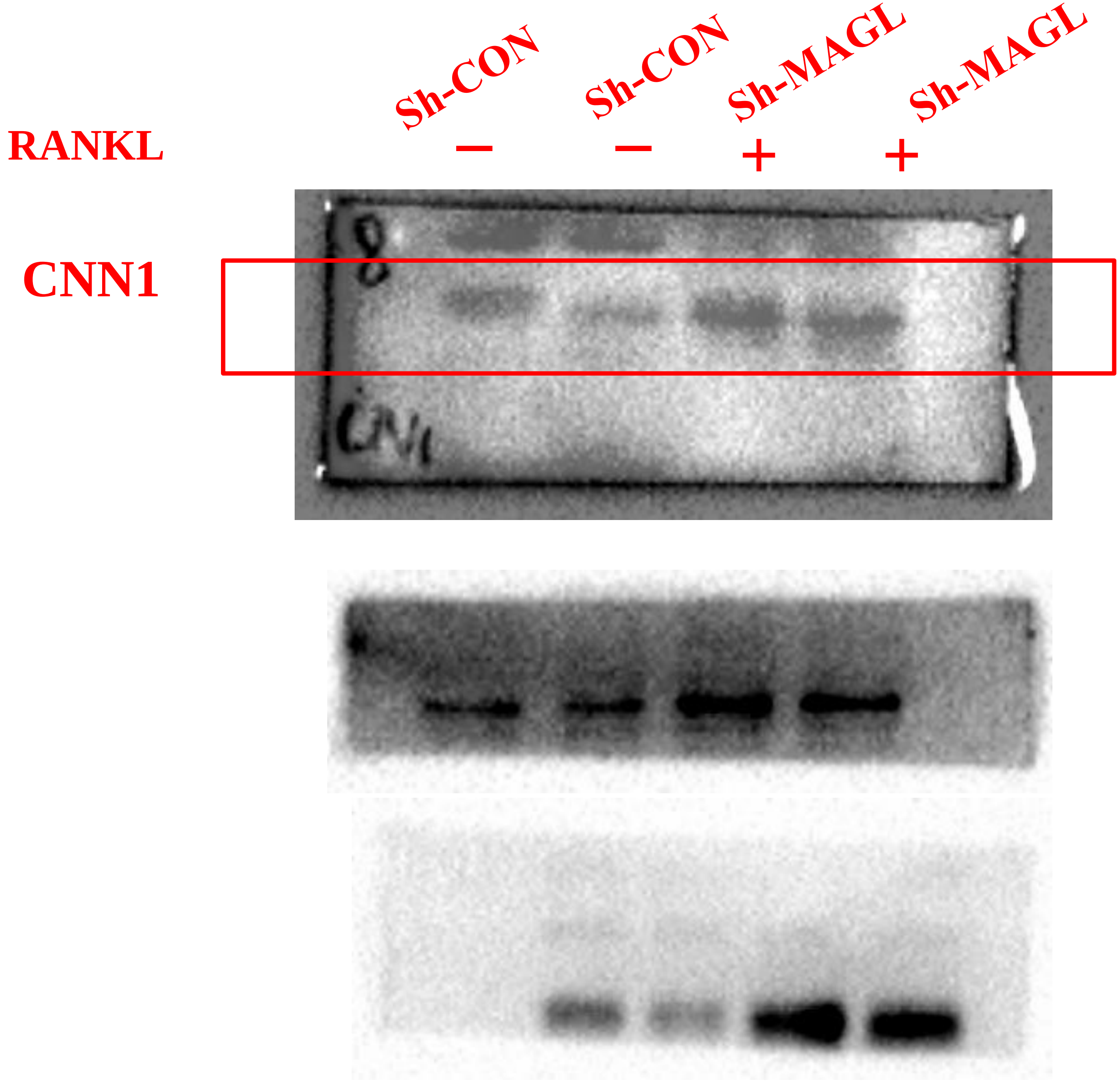
Sh-CON
Sh-CON
Sh-MAGL
Sh-MAGL
RANKL
−
−
+
+
CNN1

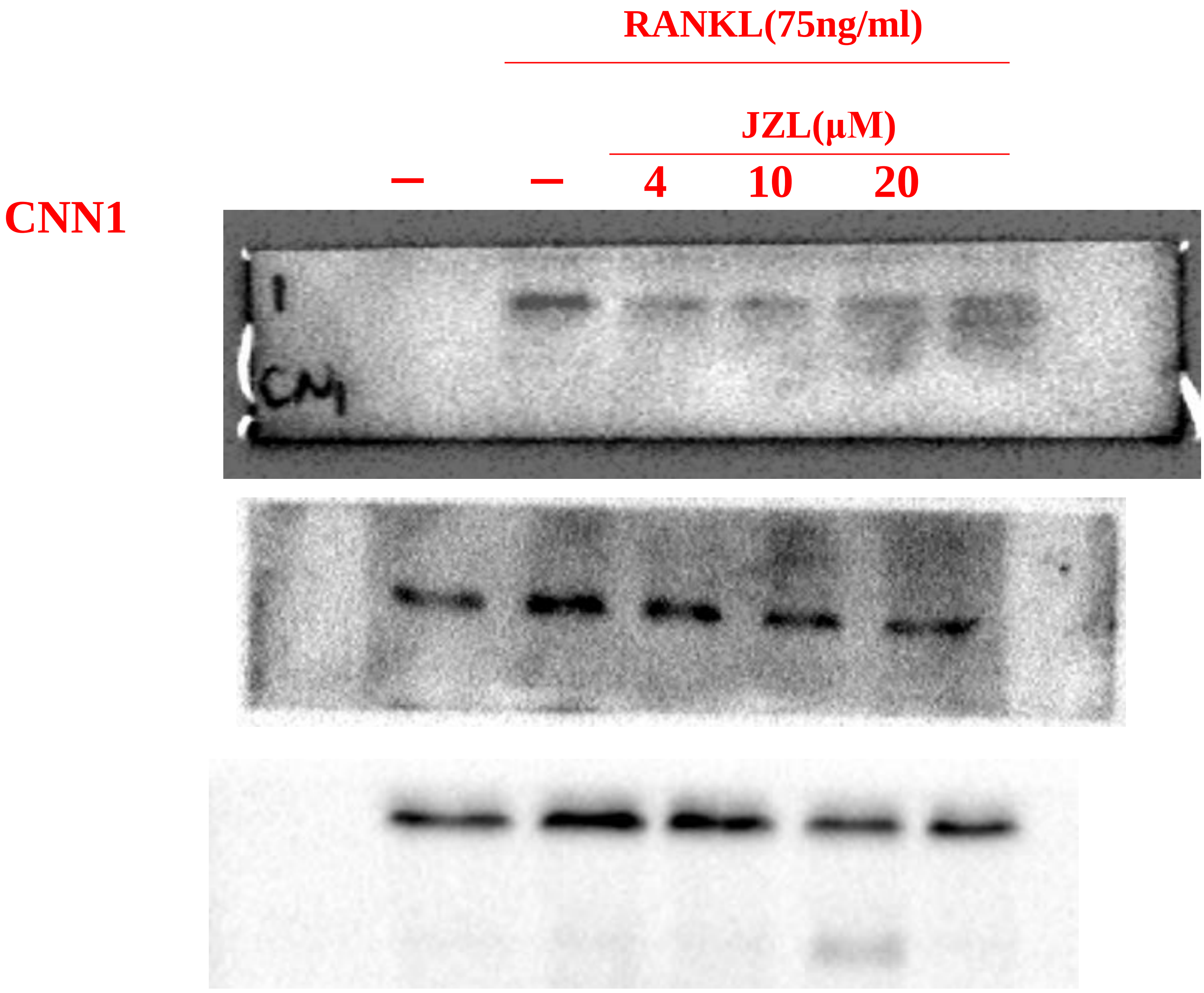
RANKL(75ng/ml)
JZL(μM)
–
–
4
10
20
CNN1
