# Supplementary material for: Inhibiting Monoacylglycerol Lipase Suppresses RANKL-Induced Osteoclastogenesis and Alleviates Ovariectomy-Induced Bone Loss
Source: Front Cell Dev Biol. 2021 Mar 12;9:640867. doi: 10.3389/fcell.2021.640867 (PMC7994615; doi:10.3389/fcell.2021.640867)
Supplement: Supplementary file 1 [file Data_Sheet_1.zip › Rename our supplementary files/Supplemental Table 1.The sequences for each shRNA of MAGL..docx]

Supplemental Table 1. The sequences for each shRNA were as follows:

| Target | Sequences |
| --- | --- |
| shRNA1 | 5’-GCGAACTCCACAGAATGTTCCTTCAAGAGAGGAACATTCTGTGGAGTTCGCTTTTTT -3’ |
| shRNA2 | 5’-GCTGGACATGCTGGTATTTGCTTCAAGAGAGCAAATACCAGCATGTCCAGCTTTTTT -3’ |
| shRNA3 | 5’-GAAGGTCCTTGCTGCCAAACTTTCAAGAGAAGTTTGGCAGCAAGGACCTTCTTTTTT-3’ |
| shRNA4 | 5’-GCCTACCTGCTCATGGAATCATTCAAGAGATGATTCCATGAGCAGGTAGGCTTTTTT -3’ |
